# Multimodal targeting of metastatic renal cell carcinoma via CD70-directed allogeneic CAR-NKT cells

**DOI:** 10.1016/j.xcrm.2025.102321

**Published:** 2025-08-29

**Authors:** Yan-Ruide Li, Junhui Hu, Zhe Li, Enbo Zhu, Yuning Chen, Tyler Halladay, Xinyuan Shen, Ying Fang, Yichen Zhu, Zibai Lyu, Yanxin Tian, Jie Huang, Annabel S. Zhao, Nathan Y. Ma, Catherine Zhang, Yongpeng Xie, Hanwei Zhang, Tzung Hsiai, Arnold I. Chin, Lily Wu, Lili Yang

**Affiliations:** 1 Department of Microbiology, Immunology & Molecular Genetics, University of California, Los Angeles (UCLA), Los Angeles, CA 90095, USA; 2 Department of Bioengineering, UCLA, Los Angeles, CA 90095, USA; 3 Department of Molecular and Medical Pharmacology, UCLA, Los Angeles, CA 90095, USA; 4 Division of Cardiology, Department of Medicine, David Geffen School of Medicine, UCLA, Los Angeles, CA 90095, USA; 5 Department of Urology, The First Affiliated Hospital of Chongqing Medical University, Chongqing 400016, China; 6 Department of Urology, David Geffen School of Medicine, UCLA, Los Angeles, CA 90095, USA; 7 Eli and Edythe Broad Center of Regenerative Medicine and Stem Cell Research, UCLA, Los Angeles, CA 90095, USA; 8 Jonsson Comprehensive Cancer Center, David Geffen School of Medicine, UCLA, Los Angeles, CA 90095, USA; 9 Institute of Urologic Oncology, UCLA, Los Angeles, CA 90095, USA; 10 Molecular Biology Institute, UCLA, Los Angeles, CA 90095, USA; 11 Parker Institute for Cancer Immunotherapy, UCLA, Los Angeles, CA 90095, USA; 12 Goodman-Luskin Microbiome Center, UCLA, Los Angeles, CA 90095, USA

**Keywords:** Renal cell carcinoma, RCC, CD70, invariant natural killer T cell, NKT cell, CAR-NKT cell, hematopoietic stem and progenitor cell, HSPC, tumor microenvironment, TME, alloreactive T cell, allorejection, patient-derived xenograft model, PDX, multimodal targeting

## Abstract

Renal cell carcinoma (RCC) represents about 90% of kidney cancers, with 30%–40% of patients developing metastatic disease despite current treatments. Conventional chimeric antigen receptor (CAR)-T therapy targeting CD70 shows promise but faces challenges due to its autologous, personalized nature. Here, we develop allogeneic CD70-directed CAR-engineered invariant natural killer T (^Allo^CAR70-NKT) cells from hematopoietic stem and progenitor cells using a clinically guided culture method. These cells expand robustly with high purity and no fratricide risk. ^Allo^CAR70-NKT cells exhibit potent cytotoxicity against primary and metastatic RCC via CAR- and natural killer (NK) receptor-mediated mechanisms and selectively target the immunosuppressive tumor microenvironment (TME) through T cell receptor (TCR) recognition. Additionally, they eliminate CD70^+^ host alloreactive T cells, promoting therapeutic persistence. Taken together, our findings support the therapeutic potential of ^Allo^CAR70-NKT cells as a next-generation, off-the-shelf immunotherapy with dual tumor- and TME-targeting functionality and the added capacity to eliminate alloreactive T cells, which offers a compelling strategy for treating metastatic RCC.

## Highlights

• Profiling primary RCC samples reveals therapeutic potential for CAR-NKT cells
• Generating ^Allo^CAR70-NKT cells with high yield, purity, and no fratricide risk
• ^Allo^CAR70-NKT cells deploy multimodal targeting against RCC tumors and the TME
• ^Allo^CAR70-NKT cells are safe and overcome host T cell-mediated allorejection

## Graphical abstract

**Figure:**
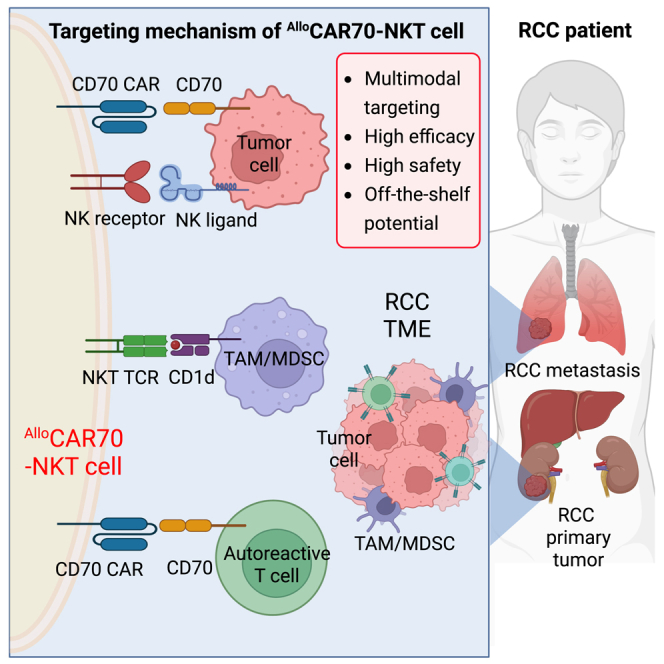

## Teaser

Li et al. generate allogeneic CD70-directed CAR-engineered NKT (^Allo^CAR70-NKT) cells from hematopoietic stem and progenitor cells using a clinically guided culture method. These cells exhibit multimodal targeting of renal cell carcinoma (RCC) tumor cells, the tumor microenvironment, and alloreactive T cells, representing a promising approach for metastatic RCC therapy.

## Introduction

Renal cell carcinoma (RCC), originating from renal epithelium, is the most prevalent type of kidney cancer, accounting for over 90% of all cases.^1^ RCC exhibits marked resistance to conventional chemotherapy and radiotherapy, underscoring the need for alternative therapeutic strategies.^2^ Although targeted therapies such as vascular endothelial growth factor (VEGF) and mammalian target of rapamycin inhibitors have improved clinical outcomes, approximately 33% of patients still progress to metastatic disease, with a 5-year survival of only 12%.^3^^,^^4^ These limitations highlight the urgent need for more effective and innovative treatment options.

Immunotherapy has emerged as a promising strategy for RCC, which is characterized by high levels of immune cell infiltration and responsiveness to immuno-oncology approaches.^4^ These insights have led to major clinical advancements, including the approval of high-dose interleukin (IL)-2 and immune checkpoint inhibitors targeting PD-1 and CTLA-4.^4^^,^^5^ Building upon these successes, chimeric antigen receptor (CAR)-engineered T (CAR-T) cell therapy targeting CD70 is emerging as an attractive approach for RCC.^6^^,^^7^ This involves genetically modifying T cells to express a CD70-directed CAR, typically constructed using a single-chain variable fragment or a truncated form of CD27 receptor, the binding partner of CD70.^6^^,^^7^ CD70 is a tumor-associated antigen expressed in over 80% of RCC tumors, with limited expression in normal tissues.^8^^,^^9^ Clinical trials investigating CD70-directed CAR-T (CAR70-T) cell therapies in RCC are currently underway.^9^^,^^10^

Despite their therapeutic potential, current CAR70-T cell therapies face several key limitations. Clinical responses have been modest, likely due to the inherent challenges posed by solid tumors, including antigen heterogeneity and a highly immunosuppressive tumor microenvironment (TME).^9^^,^^10^^,^^11^ Moreover, most CAR70-T cell products are autologous, necessitating patient-specific manufacturing, which is labor intensive, time consuming, and financially burdensome.^9^^,^^10^^,^^12^^,^^13^^,^^14^^,^^15^ These logistical hurdles restrict broad patient access and complicate the implementation of combination strategies, an approach that may be crucial for effectively treating metastatic RCC (mRCC).^9^^,^^10^^,^^16^ To overcome these limitations, the development of potent, off-the-shelf CAR70-based cell therapies that can address RCC tumor immune evasion and TME-associated suppression is critically needed.

Recent advances in allogeneic cell therapies offer encouraging prospects. For instance, CD70-directed CAR-engineered natural killer (CAR70-NK) cells derived from induced pluripotent stem cells (iPSCs) have demonstrated efficacy across various malignancies including RCC while also eliminating CD70-expressing alloreactive T cells.^17^ This approach of depleting alloreactive T cells is particularly beneficial, as it can enhance therapeutic cell persistence by mitigating host-mediated rejection.^17^ While it is a viable and promising approach, several challenges remain. CD70 must be genetically knocked out to prevent fratricide and cellular exhaustion of iPSC-derived CAR70-NK cells, thus complicating the manufacturing process.^17^ Additionally, the RCC TME contains an abundance of immunosuppressive tumor-associated macrophages (TAMs) and myeloid-derived suppressor cells (MDSCs) that pose a significant barrier to the persistence and function of therapeutic cells.^5^^,^^18^^,^^19^^,^^20^ Therefore, there is a pressing need for therapeutic modalities capable of targeting both RCC tumor cells and their TME.

To address these challenges, we employed our previously established hematopoietic stem and progenitor cell (HSPC) gene engineering technology and a clinically guided culture method to generate allogeneic CD70-directed CAR-engineered invariant natural killer T (^Allo^CAR70-NKT) cells.^21^ Through the use of a comprehensive array of experimental models, including primary RCC patient samples, patient-derived tumor cell lines, *in vitro* functional assays, and both orthotopic and metastatic *in vivo* xenograft models, we demonstrate that ^Allo^CAR70-NKT cells effectively and concurrently eliminate RCC tumor cells, modulate the immunosuppressive TME, and deplete CD70-expressing alloreactive T cells. These findings underscore the robust therapeutic efficacy and translational promise of ^Allo^CAR70-NKT cells as a next-generation, off-the-shelf cell therapy platform for the treatment of mRCC.

## Results

### Profiling of primary RCC patient samples reveals the therapeutic opportunities for CAR-NKT cell therapy

**Figure 1:**
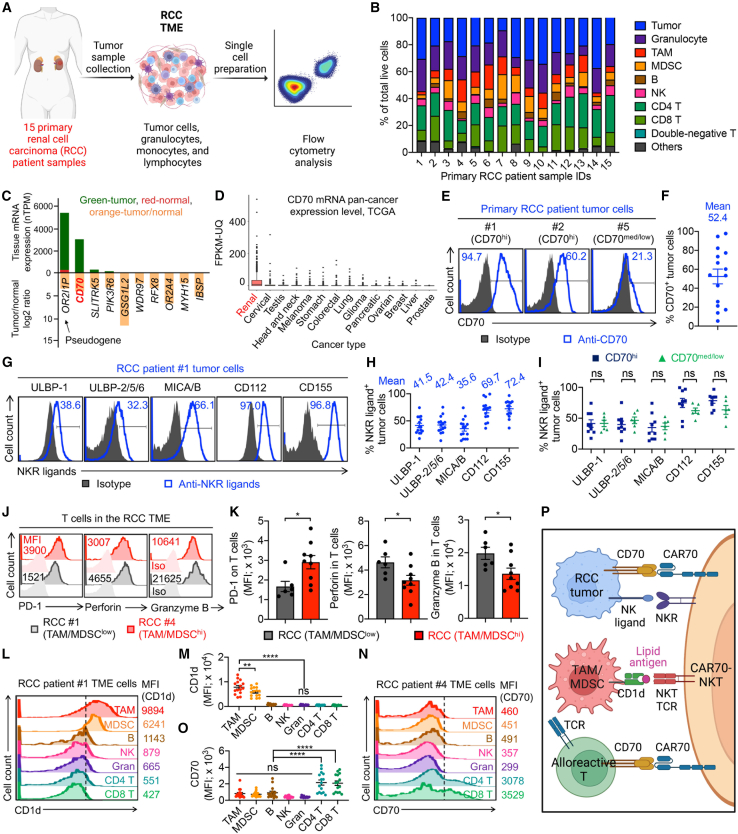
Biomarker profiling of primary RCC patient samples reveals therapeutic potential for CAR-NKT cell treatment (A) Experiment design to profile primary RCC patient tumor samples using flow cytometry. 15 RCC primary samples were included for analyses. (B) Bar graphs showing the cell cluster proportions of the 15 primary RCC tumor samples. (C) Comparison of mRNA expression levels in RCC tumor cells versus normal kidney tissues (upper bars) and corresponding log2 fold change between tumor and normal tissues (lower bars). Data were obtained from The Human Protein Atlas (HPA). nTPM, normalized transcript per million. (D) CD70 mRNA expression in various human cancer types. Raw data were collected from the Genomic Data Commons (GDC). FPKM-UQ, fragments per kilobase of transcript per million mapped reads-upper quartile. (E–I) Biomarker profiling of RCC tumor cells. (E) Fluorescence-activated cell sorting (FACS) detection of CD70 expression on tumor cells. Data from three representative samples are shown. CD70^hi^ indicates high (≥50%) CD70 expression; CD70^med/low^ indicates medium to low (<50%) CD70 expression. (F) Quantification of CD70 expression among the 15 primary RCC tumor cell samples. (G) FACS detection of NK ligand expression on tumor cells. Data from one representative sample are shown. (H) Quantification of NK ligand expression among the 15 primary RCC tumor cell samples. (I) Comparison of NK ligand expression between CD70^hi^ (*n* = 9) and CD70^med/low^ (*n* = 6) tumor cell samples. (J–O) Biomarker profiling of RCC TME cells. (J) FACS detection of PD-1 expression and Granzyme B and Perforin production in T cells. TAM/MDSC^low^, samples with <20% TAM/MDSCs among total immune cells; TAM/MDSC^high^, samples with >20% TAM/MDSCs among total immune cells; Iso, isotype staining. (K) Comparison of PD-1 expression and Granzyme B and Perforin production in T cells between TAM/MDSC^low^ (*n* = 6) and TAM/MDSC^high^ (*n* = 9) RCC samples. (L) FACS detection of CD1d expression on the indicated RCC TME cells. (M) Quantification of (L) (*n* = 15). (N) FACS detection of CD70 expression on the indicated RCC TME cells. (O) Quantification of (N) (*n* = 15). (P) Schematics showing the multimodal targeting of RCC tumor, microenvironment, and alloreactive T cells by CAR70-NKT cells. Data are presented as the mean ± SEM. ns, not significant; ∗*p* < 0.05, ∗∗*p* < 0.01, and ∗∗∗∗*p* < 0.0001 by Student’s t test (I and K) or one-way ANOVA (M and O).

To characterize RCC and identify optimal therapeutic strategies, we performed flow cytometry analyses on 15 primary RCC patient-derived tumor samples (Figure 1A). These specimens encompassed a range of pathological subtypes, disease stages, and anatomical locations, thereby representing the heterogeneity of RCC (Table S1). This analysis enabled the profiling of tumor cells and various immune cell populations within the RCC TME (Figure 1A).

Flow cytometry revealed a complex cellular composition across the RCC samples, including tumor cells, granulocytes, lymphocytes (e.g., T, B, and natural killer [NK] cells), and immunosuppressive populations such as TAMs and MDSCs (Figures 1B and S1A). Notably, we observed considerable inter-patient heterogeneity in the composition of tumor and TME cell populations (Figure 1B).

We first focused on the expression of CAR target antigens on RCC tumor cells. Among these, CD70 was found to be highly expressed on tumor cells while being largely absent in normal tissues (Figure 1C). Analysis of gene expression datasets confirmed that CD70 is not only differentially upregulated in RCC but also exhibits the highest expression levels in RCC among solid tumors (Figure 1D). Flow cytometry validated the high, albeit heterogeneous, expression of CD70 on RCC tumor cells across different patients, supporting its utility as a promising CAR target (Figures 1E and 1F). Additionally, RCC tumor cells exhibited universal and elevated expression of NK cell-activating ligands, including MHC class I chain-related protein A and B (MICA/B) and UL16-binding proteins (ULBPs) (ligands for NKG2D), as well as CD112 and CD155 (ligands for DNAM-1) (Figures 1G and 1H). Importantly, even RCC samples with low CD70 expression retained high levels of NK ligands (Figure 1I), suggesting susceptibility to natural killer receptor (NKR)-mediated cytotoxicity despite potential resistance to CAR-mediated killing.

We next assessed the immunosuppressive landscape of the RCC TME. All samples contained abundant TAMs and MDSCs (Figure 1B), which are known to facilitate tumor progression and impede immunotherapeutic responses.^11^^,^^22^^,^^23^ RCC samples enriched for TAMs and MDSCs displayed compromised T cell functionality, characterized by elevated expression of immune checkpoint molecules such as PD-1 and reduced production of cytotoxic effectors, including Perforin and Granzyme B (Figures 1J and 1K). Notably, both TAMs and MDSCs exhibited high expression of CD1d, a non-polymorphic molecule capable of presenting glycolipid antigens to the NKT T cell receptor (TCR),^24^^,^^25^^,^^26^ suggesting they could be selectively targeted by NKT cells (Figures 1L and 1M).^27^^,^^28^^,^^29^

Furthermore, we observed that T cells within the RCC TME, including both CD4^+^ and CD8^+^ subsets, expressed elevated levels of CD70 compared to other immune cell types, rendering them potential targets for CD70-directed CAR therapies (Figures 1N and 1O).

Together, these findings underscore the therapeutic potential of ^Allo^CAR70-NKT cells in treating RCC (Figure 1P). This platform leverages a multimodal mechanism of action: killing tumor cells via CAR and NKRs (e.g., NKG2D and DNAM-1), modulating the immunosuppressive TME via NKT TCR-mediated recognition of CD1d^+^ TAMs and MDSCs, and targeting CD70^+^ alloreactive T cells via CAR, thereby reducing allorejection (Figure 1P). The ability of ^Allo^CAR70-NKT cells to engage multiple tumor and TME components highlights their promise as an effective allogeneic cellular immunotherapy for RCC.

### Generate HSPC-engineered ^Allo^CAR70-NKT cells with high yield, purity, and no fratricide risk

**Figure 2:**
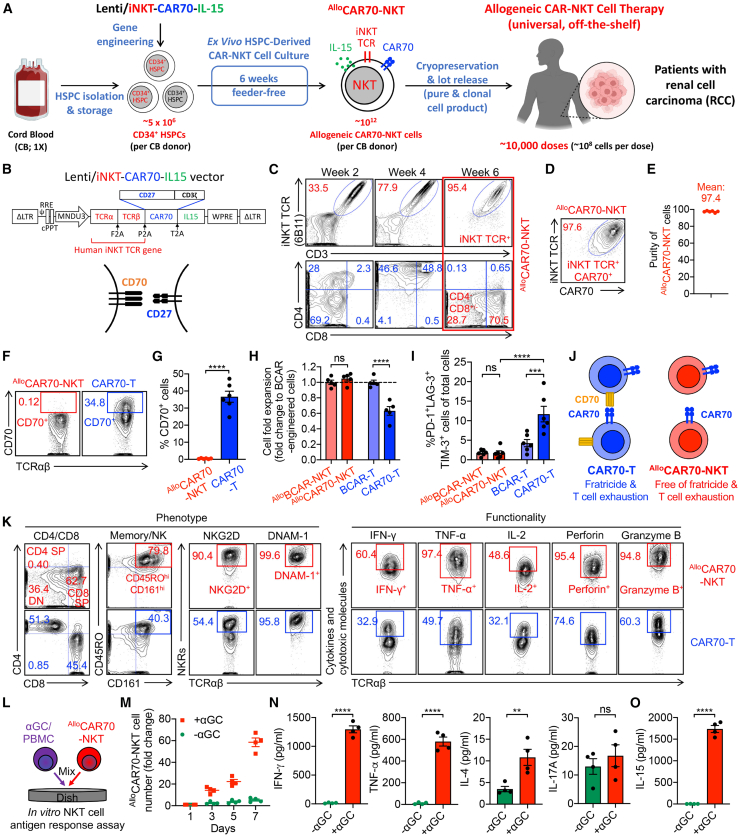
Generate HSPC-engineered ^Allo^CAR70-NKT cells with high yield, high purity, and no fratricide risk (A and B) Schematics showing the generation of ^Allo^CAR70-NKT cells (A) and the design of Lenti/iNKT-CAR70-IL-15 lentivector (B). (C) Fluorescence-activated cell sorting (FACS) monitoring of the generation and CD4/CD8 expression of ^Allo^CAR70-NKT cells during the 6-week culture. (D) FACS detection of CAR70 on ^Allo^CAR70-NKT cells. CAR70 was stained using an anti-CD27 monoclonal antibody. (E) Purity of ^Allo^CAR70-NKT cells (*n* = 6; n indicates different CB donors). (F–J) Study the fratricide risk of ^Allo^CAR70-NKT cells. (F) FACS detection of CD70 on ^Allo^CAR70-NKT cells. Healthy donor PBMC-derived conventional CAR70-T cells were included as a control. (G) Quantification of (F) (*n* = 6). (H) Fold expansion of the indicated cells normalized to their BCMA-targeting CAR (BCAR)-engineered counterparts (*n* = 5). (I) Quantification of PD-1^+^LAG-3^+^TIM-3^+^ cells as a percentage of the total cell population. (J) Schematics showing the absence of CD70 expression on ^Allo^CAR70-NKT cells, demonstrating resistance to fratricide and reduced T cell exhaustion. (K) FACS detection of surface marker expression, as well as intracellular cytokine and cytotoxic molecule production of ^Allo^CAR70-NKT and conventional CAR70-T cells. (L–O) Antigen responses of ^Allo^CAR70-NKT cells. ^Allo^CAR70-NKT cells were stimulated with/without αGC-loaded PBMCs for 1 week. (L) Experimental design. (M) Growth curve of ^Allo^CAR70-NKT cells (*n* = 4). (N) ELISA measurements of effector cytokine levels in the culture supernatants collected on day 5 (*n* = 4). (O) ELISA measurements of IL-15 levels in the culture supernatants collected at 48 h (*n* = 4). Representative of over 5 experiments. Data are presented as the mean ± SEM. ns, not significant; ∗*p* < 0.05, ∗∗*p* < 0.01, ∗∗∗*p* < 0.001, and ∗∗∗∗*p* < 0.0001 by Student’s t test (G, H, N, and O) or one-way ANOVA (I).

We employed a previously established platform to generate ^Allo^CAR70-NKT cells by genetically engineering the NKT TCR into human HSPCs and differentiating them using a clinically guided, feeder-free *ex vivo* culture system (Figure 2A). CD34^+^ HSPCs derived from human cord blood (CB) were cultured in a five-stage, 6-week *ex vivo* differentiation protocol to generate ^Allo^CAR70-NKT cells (Figure 2A). HSPCs were transduced using a lentiviral vector encoding: (1) a pair of NKT TCR α and β chains; (2) a CAR construct targeting CD70, utilizing the natural CD70 ligand CD27 as the binding domain^30^^,^^31^; and (3) soluble human IL-15, known to enhance *in vivo* persistence and functionality, as demonstrated in clinical trials using GD2-directed CAR-NKT cells for neuroblastoma (Figure 2B).^32^^,^^33^

Flow cytometry analysis confirmed the successful generation and progressive enrichment of ^Allo^CAR70-NKT cells, with NKT cells increasing from ∼10% in week 1 to >99% by week 6 (Figures 2C and S1B). These cells underwent a characteristic NKT developmental trajectory based on CD4/CD8 co-receptor expression, transitioning from double-negative (DN) to double-positive (DP), and eventually differentiating into CD8 single-positive (SP) or DN subsets (Figure 2C).^34^^,^^35^ By the end of the culture, >97% of the cells expressed CAR70, owing to the co-expression of the CAR and TCR in the same lentiviral vector, obviating the need for additional CAR^+^ cell enrichment and making the product amenable to off-the-shelf use (Figures 2D, 2E, and S1C). Notably, from a single CB donor with ∼5 × 10^6^ CD34^+^ HSPCs, it is feasible to generate up to ∼10^12 Allo^CAR70-NKT cells, theoretically yielding 1,000–10,000 therapeutic doses (based on conventional CAR-T infusion doses of 10^8^–10^9^ cells) (Figures S1D and S1E).^36^^,^^37^^,^^38^

To benchmark the performance of ^Allo^CAR70-NKT cells, we compared them with healthy donor PBMC-derived conventional CAR70-T cells (Figures S2A and S2B). CAR70-T cells typically exhibited ∼80% CAR transduction efficiency (Figures S2C–S2E). However, because conventional T cells endogenously express CD27 (used as the CAR70-binding domain), CD27-based detection does not accurately reflect CAR expression (Figure S2C). To address it, we co-expressed EGFP as a CAR marker in CAR70-T cells (Figures S2D and S2E). Despite achieving high CAR70 expression, CAR70-T cell manufacturing was subject to variability, requiring additional purification steps that could affect consistency.^39^^,^^40^ In contrast, ^Allo^CAR70-NKT cells consistently achieved nearly 100% CAR70 expression, eliminating the need for enrichment and ensuring uniform CAR expression across the product.

Targeting CD70 with CAR-T cells presents a unique challenge: upon activation, conventional T cells upregulate CD70, making them susceptible to CAR-mediated fratricide, which can drive cell exhaustion.^30^^,^^41^^,^^42^ This has emerged as a critical limitation in current CAR70-T cell therapies under clinical evaluation. Remarkably, ^Allo^CAR70-NKT cells did not express CD70, thus avoiding fratricide and the associated exhaustion phenotype (Figures 2F and 2G). When compared with our previously developed B cell maturation antigen (BCMA)-targeted ^Allo^BCAR-NKT cells,^21 Allo^CAR70-NKT cells showed comparable yield and similarly low expression of exhaustion markers (i.e., PD-1, TIM-3, and LAG-3) (Figures 2H–2J). In contrast, conventional CAR70-T cells exhibited reduced yields and elevated exhaustion marker expression compared to their BCMA-targeted counterparts (Figures 2H–2J).

These results highlight the advantages of ^Allo^CAR70-NKT cells as a fratricide-free, exhaustion-resistant, and scalable off-the-shelf cellular therapy for targeting CD70-expressing cancers such as RCC, without the need for CD70 knockout engineering (Figure 2J).^43^

### ^Allo^CAR70-NKT cells display typical NKT cell phenotype and functionality

Flow cytometry analysis revealed that ^Allo^CAR70-NKT cells predominantly exhibited CD8 SP and DN phenotypes, with an absence of CD4 SP cells, a notable distinction from endogenous human NKT cells and conventional CAR70-T cells, which often contain a significant CD4 SP subset (Figure 2K).^32^^,^^33^^,^^44^ Functional characterization showed that both CD8 SP and DN ^Allo^CAR70-NKT cells exhibited comparable pro-inflammatory and cytotoxic profiles, attributes that are highly favorable for cancer immunotherapy (Figure 2K).^45^^,^^46^^,^^47^

In comparison to conventional CAR70-T cells, ^Allo^CAR70-NKT cells displayed a typical NKT cell phenotype, characterized by high expression of T cell memory marker CD45RO and multiple activating NKRs, including CD161, NKG2D, and DNAM-1 (Figures 2K and S2F). Functionally, these cells produced elevated levels of pro-inflammatory cytokines (i.e., interferon [IFN]-γ, tumor necrosis factor alpha [TNF-α], and IL-2) and cytotoxic effector molecules (i.e., Perforin and Granzyme B), supporting a robust antitumor potential consistent with their CD8 SP and DN phenotypes (Figures 2K and S2F).

To confirm the functional integrity of the transduced NKT TCR, ^Allo^CAR70-NKT cells were stimulated with the canonical NKT cell agonist, α-galactosylceramide (αGC) (Figure 2L). Upon stimulation, the cells exhibited vigorous proliferation and secreted high levels of Th1-associated cytokines (IFN-γ and TNF-α), while producing only minimal amounts of Th2 (IL-4) and Th17 (IL-17A) cytokines (Figures 2M, 2N, and S2G). This Th1-skewed cytokine profile aligns with the cytotoxic and inflammatory phenotype of CD8 SP/DN cells and is desirable for anti-cancer activity (Figure 2K). Additionally, robust secretion of IL-15 following αGC stimulation confirmed successful transgene expression of human IL-15, which is included in the lentiviral construct to enhance *in vivo* persistence and function (Figures 2O and S2H).^48^^,^^49^^,^^50^

### ^Allo^CAR70-NKT cells exhibit potent antitumor activity and engage multiple tumor-targeting mechanisms against RCC tumor cells *in vitro*

**Figure 3:**
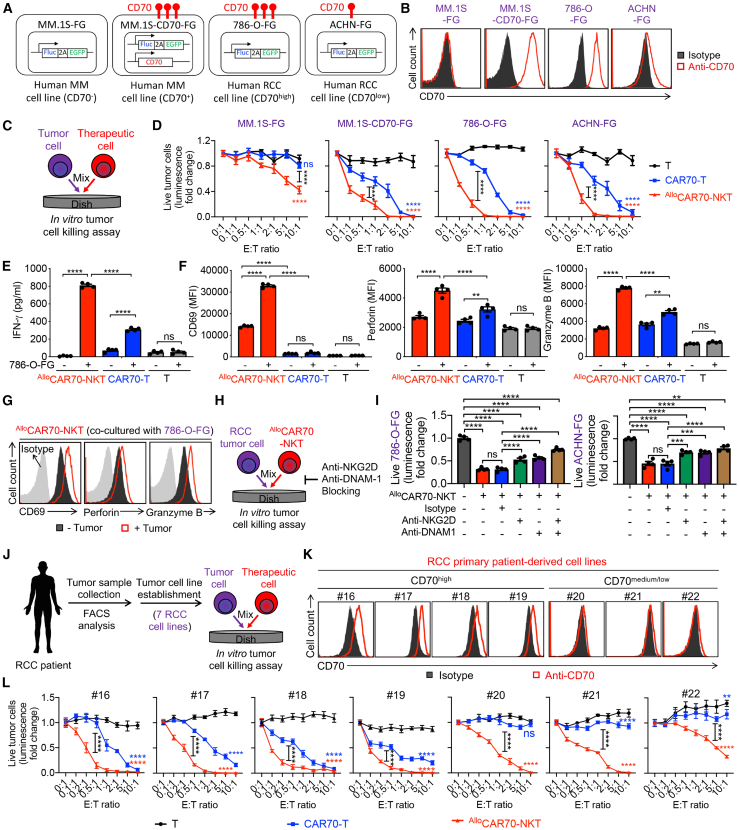
^Allo^CAR70-NKT cells exhibit potent antitumor activity and engage multiple tumor-targeting mechanisms against RCC tumor cells (A–G) Studying the *in vitro* antitumor efficacy of ^Allo^CAR70-NKT cells against human RCC cell lines. (A) Schematics showing the indicated human tumor cell lines. (B) FACS detection of CD70 expression on the indicated tumor cell lines. (C) Experimental design. (D) Tumor cell killing data at 24 h (*n* = 4). (E) ELISA analyses of IFN-γ production by the indicated therapeutic cells (*n* = 4). E:T, effector to tumor. (F) FACS analyses of surface CD69 expression as well as intracellular Perforin and Granzyme B production in the indicated therapeutic cells (*n* = 4). (G) FACS detection of effector molecule expression in ^Allo^CAR70-NKT cells with or without co-culture with 786-O-FG tumor cells. (H and I) Studying the tumor cell killing mechanisms of ^Allo^CAR70-NKT cells mediated by NKRs. (H) Experimental design. (I) Tumor cell killing data at 24 h (E:T ratio = 0.5:1; *n* = 4). (J–L) Studying the *in vitro* antitumor efficacy of ^Allo^CAR70-NKT cells against primary RCC patient-derived tumor cell lines. (J) Experimental design. (K) FACS detection of CD70 expression on the indicated RCC patient-derived cell lines. (L) Tumor cell killing data at 24 h (*n* = 4). Representative of 3 experiments. Data are presented as the mean ± SEM. ns, not significant; ∗*p* < 0.05, ∗∗*p* < 0.01, ∗∗∗*p* < 0.001, and ∗∗∗∗*p* < 0.0001 by one-way ANOVA (E, F, and I) or two-way ANOVA (D and L).

We then assessed the *in vitro* cytotoxic potential of ^Allo^CAR70-NKT cells against RCC using a series of tumor cell killing assays, employing both stable tumor cell lines (Figures 3A–3I) and primary RCC patient-derived tumor cell lines (Figures 3J–3L). These targets were selected to represent diverse tumor scenarios, with varying expression of surface CAR target antigens (i.e., CD70) and NKR ligands (e.g., MICA/B, ULBPs, CD112, and CD155), thus modeling RCC-associated tumor heterogeneity. All tumor cell lines were engineered to stably express firefly luciferase and green fluorescent protein dual reporters (FG) for quantifiable tracking via bioluminescence and flow cytometry (Figure 3A). Three therapeutic cell types were evaluated, including ^Allo^CAR70-NKT cells, conventional CAR70-T, and non-transduced PBMC-derived T cells.

We first utilized five human tumor cell lines exhibiting variable CD70 expression (Figures 3A and S3A). These included two NK-resistant multiple myeloma cell lines, MM.1S-FG (CD70^−^) and MM.1S-CD70-FG (CD70^+^), chosen to assess CAR-mediated cytotoxicity. Additionally, three RCC lines were used: 786-O-FG (CD70^high^), ACHN-FG (CD70^low^), and a CRISPR-Cas9-engineered CD70 knockout line, 786-O-FG^CD70−/−^ (Figures 3B and S3B). These RCC lines enabled evaluation of both CAR- and NKR-mediated killing. PBMC-T cells exhibited no cytotoxicity against any target cells within 24 h (Figures 3C, 3D, and S3C). In contrast, CAR70-T cells efficiently lysed CD70^+^ targets while showing no activity against CD70^−^ cells, demonstrating strict CAR70-CD70 dependency (Figures 3D and S3C). Strikingly, ^Allo^CAR70-NKT cells were capable of killing both CD70^+^ and CD70^−^ tumor cells, with enhanced efficacy toward CD70^+^ targets, indicating both CAR-dependent and CAR-independent mechanisms (Figures 3D and S3C). Tumor cell killing was accompanied by elevated expression of activation markers (i.e., CD69) and robust production of effector cytokines (i.e., IFN-γ) and cytotoxic molecules (i.e., Perforin and Granzyme B) (Figures 3E–3G). Notably, cytotoxicity against RCC tumor cells was significantly reduced upon blockade of key NKRs (e.g., NKG2D and DNAM-1), confirming the contribution of NKR-mediated tumor recognition (Figures 3H and 3I). Moreover, simultaneous inhibition of both CAR and NKR pathways led to a more substantial decrease in cytotoxic activity, indicating that CAR and NKR signaling function additively to mediate the tumor-killing effects of ^Allo^CAR70-NKT cells (Figures S3D–S3F).

We further validated these findings using a panel of primary RCC patient-derived tumor lines, each with varying levels of CD70 expression (Figures 3J and 3K; Table S1). In CD70^high^ primary RCC lines, both CAR70-T and ^Allo^CAR70-NKT cells induced potent tumor cell killing, though ^Allo^CAR70-NKT cells demonstrated superior efficacy, likely due to their multifaceted tumor-targeting capabilities (Figure 3L). In contrast, CAR70-T cells exhibited diminished cytotoxicity against CD70^low^ RCC lines, whereas ^Allo^CAR70-NKT cells maintained robust killing capacity, reinforcing their ability to target tumors through CAR-independent mechanisms (Figure 3L).

Collectively, these data demonstrate that ^Allo^CAR70-NKT cells possess superior tumor-killing efficacy compared to conventional CAR70-T cells. Their dual-targeting capacity, mediated via both CAR70 and NKRs, enables them to effectively overcome tumor antigen heterogeneity and CAR target loss, key limitations observed in CAR-T cell therapies in both preclinical and clinical settings.^51^^,^^52^^,^^53^^,^^54^^,^^55^

### ^Allo^CAR70-NKT cells demonstrate superior antitumor efficacy, tumor homing, and enhanced effector/cytotoxic functions in an orthotopic RCC PDX model

**Figure 4:**
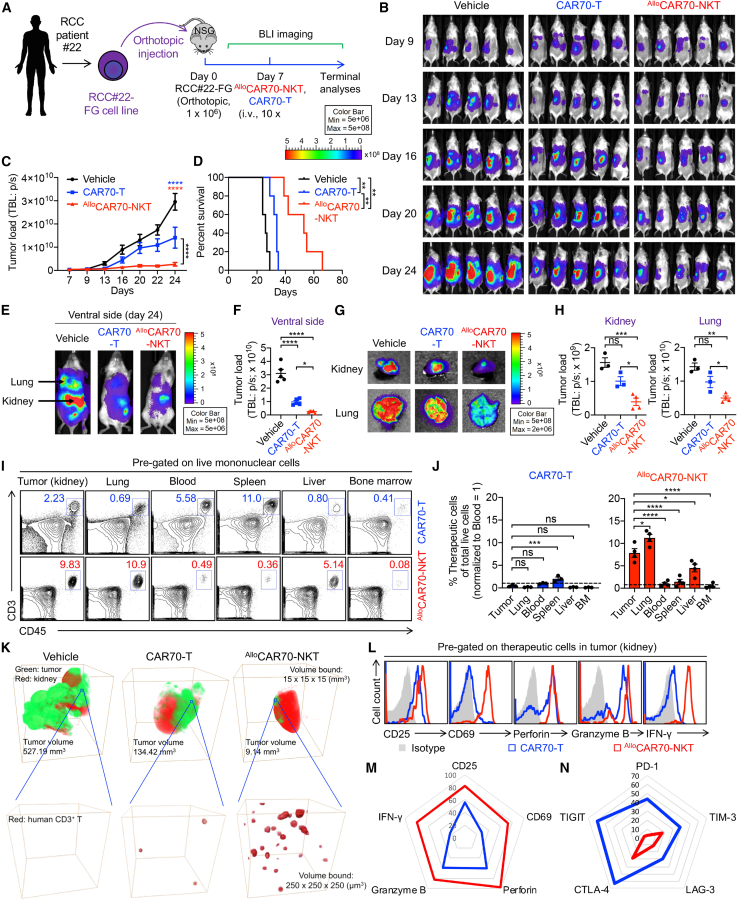
^Allo^CAR70-NKT cells demonstrate superior antitumor efficacy, tumor homing, and enhanced effector and cytotoxic functions in an RCC patient-derived xenograft orthotopic mouse model (A) Experimental design. BLI, bioluminescence imaging. (B) BLI images showing the presence of tumor cells in experimental mice over time. (C) Quantification of (B) (*n* = 5). (D) Kaplan-Meier survival curves of experimental mice over time (*n* = 5). (E) BLI images showing the biodistribution of RCC tumor cells in representative experimental mice. Ventral views are shown. (F) Quantification of (E) (*n* = 5). (G) Tissue BLI images showing the presence of RCC tumor cells in mouse kidneys and lungs on day 18. (H) Quantification of (G) (*n* = 3–4). (I) Fluorescence-activated cell sorting (FACS) detection of the presence of therapeutic cells in the indicated tissues of experimental mice on day 18. (J) Quantification of (I) (*n* = 3–4). The percentage of therapeutic cells among total live mononuclear cells was measured and normalized to the blood (set as 1). BM, bone marrow. (K) 3D tissue imaging of kidney samples collected from experimental mice on day 18. Upper panel: volume rendering of a 15 × 15 × 15 mm^3^ region showing GFP^+^ tumor cells (green) and GFP^−^ kidney tissue (red). Lower panel: high-resolution view of a 250 × 250 × 250 μm^3^ region showing tumor-infiltrating human CD3^+^ cells (red). (L) FACS detection of the effector molecule expression in therapeutic cells collected from tumor sites of experimental mice on day 18. (M and N) Radar plots showing the effector molecule (M) and immune checkpoint (N) expression in therapeutic cells (*n* = 3–4). Representative of 2 experiments. Data are presented as the mean ± SEM. ns, not significant; ∗*p* < 0.05, ∗∗*p* < 0.01, ∗∗∗*p* < 0.001, and ∗∗∗∗*p* < 0.0001 by one-way ANOVA (F, H, and J), two-way ANOVA (C), or log rank (Mantel-Cox) text adjusted for multiple comparisons (D).

**Figure 5:**
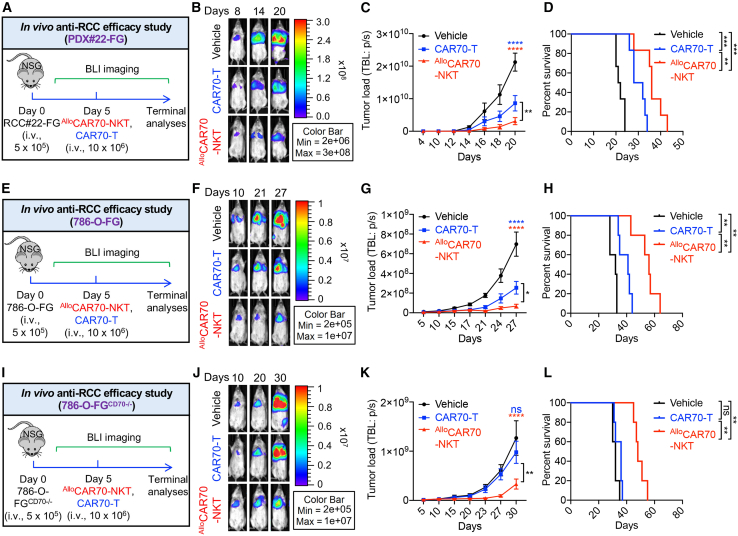
^Allo^CAR70-NKT cells elicit robust antitumor activity in human RCC metastatic xenograft mouse models (A–D) Studying the *in vivo* antitumor efficacy of ^Allo^CAR70-NKT cells in a human RCC PDX NSG mouse model. (A) Experimental design. (B) BLI images. (C) Quantification of (B) (*n* = 6). (D) Kaplan-Meier survival curves (*n* = 6). (E–H) Studying the *in vivo* antitumor efficacy of ^Allo^CAR70-NKT cells in a 786-O-FG human RCC xenograft NSG mouse model. (E) Experimental design. (F) BLI images. (G) Quantification of (F) (*n* = 5). (H) Kaplan-Meier survival curves (*n* = 5). (I–L) Studying the *in vivo* antitumor efficacy of ^Allo^CAR70-NKT cells in a 786-O-FG^CD70−/−^ human RCC xenograft NSG mouse model. (I) Experimental design. (J) BLI images. (K) Quantification of (J) (*n* = 5). (L) Kaplan-Meier survival curves (*n* = 5). Representative of 2 experiments. Data are presented as the mean ± SEM. ns, not significant; ∗*p* < 0.05, ∗∗*p* < 0.01, ∗∗∗*p* < 0.001, and ∗∗∗∗*p* < 0.0001 by one-way ANOVA (C, G, and K) or log rank (Mantel-Cox) text adjusted for multiple comparisons (D, H, and L).

To evaluate the *in vivo* antitumor efficacy of ^Allo^CAR70-NKT cells, we established a series of RCC patient-derived xenograft (PDX) models, including both orthotopic (Figure 4) and metastasis models (Figure 5). The orthotopic model recapitulates the native TME, preserving the anatomical, stromal, and vascular context of the kidney, while the metastasis model establishes the RCC cell dissemination to and colonization in the lungs, a common metastatic site in patients with advanced RCC.^56^^,^^57^

We first developed an orthotopic, spontaneous metastatic model of RCC by implanting tumor cells derived from a patient with advanced-stage RCC into the renal capsule of the kidney (Table S1; Figure 4A).^58^ In this model, a single administration of CAR70-T or ^Allo^CAR70-NKT cells resulted in significant tumor suppression; however, ^Allo^CAR70-NKT cells demonstrated greatly enhanced tumor control and prolonged survival of treated mice (Figures 4B–4D). In this aggressive metastatic model, spontaneous metastasis from the kidney to the lungs was observed as early as day 9 (Figure 4B) and extensively at day 24 (Figure 4E). Notably, compared to conventional CAR70-T cells, ^Allo^CAR70-NKT cells showed superior control of tumor growth at both primary (kidney) and metastatic (lung) sites, highlighting their capacity to target and eliminate both localized and disseminated RCC (Figures 4E–4H).

Tissue biodistribution analysis revealed distinct trafficking patterns between the two therapeutic cell types. CAR70-T cells primarily accumulated in peripheral lymphoid organs such as the blood and spleen, with minimal infiltration into tumor sites (Figures 4I and 4J). In contrast, ^Allo^CAR70-NKT cells preferentially homed to the major tumor-bearing organs of kidney and lungs, demonstrating superior tumor-homing capacity, a critical advantage for treating solid tumors (Figures 4I and 4J). Three-dimensional tissue imaging of mouse kidneys revealed that treatment with ^Allo^CAR70-NKT cells resulted in smaller residual tumor burden compared to conventional CAR70-T cell therapy, and a greater number of ^Allo^CAR70-NKT cells were observed infiltrating the solid tumor regions (Figure 4K).

To investigate the molecular basis of this differential trafficking, we performed chemokine receptor profiling. Compared to conventional CAR70-T cells, ^Allo^CAR70-NKT cells expressed higher levels of tumor-homing-associated chemokine receptors, including CCR2, CCR5, CCR6, CXCR3, and CXCR4, which are receptors known to mediate effector lymphocyte migration into inflamed and tumor-infiltrated tissues (Figures S4A and S4B).^59^ Interestingly, ^Allo^CAR70-NKT cells exhibited reduced expression of CCR7, a receptor involved in homing to secondary lymphoid organs (Figures S4A and S4B).^60^ This distinct chemokine receptor signature likely underlies the enhanced tumor infiltration observed with ^Allo^CAR70-NKT cells and may account for the limited tumor accumulation seen with CAR70-T cells, which remain largely confined to peripheral lymphoid compartments (Figures 4I and 4J).

Further phenotypic analysis of therapeutic cells infiltrating the kidney tumor revealed that ^Allo^CAR70-NKT cells exhibited a more activated and cytotoxic phenotype, characterized by elevated expression of activation markers (i.e., CD25 and CD69), pro-inflammatory cytokines (i.e., IFN-γ), and cytotoxic effectors (i.e., Granzyme B and Perforin) (Figures 4L and 4M). Moreover, these cells expressed significantly lower levels of exhaustion markers (i.e., PD-1, CTLA-4, TIM-3, LAG-3, and TIGIT), suggesting sustained effector functionality within the TME (Figure 4N).

Although ^Allo^CAR70-NKT cells demonstrated potent *in vivo* antitumor activity, tumor relapsed eventually, leading to mouse mortality. This may be attributed to the limited persistence of ^Allo^CAR70-NKT cells, which were detectable in peripheral blood for approximately 1 month post-infusion before declining (Figures S4C and S4D). To address this limitation, we conducted an additional *in vivo* efficacy study using the same orthotopic PDX model and implemented a repeated dosing strategy (Figure S4E). ^Allo^CAR70-NKT cells were administered intravenously on days 7, 14, and 21 following tumor inoculation. Notably, the repeated infusion led to improved tumor control and significantly prolonged survival in treated mice (Figures S4F–S4H). These findings suggest that, in the context of aggressive mRCC, multiple infusions of ^Allo^CAR70-NKT cells can enhance therapeutic efficacy. Furthermore, the off-the-shelf nature of these allogeneic cells makes manufacturing, banking, and repeated dosing clinically feasible.

Together, these findings demonstrate that ^Allo^CAR70-NKT cells possess enhanced tumor homing, potent effector function, and resilience against exhaustion in the TME, resulting in superior antitumor efficacy. These features underscore the therapeutic potential of ^Allo^CAR70-NKT cells as a promising candidate for the treatment of RCC, particularly in cases of metastatic disease.

### ^Allo^CAR70-NKT cells elicit robust antitumor activity in human RCC metastatic models

We then established a series of mRCC xenograft models using highly aggressive PDX#22 RCC cell line (Figure 5A),^58^ the 786-O-FG cell line (Figure 5E), and the 786-O-FG^CD70−/−^ cell line (Figure 5I), which represents a model of CAR70 antigen escape (Figures S3A–S3C). After intravenous administration, all three models established metastatic lesions, localized to the mouse lungs (Figures 5B, 5F, and 5J). Treatment with either conventional CAR70-T cells or ^Allo^CAR70-NKT cells suppressed tumor growth and prolonged mouse survival in the CD70-expressing model of PDX#22 and 786-O-FG, with ^Allo^CAR70-NKT cells demonstrating superior antitumor efficacy (Figures 5B–5D and 5F–5H).

Notably, in the CD70 null 786-O-FG^CD70−/−^ metastasis model, conventional CAR70-T cells failed to control tumor growth due to the absence of their target antigen (Figures 5J–5L). In contrast, ^Allo^CAR70-NKT cells retained the ability to eliminate tumor cells, primarily through NKR-mediated tumor recognition mechanisms (Figures 3H, 3I, and 5J–5L).

Together, these results from orthotopic and metastatic models highlight the superior antitumor capacity of ^Allo^CAR70-NKT cells, demonstrating their ability to target primary kidney tumors and metastatic lung lesions after systemic delivery. These findings position ^Allo^CAR70-NKT cells as a potent and versatile therapeutic platform for mRCC, particularly in tumors exhibiting low CAR antigen expression or immune evasion.

### ^Allo^CAR70-NKT cells effectively target the RCC TME by depleting immunosuppressive TAMs and MDSCs via CD1d-dependent recognition

**Figure 6:**
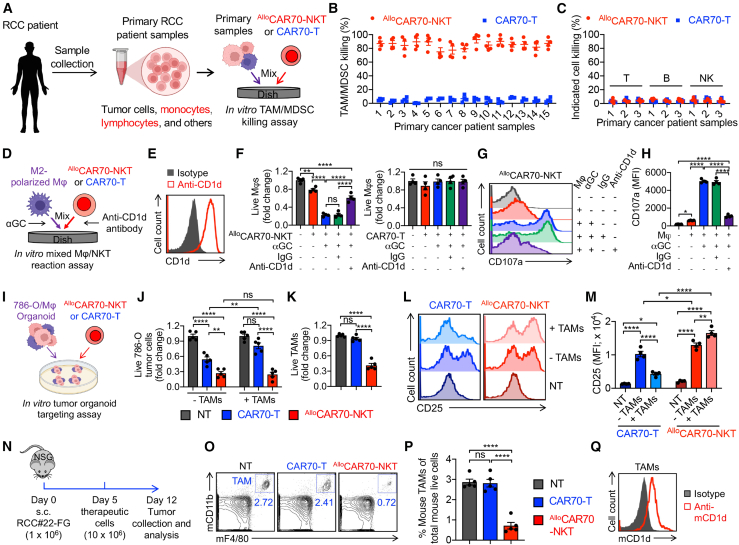
^Allo^CAR70-NKT cells effectively target the RCC TME through CD1d-mediated recognition (A–C) Studying ^Allo^CAR70-NKT cells targeting of RCC TME using primary RCC patient samples. (A) Experimental design. (B) TAM/MDSC killing data at 24 h (*n* = 4). (C) Killing data of the indicated RCC TME cell components at 24 h (*n* = 3). (D–M) Studying ^Allo^CAR70-NKT cells targeting of RCC TME using *in vitro*-cultured human M2-polarized macrophages and 786-O/macrophage co-culture organoid models. (D) Experimental design to study the direct killing to macrophages by ^Allo^CAR70-NKT cells. (E) Fluorescence-activated cell sorting (FACS) detection of CD1d expression on macrophages. (F) Macrophage killing data at 24 h (*n* = 4). (G) FACS detection of degranulating cytotoxic marker CD107a expression in ^Allo^CAR70-NKT cells. (H) Quantification of (G) (*n* = 4). (I) Experimental design to study killing to RCC tumor cells in the 786-O/macrophage organoids by ^Allo^CAR70-NKT cells. (J) Tumor cell killing data at 24 h (*n* = 4). (K) TAM killing data at 24 h (*n* = 4). (L) FACS detection of activation marker CD25 expression in the indicated therapeutic cells. (M) Quantification of (M) (*n* = 4). (N–Q) Studying ^Allo^CAR70-NKT cell targeting of RCC TME using a human RCC patient-derived xenograft (PDX) NSG mouse model. (N) Experimental design. (O) FACS detection of mouse TAMs (gated as mouse CD11b^+^F4/80^+^ cells) in tumors collected from experimental mice at the termination day. (P) Quantification of (O) (*n* = 4–5). (Q) FACS detection of mouse CD1d expression on the mouse TAMs. Representative of 3 experiments. Data are presented as the mean ± SEM. ns, not significant; ∗*p* < 0.05, ∗∗*p* < 0.01, and ∗∗∗∗*p* < 0.0001 by one-way ANOVA (F, H, J, K, M, and P).

The presence of immunosuppressive cells, including TAMs and MDSCs, within the TME significantly contributes to RCC resistance and evasion of current therapies.^23^ These immunosuppressive cells express high levels of CD1d, making them susceptible to NKT TCR-mediated recognition and killing (Figures 1L, 1M, and 1P). We therefore assessed the ability of ^Allo^CAR70-NKT cells to target and eliminate TAMs and MDSCs using primary RCC patient samples (Figures 6A–6C), a 786-O/TAM organoid model (Figures 6D–6M), and an *in vivo* 786-O human RCC xenograft model (Figures 6N–6Q).

In the first study, we co-cultured ^Allo^CAR70-NKT and CAR70-T cells with 15 primary RCC patient samples (Figure 6A). Unlike conventional CAR70-T cells, ^Allo^CAR70-NKT cells selectively and efficiently depleted CD1d^+^ TAMs and MDSCs, while sparing other immune populations expressing no or low levels of CD1d, including granulocytes, T cells, B cells, and NK cells (Figures 6B and 6C).

In the second study, we established RCC organoids by 3D-culturing 786-O tumor cells with M2-polarized macrophages derived from human monocytes to mimic TAMs (termed 786-O/TAM organoids).^19^^,^^61^ An *in vitro* cytotoxicity assay confirmed that ^Allo^CAR70-NKT cells could directly kill CD1d^+^ macrophages, whereas CAR70-T cells could not (Figures 6D–6F). The cytotoxicity of ^Allo^CAR70-NKT cells was enhanced by the NKT agonist αGC and attenuated by CD1d blockade, confirming TCR-CD1d-mediated killing (Figure 6F). This activity was supported by increased expression of effector markers (i.e., CD25), cytotoxic molecules (i.e., Granzyme B), and degranulation markers (i.e., CD107a) (Figures 6G, 6H, and S5A). We then evaluated tumor cell killing within the 786-O/TAM organoids (Figure 6I). In the absence of TAMs, both CAR70-T and ^Allo^CAR70-NKT cells killed the tumor cells, with ^Allo^CAR70-NKT cells showing superior cytotoxicity (Figure 6J). However, in the presence of TAMs, CAR70-T cell function was significantly impaired, highlighting TAM-mediated immunosuppression, while ^Allo^CAR70-NKT cells maintained robust tumor-killing activity (Figures 6J–6M and S5B). This suggests that ^Allo^CAR70-NKT cells can both overcome TAM-mediated suppression and eliminate TAMs, preserving their effector functions.

Furthermore, we established 786-O/TAM organoids under hypoxic conditions to better recapitulate the immunosuppressive TME (Figure S5C).^62^ Under these conditions, the antitumor activity of both conventional CAR70-T cells and ^Allo^CAR70-NKT cells was reduced compared to normoxic controls (Figures 6J and S5D). However, ^Allo^CAR70-NKT cells retained the ability to effectively kill both tumor cells and TAMs (Figures S5D and S5E). The presence of TAMs and hypoxia significantly impaired the Granzyme B production by conventional CAR70-T cells, whereas ^Allo^CAR70-NKT cells maintained robust Granzyme B production even under hypoxia (Figures S5F and S5G).

In the third study, we treated NOD scid gamma (NSG) mice bearing human RCC xenografts with ^Allo^CAR70-NKT or CAR70-T cells and analyzed the TME (Figure 6N). Although these mice lack human myeloid cells, the model retains murine myeloid populations that express mouse CD1d, an evolutionarily conserved molecule recognized by the human NKT TCR, allowing for the study of TME interactions (Figure S5H–S5K).^63^ Conventional CAR70-T cells did not reduce TAM levels within tumors, whereas ^Allo^CAR70-NKT cells led to a marked depletion of TAMs, consistent with the CD1d expression profile (Figures 6O–6Q).

Altogether, these results demonstrate that ^Allo^CAR70-NKT cells possess a dual capacity to target both RCC tumor cells and the immunosuppressive TME by eliminating CD1d^+^ TAMs and MDSCs. This makes ^Allo^CAR70-NKT cells a highly promising candidate for treating RCC, particularly in immune-evasive, TME-rich tumors.

### ^Allo^CAR70-NKT cells selectively target CD70^+^ alloreactive T cells leading to enhanced *in vivo* persistence

Host-mediated allorejection remains a major challenge for allogeneic cell therapies, as it can compromise *in vivo* persistence and therapeutic efficacy.^13^^,^^14^^,^^64^ Our HSPC-derived allogeneic CAR-NKT cells possess a stable, intrinsic hypoimmunogenic phenotype, characterized by low expression of human leukocyte antigen (HLA)-I/II and NK ligands, rendering them resistant to both host T and NK cell-mediated rejection.^21^^,^^65^ Furthermore, these cells can be engineered with *B2M* and *CIITA* knockouts to fully abrogate HLA-I/II expression, further mitigating host T cell-driven rejection.^66^ Given the high CD70 expression on activated host T cells (Figures 1N–1P), we hypothesized that ^Allo^CAR70-NKT cells are not only resistant to host T cell-mediated allorejection due to their hypoimmunogenic phenotype but can also selectively eliminate CD70^+^ alloreactive T cells, thereby reducing host T cell-mediated alloreactivity while preserving their therapeutic potential.

To study this, we first induced alloreactive T cells *in vitro* by coculturing healthy donor T cells with irradiated, mismatched PBMCs (Figure S6A). Over the 2-week culture, T cells expanded robustly and gradually upregulated CD70, alongside increased expression of activation markers such as CD25, CD69, CD137, and PD-1, consistent with an alloreactive phenotype (Figures S6B–S6E).^17^

**Figure 7:**
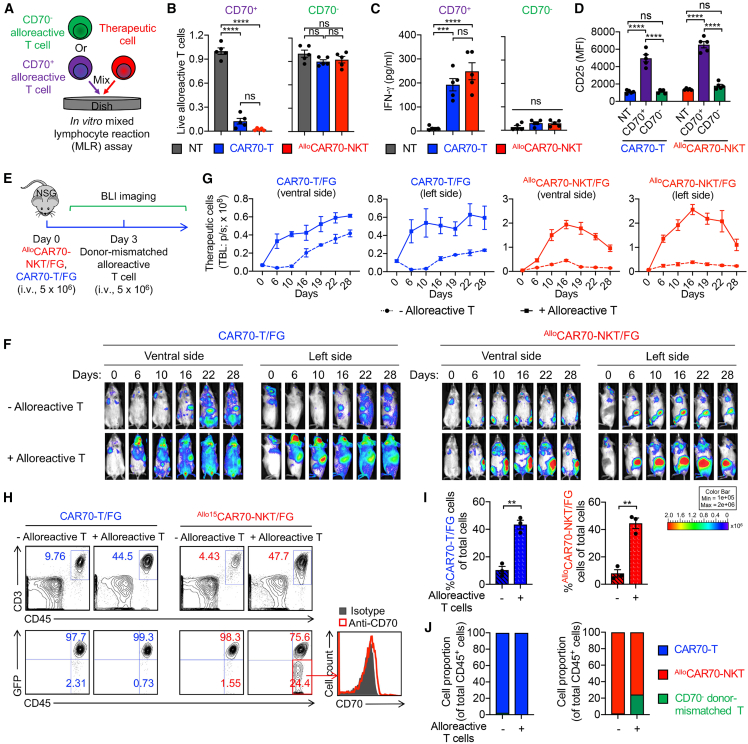
^Allo^CAR70-NKT cells selectively target alloreactive T cells via CD70-mediated recognition (A–D) Studying ^Allo^CAR70-NKT cell targeting of alloreactive T cells using an *in vitro* MLR assay. (A) Experimental design. (B) Alloreactive T cell killing data at 8 h (*n* = 5). NT, no therapeutic cell treatment. (C) ELISA analyses of IFN-γ production by the indicated therapeutic cells (*n* = 5). (D) Fluorescence-activated cell sorting (FACS) analyses of CD25 expression on the indicated therapeutic cells (*n* = 5). NT, no addition of alloreactive T cells. (E–J) Studying ^Allo^CAR70-NKT cell targeting of alloreactive T cells using a human alloreactive T cell xenograft NSG mouse model. (E) Experimental design. (F) BLI images showing the presence of therapeutic cells in experimental mice over time. Ventral and left-side views are shown. (G) Quantification of (F) (*n* = 3). (H) FACS detection of human ^Allo^CAR70-NKT/FG (identified as human CD45^+^CD3^+^GFP^+^), CAR70-T/FG (identified as human CD45^+^CD3^+^GFP^+^), and alloreactive T cells (identified as human CD45^+^CD3^+^GFP^−^) in the blood collected from experimental mice at day 20, as well as the CD70 expression on the alloreactive T cells. (I) Percentage of ^Allo^CAR70-NKT/FG and CAR70-T/FG cells among total live cells in peripheral blood (*n* = 3). (J) Cell proportion of human CD45^+^ immune cells (*n* = 3). Representative of 3 experiments. Data are presented as the mean ± SEM. ns, not significant; ∗∗*p* < 0.01, ∗∗∗*p* < 0.001, and ∗∗∗∗*p* < 0.0001 by one-way ANOVA (B–D, I, and J).

Next, we assessed the ability of ^Allo^CAR70-NKT cells to kill CD70^+^ alloreactive T cells using a mixed lymphocyte reaction (MLR) assay (Figure 7A). Both conventional CAR70-T and ^Allo^CAR70-NKT cells effectively killed CD70^+^, but not CD70^−^, alloreactive T cells within 8 h, highlighting the CAR-specific nature of the cytotoxicity (Figure 7B). Killing correlated with enhanced IFN-γ secretion and CD25 upregulation of therapeutic cells (Figures 7C and 7D).

We further validated these findings *in vivo* using a human alloreactive T cell xenograft NSG mouse model (Figure 7E). FG-labeled ^Allo^CAR70-NKT (denoted as ^Allo^CAR70-NKT/FG) or CAR70-T (denoted as CAR70-T/FG) cells were injected into mice, followed 3 days later by donor-mismatched alloreactive T cells. Bioluminescence imaging (BLI) was used to monitor the pharmacokinetics and biodistribution of the therapeutic cells (Figure 7E).

In the absence of alloreactive T cells, CAR70-T/FG cells expanded and disseminated across multiple organs, including the lungs, liver, spleen, and gastrointestinal tract (Figures 7F and 7G). Upon addition of alloreactive T cells, their expansion and persistence were significantly enhanced (Figures 7F and 7G). On the other hand, ^Allo^CAR70-NKT/FG cells initially expanded, peaked around day 16, and then gradually declined, and these cells predominantly homed to the bone marrow in the experimental mice (Figures 7F and 7G). The presence of alloreactive T cells boosted their expansion and persistence, suggesting these CD70^+^ alloreactive T cells served as a stimulatory “vaccine” to promote therapeutic cell activation and durability *in vivo* (Figures 7F and 7G). Moreover, compared to conditions without alloreactive T cells, both ^Allo^CAR70-NKT/FG and conventional CAR70-T/FG cells showed increased expression of exhaustion markers, including PD-1, TIM-3, and TOX, indicating that alloreactive T cells activate the therapeutic cells and induce exhaustion marker upregulation (Figures S6F and S6G). However, ^Allo^CAR70-NKT/FG cells exhibited significantly lower levels of these markers compared to conventional CAR70-T/FG cells (Figures S6F and S6G), suggesting that ^Allo^CAR70-NKT/FG cells retain better functionality even in the presence of alloreactive T cells.

We further analyzed the human immune cell populations in the blood of the experimental mice. Consistent with the BLI findings, both CAR70-T/FG and ^Allo^CAR70-NKT/FG cells exhibited expansion in the presence of alloreactive T cells (Figures 7H and 7I). Interestingly, in CAR70-T cell-treated mice, only GFP^+^ CAR70-T/FG cells were detected, with complete loss of alloreactive T cells, likely due to CAR-T cell-induced allorejection (Figures 7H–7J). Conversely, in ^Allo^CAR70-NKT cell-treated mice, both GFP^+ Allo^CAR70-NKT/FG cells and GFP^−^ alloreactive T cells were detected, specifically, only CD70^−^ alloreactive T cells remained (Figures 7H–7J). Taken together, both conventional CAR70-T cells and ^Allo^CAR70-NKT cells are capable of targeting CD70^+^ alloreactive T cells through CAR-mediated killing, as demonstrated by their inability to kill CD70^−^ alloreactive T cells in an 8-h short-term co-culture assay (Figures 7A–7D). However, over prolonged interaction, conventional CAR70-T cells can induce a host-versus-graft response due to their diverse TCRs recognizing mismatched major histocompatibility complex (MHC) molecules on other alloreactive T cells, leading to their elimination (Figures 7H–7J).^67^^,^^68^^,^^69^ In contrast, ^Allo^CAR70-NKT cells do not recognize mismatched MHC molecules due to their MHC-independent, CD1d-restricted TCRs and thus spare other alloreactive T cells (Figures 7H–7J).^70^^,^^71^^,^^72^

To verify this, we performed a long-term *in vitro* MLR assay by co-culturing healthy donor-derived alloreactive T cells with therapeutic cells (Figure S6H). Since CD70 is upregulated on activated alloreactive T cells, making them susceptible to CAR70-mediated killing, we used non-CAR-engineered T and ^Allo^NKT cells for this assay to specifically assess TCR-mediated effects (Figure S6H). After 4 days of co-culture, we observed that conventional T cells significantly depleted alloreactive T cells and produced high levels of IFN-γ, indicating TCR-mediated recognition and killing (Figures S6I and S6J). In contrast, ^Allo^NKT cells did not deplete alloreactive T cells and showed minimal IFN-γ production (Figures S6I and S6J). These results are consistent with the *in vivo* findings (Figures 7H–7J) and demonstrate that conventional T or CAR70-T cells can mediate TCR-driven rejection of alloreactive T cells, whereas ^Allo^NKT and ^Allo^CAR70-NKT cells do not, highlighting the immunological safety of the allogeneic CAR-NKT cell platform (Figure S6K).

In summary, ^Allo^CAR70-NKT cells leverage the presence of CD70^+^ alloreactive T cells to enhance their own expansion and persistence, while avoiding GvH response by sparing CD70^−^ host T cells. These features position ^Allo^CAR70-NKT cells as a safe and effective off-the-shelf therapeutic platform for RCC, with strong *in vivo* performance and minimized risk of host immune disruption.

### ^Allo^CAR70-NKT cells exhibit high safety with minimal risk ofCRS and tissue damage

Conventional CAR-T cell therapies are frequently associated with toxicities such as cytokine release syndrome (CRS) and immune effector cell-associated neurotoxicity syndrome.^36^^,^^73^ These adverse events are primarily driven by CAR-T cell interactions with pro-inflammatory myeloid cells in the TME or circulation, leading to excessive cytokine production and a hyperinflammatory state.^73^^,^^74^^,^^75^^,^^76^ We next evaluated the safety profile of ^Allo^CAR70-NKT cells in direct comparison with conventional CAR70-T cells.

We first assessed the potential for CRS toxicity induced by ^Allo^CAR70-NKT cells using a 786-O-FG human RCC xenograft mouse model (Figure S7A). The xenograft models have previously demonstrated a key role for host mouse macrophages in amplifying CAR-T-induced CRS responses.^65^^,^^74^ Notably, in mice bearing a high peritoneal tumor burden, treatment with ^Allo^CAR70-NKT cells resulted in significantly attenuated CRS-related toxicity compared to CAR70-T cells. This was evidenced by more stable body weight and markedly lower levels of CRS-associated biomarkers, including mouse IL-6 and serum amyloid A-3 (SAA-3), in the serum (Figures S7B and S7C). While analysis of human cytokines revealed comparable levels of effector cytokines such as IFN-γ and TNF-α between the two groups, ^Allo^CAR70-NKT cell-treated mice exhibited significantly lower levels of human IL-6 (Figure S7D). These findings suggest that ^Allo^CAR70-NKT cells may reduce the risk of CRS-like responses without compromising their antitumor effector functions. This favorable safety profile may be attributed to their innate-like NK cell characteristics and their ability to target and modulate mouse peritoneal macrophages (Figure S7E), thereby mitigating macrophage-driven CRS amplification.^65^^,^^74^

To better recapitulate the human TME with a functional human myeloid component, we employed an advanced humanized mouse model using NSG-SGM3 mice, which express human stem cell factor, granulocyte-macrophage colony-stimulating factor, and IL-3 to support the development and maintenance of human myeloid cells (Figure S7F).^73^ Flow cytometric analysis confirmed successful engraftment of human myeloid cells within the peritoneal fluid, the primary TME in this model (Figures S7G and S7H). These cells expressed high levels of M2-like macrophage markers, including CD163 and CD206, consistent with an immunosuppressive, tumor-supportive phenotype (Figure S7I).^77^^,^^78^ Interestingly, treatment with conventional CAR70-T cells resulted in a significant expansion of these immunosuppressive myeloid cells in the peritoneal fluid, exacerbating cell-mediated toxicity (Figures S7G and S7H). This was reflected by a notable reduction in body weight and elevated serum levels of both human IL-6 and mouse SAA-3 (Figures S7J and S7K). In contrast, treatment with ^Allo^CAR70-NKT cells led to a marked depletion of human myeloid cells (Figures S7G and S7H). This effect is likely mediated by the NKT TCR through CD1d recognition, as the myeloid cells expressed high levels of human CD1d (Figure S7I). Mice treated with ^Allo^CAR70-NKT cells maintained stable body weight and exhibited significantly lower levels of CRS-associated biomarkers, suggesting a reduced risk of systemic toxicity (Figures S7J and S7K). These results support the ability of ^Allo^CAR70-NKT cells to selectively deplete CD1d^+^ tumor-associated myeloid cells within the TME, thereby mitigating CRS while preserving therapeutic efficacy.

Lastly, we evaluated the long-term safety profile of ^Allo^CAR70-NKT cells, with a focus on potential organ toxicity. In contrast to conventional CAR70-T cells, which induced significant elevations of organ damage markers in serum, including urea, alanine aminotransferase (ALT), aspartate aminotransferase (AST), bilirubin, and glutamate dehydrogenase (GLDH), ^Allo^CAR70-NKT cells did not elicit such toxic effects (Figures S7L and S7M). The observed toxicity in the CAR70-T cell-treated group is likely attributable to severe xenogeneic GvHD triggered by their polymorphic TCRs in the xenograft mouse model.^21^^,^^65^ Notably, ^Allo^CAR70-NKT cells demonstrated sustained safety, with minimal signs of organ damage even 120 days after adoptive transfer into NSG mice (Figure S7N). These findings collectively highlight the favorable safety profile of ^Allo^CAR70-NKT cells and support their potential as a durable and safe off-the-shelf cellular immunotherapy.

## Discussion

CD70 represents a promising tumor-associated antigen due to its consistent overexpression across RCC tumors (Figures 1C–1F). The therapeutic landscape for CD70-targeted therapies in RCC is rapidly evolving with multiple strategies under investigation such as monoclonal antibodies, antibody-drug conjugates, CAR-T cells, and CAR-NK cells. Among them, two allogeneic CAR-T cell platforms, CTX130 and ALLO-316, are currently in clinical development. The phase I trial of CTX130 reported a disease control rate of 77%, with one complete response.^9^^,^^79^^,^^80^ Similarly, another trial evaluating the safety and efficacy of ALLO-316 showed a favorable safety profile and an 89% disease control rate.^79^^,^^80^ These clinical results are promising and serve as a proof of concept demonstrating the effectiveness and safety of CD70-directed CAR cell therapy. Building on this progress, we have developed an ^Allo^CAR70-NKT cell therapy as a next-generation, off-the-shelf immunotherapy for mRCC. This platform combines tumor-directed cytotoxicity with TME-modulating activity and includes the added benefit of eliminating host alloreactive T cells, offering a multifaceted strategy for effective and safe treatment of advanced RCC.

Interestingly, ^Allo^CAR70-NKT cells naturally lack CD70 expression, thereby eliminating the need for *CD70* gene knockout and mitigating the risk of fratricide and activation-induced exhaustion (Figures 2F–2J).^41^ This phenotype appears to be a consistent feature of our *in vitro* differentiation protocol and the intrinsic properties of HSPC-derived cell products, independent of the specific CAR construct used (e.g., CAR70 or BCMA-directed CAR) (Figures 2H and 2I). Further studies are warranted to elucidate the underlying mechanisms governing the absence of CD70 expression in these HSPC-engineered NKT cells.

^Allo^CAR70-NKT cells exhibited robust cytotoxic activity and multimodal tumor-targeting across a diverse panel of established RCC cell lines and primary patient-derived RCC tumor cells with heterogeneous CD70 expression, both *in vitro* and *in vivo* (Figures 3, 4, 5, and S3). Notably, this included effective targeting of tumors with low to moderate CD70 levels, which are typically resistant to conventional CAR70-T cell therapies (Figures 3 and S3). These findings highlight the capacity of ^Allo^CAR70-NKT cells to mediate CAR-independent cytotoxicity through their endogenous NKRs, thereby mitigating the risk of antigen escape and promoting sustained antitumor responses in the context of CD70 downregulation.^11^

The immunosuppressive TME, enriched with TAMs and MDSCs, contributes significantly to RCC progression and resistance to therapy.^23^ Current clinical strategies for RCC commonly involve combining anti-angiogenic agents, such as VEGF or VEGF receptor blocking agents or tyrosine kinase inhibitors, with immune checkpoint blockade, which has demonstrated clinical benefit.^23^^,^^81^^,^^82^ In this study, we demonstrated that ^Allo^CAR70-NKT cells effectively eliminated immunosuppressive TAMs and MDSCs derived from primary RCC tumors via NKT TCR-mediated cytotoxicity (Figure 6). Moreover, ^Allo^CAR70-NKT cells exhibited enhanced resistance to TME-mediated inhibition and superior TAM depletion *in vivo* compared to conventional CAR70-T cells (Figures 6I–6Q). These results underscore the multiple functionalities of ^Allo^CAR70-NKT cells in exerting direct antitumor activity and remodeling the immunosuppressive RCC TME.

Significantly, allogeneic CAR70-NKT cells exhibited selective cytotoxicity toward CD70^+^ alloreactive T cells. They notably expanded upon activation of their CD70-directed alloreactivity, supporting their enhanced *in vivo* persistence and tumor-killing capacity (Figure 7). Importantly, unlike conventional CAR70-T cells, which indiscriminately deplete both CD70^+^ and CD70^−^ alloreactive T cells and pose a high risk of GvH reactions, ^Allo^CAR70-NKT cells spared CD70^−^ alloreactive T cells, indicating a superior safety profile (Figures 7E–7J). These characteristics align with the intrinsic properties of HSPC-engineered CAR-NKT cells, as well as PBMC-derived unedited NKT cells, which are naturally resistant to host T cell-mediated allorejection and exhibit minimal risk of GvHD.^21^^,^^65^^,^^83^

CD70 is upregulated as an activation-induced marker on T cells, B cells, and dendritic cells during immune responses. Therefore, prolonged depletion of CD70-expressing immune populations could potentially impair adaptive immune functions, including memory T cell formation, antigen recall responses, and overall responsiveness to infections or vaccines.^84^ However, given the off-the-shelf nature of ^Allo^CAR70-NKT cell therapy, these cells are not expected to persist long-term in patients. Unlike autologous CAR70-T cells, ^Allo^CAR70-NKT cells are eventually eliminated by the host immune system following the therapeutic window, a phenomenon commonly observed across all allogeneic cell therapies.^13^^,^^64^^,^^85^^,^^86^^,^^87^ As a result, the depletion of CD70-expressing immune cells by ^Allo^CAR70-NKT cells is expected to be temporary, which offers a safety advantage over autologous CAR70-T cell therapies of lower risk of long-term immunosuppression.

### Limitations of the study

In our study, we performed a side-by-side comparison between ^Allo^CAR70-NKT and conventional CAR70-T cells lacking IL-15 engineering, as this reflects the current standard among Food and Drug Administration -approved CAR-T therapies.^39^^,^^88^ However, we acknowledge that including IL-15-engineered CAR-T cells in future studies would provide a more balanced and comprehensive comparison. Notably, recent clinical trials have shown that IL-15-engineered conventional CAR-T cells can enhance antitumor efficacy in solid tumor patients but are also associated with increased risk of severe CRS.^89^ In contrast, autologous IL-15-engineered CAR-NKT cells have demonstrated a favorable safety profile.^32^ These findings suggest that IL-15 engineering may be better tolerated in the CAR-NKT cell context.^90^ Beyond oncology, CD70-targeting ^Allo^CAR70-NKT cells may have utility in T cell-mediated autoimmune diseases, where activated CD70^+^ T cells contribute to pathogenesis, including multiple sclerosis, inflammatory bowel disease, and lupus.^91^^,^^92^^,^^93^ Thus, the ability to selectively eliminate activated T cells positions ^Allo^CAR70-NKT cells as a promising platform for both cancer and autoimmune indications, meriting further preclinical exploration.

## Resource availability

### Lead contact

Further information and requests for resources and reagents should be directed to and will be fulfilled by the lead contact, Lili Yang (liliyang@ucla.edu).

### Materials availability

This study did not generate new unique materials.

### Data and code availability

• Data: This study analyzed publicly available datasets at The Human Protein Atlas and the Genomic Data Commons. The details are listed in the key resources table. Other data reported in the paper will be shared upon request from the lead contact.
• Code: This paper does not report original code.
• Any additional information required to reanalyze the data reported in this paper is available from the lead contact upon request.

## Acknowledgments

We thank the UCLA animal facility for providing animal support, the UCLA Translational Pathology Core Laboratory (TPCL) for providing histology support, and the UCLA CFAR Virology Core for providing human cells. This work was supported by a Partnering Opportunity for Discovery Stage Research Projects award and Partnering Opportunity for Translational Research Projects awards from the 10.13039/100000900California Institute for Regenerative Medicine (DISC2-11157, DISC2-13015, TRAN1-12250, and TRAN1-16050 to L.Y.), a Department of Defense Kidney Cancer Research Program award (KC230215 to A.I.C., L.W., and L.Y.), and a UCLA BSCRC Innovation Grant. L.Y. is a member of UCLA Parker Institute for Cancer Immunotherapy (PICI). Y.-R.L. is a postdoctoral fellow supported by a UCLA Chancellor’s Award for Postdoctoral Research and a UCLA Goodman-Luskin Microbiome Center Collaborative Research Fellowship award. A.I.C. is supported by the Perkins Foundation and Nancy and Donald DeBrier. We would like to acknowledge Moe Ishihara and Yiming Jin who assisted on this project. Some figures were created with BioRender (biorender.com).

## Author contributions

Y.-R.L. and J. Hu designed the experiments, analyzed the data, and wrote the manuscript. L.Y. conceived and oversaw the study, with advice from T. Hsiai, A.I.C., and L.W. Y.-R.L. performed all experiments, with assistance from J. Hu, Z. Li, E.Z., Y.C., T. Halladay, X.S., Y.F., Y.Z., Z. Lyu, Y.T., J. Huang, A.S.Z., N.Y.M., C.Z., and Y.X. H.Z. and A.I.C. provided the primary patient samples.

## Declaration of interests

L.Y. is a scientific advisor to AlzChem and Amberstone Biosciences and a co-founder, stockholder, and advisory board member of Appia Bio. None of the declared companies contributed to or directed any of the research reported in this article.

## STAR★Methods

### Key resources table

**Table:** 

REAGENT or RESOURCE	SOURCE	IDENTIFIER
**Antibodies**		

Anti-human TCR αβ (Clone IP26)	BioLegend	CAT#306716, RRID: AB_1953257
Anti-human CD1d (Clone 51.1)	BioLegend	CAT#350308, RRID: AB_10642829
Anti-human CD3 (clone HIT3a)	BioLegend	CAT#300329, RRID: AB_10552893
Anti-human CD4 (Clone OKT4)	BioLegend	CAT#317414, RRID: AB_571959
Anti-human CD8 (Clone SK1)	BioLegend	CAT#344714, RRID: AB_2044006
Anti-human CD14 (Clone HCD14)	BioLegend	CAT#367122, RRID: AB_2687385
Anti-human CD15 (Clone W6D3)	BioLegend	CAT#323008, RRID: AB_756014
Anti-human CD19 (Clone HIB19)	BioLegend	CAT#302212, RRID: AB_314242
Anti-human CD20 (Clone 2H7)	BioLegend	CAT#302306, RRID: AB_2924581
Anti-human CD27 (Clone M-T271)	BioLegend	CAT#986904, RRID: AB_3068050
Anti-human CD31 (Clone WM59)	BioLegend	CAT#303103, RRID: AB_314329
Anti-human CD34 (Clone 581)	BioLegend	CAT#343520, RRID: AB_1937269
Anti-human CD45 (Clone HI30)	BioLegend	CAT#982318, RRID: AB_2888786
Anti-human CD56 (Clone HCD56)	BioLegend	CAT#318304, RRID: AB_604100
Anti-human CD69 (Clone FN50)	BioLegend	CAT#310912, RRID: AB_314847
Anti-human CD70 (Clone WM53)	BioLegend	CAT#355109, RRID: AB_2562480
Purified anti-human CD70 (Clone 113-16)	BioLegend	CAT#355102, RRID: AB_2561429
Anti-human CD107a (Clone H4A3)	BioLegend	CAT#328606, RRID: AB_1186036
Anti-human CD112 (Clone TX31)	BioLegend	CAT#337410, RRID: AB_2269088
Anti-human CD155 (Clone SKII.4)	BioLegend	CAT#337614, RRID: AB_2565747
Anti-human CD11b (Clone ICRF44)	BioLegend	CAT#982614, RRID: AB_2924634
Anti-human CD163 (Clone GHI/61)	BioLegend	CAT#333610, RRID: AB_2074533
Anti-human CD206 (Clone 15-2)	BioLegend	CAT#321120, RRID: AB_2144930
Anti-human MICA/B (Clone 6D4)	BioLegend	CAT#320908, RRID: AB_493195
Anti-human 41BBL (Clone 5F4)	BioLegend	CAT#311504, RRID: AB_314883
Anti-human CD83 (Clone HB15e)	BioLegend	CAT#305330, RRID: AB_2566393
Anti-human CD86 (Clone IT2.2)	BioLegend	CAT#305412, RRID: AB_493231
Anti-human CCR2 (Clone K036C2)	BioLegend	CAT#357208, RRID: AB_2562239
Anti-human CCR5 (Clone J418F1)	BioLegend	CAT#359120, RRID: AB_2564071
Anti-human CCR6 (Clone G034E3)	BioLegend	CAT#353410, RRID: AB_10913815
Anti-human CCR7 (Clone G043H7)	BioLegend	CAT#353212, RRID: AB_10916390
Anti-human CXCR3 (Clone G025H7)	BioLegend	CAT#353719, RRID: AB_11218804
Anti-human CXCR4 (Clone 12G5)	BioLegend	CAT#306510, RRID: AB_314616
Anti-human PD-1 (Clone A17188A)	BioLegend	CAT#379209, RRID: AB_2922607
Anti-human TIM-3 (Clone A18087E)	BioLegend	CAT#364803, RRID: AB_2910409
Anti-human LAG-3 (Clone 7H2C65)	BioLegend	CAT#369208, RRID: AB_2629835
Anti-human NKG2D (Clone 1D11)	BioLegend	CAT#320812, RRID: AB_2234394
Anti-human DNAM-1 (Clone 11A8)	BioLegend	CAT#338312, RRID: AB_2561952
Anti-human NKp30 (Clone P30-15)	BioLegend	CAT#325210, RRID: AB_2149449
Anti-human NKp46 (Clone 9E2)	BioLegend	CAT#137606, RRID: AB_2298210
Anti-human IFN-γ (Clone B27)	BioLegend	CAT#506518, RRID: AB_2123321
Anti-human Granzyme B (Clone QA16A02)	BioLegend	CAT#372204, RRID: AB_2687028
Anti-human Perforin (Clone dG9)	BioLegend	CAT#308126, RRID: AB_2572049
Anti-human TNF-α (Clone MAb11)	BioLegend	CAT#502912, RRID: AB_315264
Anti-human IL-2 (Clone MQ1-17H12)	BioLegend	CAT#500342, RRID: AB_2562854
Anti-human β2-microglobulin (B2M) (Clone 2M2)	BioLegend	CAT#316312, RRID: AB_10641281
Anti-human HLA-DR (Clone L243)	BioLegend	CAT#307618, RRID: AB_493586
Anti-human HLA-DR, DP, DQ (Clone Tü39)	BioLegend	CAT#361706, RRID: AB_2563192
Anti-mouse CD11b (Clone M1/70)	BioLegend	CAT#101206, RRID: AB_312789
Anti-mouse F4/80 (Clone BM8)	BioLegend	CAT#123126, RRID: AB_893495
Anti-mouse CD1d (Clone 1B1)	BioLegend	CAT#123522, RRID: AB_2715920 CAT#123524, RRID: AB_2721672
Purified mouse lgG2b, κ isotype control antibody (Clone MG2b-57)	BioLegend	CAT#401202, RRID: AB_2744505
Anti-human TCR Vα24-Jβ18 (Clone 6B11)	BD Biosciences	CAT#552825, RRID: AB_394478
Anti-human fibroblast activation protein alpha (FAP) (Clone 427819)	R&D Systems	CAT# MAB3715-SP
Anti-human ULBP-1 (Clone 170818)	R&D Systems	CAT#FAB1380P, RRID: AB_2687471
Anti-human ULBP-2,5,6 (Clone 165903)	R&D Systems	CAT#FAB1298A, RRID: AB_2257142
Anti-human TOX (Clone TXRX10)	Thermo Fisher	CAT#12-6502-82, RRID: AB_10855034
Goat anti-Mouse IgG F(ab’)2 Secondary Antibody, Biotin	Thermo Fisher	CAT#31803, RRID: AB_228311
Mouse Fc Block (anti-mouse CD16/32)	BD Biosciences	CAT#553142, RRID: AB_394657
Human Fc Receptor Blocking Solution (TruStain FcX)	BioLegend	CAT#422302, RRID: AB_2818986
Anti-human IFN-γ (ELISA, capture; Clone NIB42)	BD Biosciences	CAT#551221, RRID: AB_394099
Anti-human IFN-γ (ELISA, detection; Clone 4S.B3)	BD Biosciences	CAT#554550, RRID: AB_395472
Anti-human TNF-α (ELISA, capture; Clone MAb1)	BD Biosciences	CAT#551220, RRID: AB_394098
Anti-human TNF-α (ELISA, detection; Clone MAb11)	BD Biosciences	CAT#554511, RRID: AB_395442
Anti-human IL-2 (ELISA, capture; Clone MQ1-17H12)	BD Biosciences	CAT#554563; RRID: AB_398570
Anti-human IL-2 (ELISA, detection; Clone B33-2)	BD Biosciences	CAT#555040; RRID: AB_395666
Anti-human IL-4 (ELISA, capture; Clone 8D4-8)	BD Biosciences	CAT#554515; RRID: AB_398567
Anti-human IL-4 (ELISA, detection; Clone MP4-25D2)	BD Biosciences	CAT#554483; RRID: AB_395422
Anti-human IL-15 (ELISA, capture; Clone G243-935)	BD Biosciences	CAT#554712; RRID: AB_2561319
Anti-human IL-15 (ELISA, detection; Clone G243-886)	BD Biosciences	CAT#554713; RRID: AB_2561320
Anti-mouse IL-6 (ELISA, capture; Clone MP5-20F3)	BD Biosciences	CAT#554400; RRID: AB_315336
Anti-mouse IL-6 (ELISA, detection; Clone MP5-32C11)	BD Biosciences	CAT#554402; RRID: AB_2127458

**Bacterial and virus strains**		

Lenti/iNKT	This paper	N/A
Lenti/iNKT-CAR70-IL-15	This paper	N/A
Lenti/iNKT-BCAR-IL-15	This paper	N/A
Lenti/CAR70	This paper	N/A
Lenti/CAR70-EGFP	This paper	N/A
Lenti/BCAR	This paper	N/A
Lenti/CD70	This paper	N/A
Lenti/FG	This paper	N/A

**Biological samples**		

Primary RCC patient tumor samples	UCLA	N/A
Human peripheral blood mononuclear cells (PBMCs)	UCLA	N/A
Cord blood CD34^+^ stem/progenitor cells	HemaCare	N/A
RCC patient-derived xenografts (PDX)	This paper	N/A

**Chemicals, peptides, and recombinant proteins**		

Streptavidin-HRP conjugate	Invitrogen	CAT#SA10001
Human IFN-γ (ELISA, standard)	eBioscience	CAT#29-8319-65
Human TNF-α (ELISA, standard)	eBioscience	CAT#29-8329-65
Human IL-2 (ELISA, standard)	eBioscience	CAT#29-8029-65
Human IL-4 (ELISA, standard)	eBioscience	CAT#39-8049-65
Human IL-6 (ELISA, standard)	eBioscience	CAT#29-8069-65
Human IL-15 (ELISA, standard)	eBioscience	CAT#29-8159-60
Human IL-17A (ELISA, standard)	eBioscience	CAT#29-8179-65
Mouse IL-6 (ELISA, standard)	eBioscience	CAT#29-8061-65
Tetramethylbenzidine (TMB)	KPL	CAT#5120-0053
α-Galactosylceramide (KRN7000)	Avanti Polar Lipids	SKU#867000P-1mg
Recombinant human IL-2	Peprotech	CAT#200-02
Recombinant human IL-3	Peprotech	CAT#200-03
Recombinant human IL-4	Peprotech	CAT#200-04
Recombinant human IL-7	Peprotech	CAT#200-07
Recombinant human IL-13	Peprotech	CAT#200-13
Recombinant human IL-15	Peprotech	CAT#200-15
Recombinant human IL-21	Peprotech	CAT#200-21
Recombinant human IFN-γ	Peprotech	CAT#300-02
Recombinant human Flt3-Ligand	Peprotech	CAT#300-19
Recombinant human SCF	Peprotech	CAT#300-07
Recombinant human TPO	Peprotech	CAT#300-18
Recombinant human GM-CSF	Peprotech	CAT#300-03
Recombinant human M-CSF	Peprotech	CAT#300-25
L-ascorbic acid 2-phosphate	Sigma	CAT#A8960-5G
B27™ Supplement (50X), serum free	ThermoFisher	CAT#17504044
Cas9-NLS purified protein	UC Berkeley	N/A
X-VIVO 15 Serum-free Hematopoietic Cell Medium	Lonza	CAT#04-418Q
RPMI1640 cell culture medium	Corning Cellgro	CAT#10-040-CV
DMEM cell culture medium	Corning Cellgro	CAT#10-013-CV
Fetal Bovine Serum (FBS)	Sigma	CAT#F2442
MACS BSA stock solution	Miltenyi	CAT#130-091-376
30% BSA	Gemini	CAT#700-110-100
Penicillin-Streptomycine-Glutamine (P/S/G)	Gibco	CAT#10378016
Penicillin: streptomycin (pen:strep) solution (P/S)	Gemini Bio-products	CAT#400-109-100
MEM non-essential amino acids (NEAA)	Gibco	CAT#11140050
HEPES Buffer Solution	Gibco	CAT#15630080
Sodium Pyruvate	Gibco	CAT#11360070
Beta-Mercaptoethanol	Sigma	SKU#M6250
Normocin	Invivogen	CAT#ant-nr-2
Fixable Viability Dye eFluor506	eBioscience	CAT#65-0866-14
Cell Fixation/Permeabilization Kit	BD Biosciences	CAT#554714
RetroNectin recombination human fibronectin fragment, 2.5mg	Takara	CAT#T100B
10% neutral-buffered formalin	Richard-Allan Scientific	CAT#5705
D-Luciferin Potassium Salt Bioluminescent Substrate	Revivi	CAT#122799-100MG
Fluriso (Isoflurane)	Vet One	CAT#502017
Phosphate Buffered Saline (PBS) pH 7.4 (1X)	Gibco	CAT#10010-023
Formaldehyde	Sigma-Aldrich	CAT#F8775
Golgistop Protein Transport Inhibitor	BD Biosciences	CAT#554724
Poloxamer Synperonic F108	Millipore Sigma	CAT#07579-250G-F
Prostaglandin E2 (PGE2)	Cayman Chemical	CAT#14010
TrypLE™ Express Enzyme	ThermoFisher Scientific	CAT#12605010
Phorbol-12-myristate-13-acetate (PMA)	Sigma	CAT#524400
Ionomycin, Calcium salt, *Streptomyces conglobatus*	Sigma	CAT#407952
ImmunoCult™ Human CD3/CD28/CD2 T cell Activator	Stem Cell Technologies	CAT#10970
MethoCult™H4330 MethycelluloseBased Medium	Stem Cell Technologies	CAT#04330
CTS™ OpTmizer™ T cell Expansion SFM (no phenol red, bottle format, cat.	Thermo Fisher Scientific	CAT#A3705001
CryoStor ® Cell Cryopreservation Media CS10	MilliporeSigma	CAT#C2874
Iscove’s Modified Dulbecco’s Medium	MilliporeSigma	CAT#I3390

**Critical commercial assays**		

Human NK Cell Isolation Kit	Miltenyi Biotec	CAT#130-092-657
Human CD34 MicroBeads Kit	Miltenyi Biotec	CAT#130-046-703
Human CD14 MicroBeads Kit	Miltenyi Biotec	CAT#130-050-201
Human Anti-NKT MicroBeads	Miltenyi Biotec	CAT#130-094-842
Human Anti-HLA-DR MicroBeads	Miltenyi Biotec	CAT#130-046-101
Human tumor cell isolation kit	Miltenyi Biotec	CAT#130-108-339
Fixation/Permeabilization Solution Kit	BD Sciences	CAT#55474
Amaxa™ P3 Primary Cell 4D-Nucleofector™ X Kit S	Lonza	CAT#V4XP-3032
Dynabeads Human T-Activator CD3/CD28	ThermoFisher	CAT#11131D
miRNeasy Mini Kit	Qiagen	CAT#217004
Chromium single cell V(D)J enrichment kit, human T cell	10 x Genomics	CAT#1000005
Cryostor cell cryopreservation media	Sigma	CAT#C2874-100ML
StemSpan“” Lymphoid Differentiation Coating Material (100X)	Stem Cell Technologies	CAT#9925
StemSpan" SFEM II	Stem Cell Technologies	CAT#9605
ImmunoCult" Human CD3/CD28/CD2 T cell Activator	Stem Cell Technologies	CAT#10970
TransIT-Lenti Transfection Reagent	Mirus Bio	CAT#MIR 6600
Amicon Ultra-15 Centrifugal Filter Unit	Sigma	CAT#UFC910024
Nunclon Sphera-Treated 96-Well Microplate	ThermoFisher	CAT#174929
CellQART 6-Well Cell Culture Insert	Millicell	CAT#9301002
Human IL-17A ELISA MAX Deluxe Kit	BioLegend	CAT#433915
CUBIC-L reagent	TCI	Product#T3740
CUBIC-R+(M) reagent	TCI	Product#T3741
CUBIC mounting solution (RI 1.520)	TCI	Product#M3294
Mouse SAA-3 ELISA Kit	Millipore Sigma	Product#EZMSAA3
Urea Nitrogen (BUN) Colorimetric Detection Kit	ThermoFisher Scientific	CAT#EIABUN
Mouse AST ELISA Kit	Abcam	CAT#ab263882
Mouse ALT ELISA Kit	Abcam	CAT#ab282882
Mouse Bilirubin ELISA Kit	MyBioSource	CAT#MBS3805359
Mouse Glutamate dehydrogenase (GLDH) ELISA Kit	MyBioSource	CAT#MBS761948

**Deposited data**		

Gene mRNA expression levels in RCC tumor cells versus normal kidney tissues	The Human Protein Atlas	https://www.proteinatlas.org/about/download
CD70 mRNA expression in various human cancer types	Genomic Data Commons Data Portal	https://portal.gdc.cancer.gov

**Experimental models: Cell lines**		

Human renal cell carcinoma cell line 786-O	ATCC	CRL-1932
Human renal cell carcinoma cell line ACHN	ATCC	CRL-1611
Human multiple myeloma cell line MM.1S	ATCC	CRL-2974
Human chronic myelogenous leukemia cell line K562	ATCC	CRL-2974
Human artificial APC cell line	This paper	N/A
Human multiple myeloma cell line MM.1S-FG	This paper	N/A
Human multiple myeloma cell line MM.1S-CD70-FG	This paper	N/A
Human renal cell carcinoma cell line 786-*O*-FG	This paper	N/A
Human renal cell carcinoma cell line 786-*O*-FG^CD70−/−^	This paper	N/A
Human renal cell carcinoma cell line ACHN-FG	This paper	N/A

**Experimental models: Organisms/strains**		

NOD.Cg-*Prkdc*^*scid*^*Il2rg*^*tm1Wjl*^/SzJ (NSG)	The Jackson Laboratory	Strain #: 005557
NOD.Cg-*Prkdc*^*scid*^*Il2rg*^*tm1Wjl*^ Tg(CMV-IL3,CSF2,KITLG)1Eav/MloySzJ (NSG-SGM3)	The Jackson Laboratory	Strain #: 013062

**Oligonucleotides**		

gRNA (*CD70*): GCCAACGGCTCGACCCCTGT	Synthego	N/A

**Recombinant DNA**		

Vector: parental lentivector pMNDW	Giannoni et al.^94^ Zhu et al.^35^	N/A

**Software and algorithms**		

FlowJo Software	FlowJo	https://www.flowjo.com/solutions/flowjo/downloads
AURA imaging software	Spectral Instruments Imaging	https://spectralinvivo.com/software/
ImageJ	ImageJ	https://imagej.net/Downloads
Amira-Avizo 3D	ThermoFisher	https://www.thermofisher.com/software-em-3d-vis/customerportal/download-center/amira-avizo-3d-installers/
Prism 9	Graphpad	https://www.graphpad.com/scientific-software/prism/
MATLAB	The MathWorks, Inc	http://www.mathworks.com/products/matlab.html
R	R	http://www.R-project.org/
RStudio	RStudio	https://posit.co

### Experimental model and study participant details

#### Mice

Animal studies were conducted under protocols approved by the UCLA Division of Laboratory Animal Medicine. NOD.Cg-*Prkdc*^*scid*^ *Il2rg*^*tm1Wjl*^/SzJ (NOD/SCID/IL-2Rγ^−/−^, NSG) and NOD.Cg-*Prkdc*^*scid*^ *Il2rg*^*tm1Wjl*^ Tg(CMV-IL3,CSF2,KITLG)1Eav/MloySzJ (NSG-SGM3) mice were purchased from The Jackson Laboratory, and maintained in the animal facilities of UCLA under the following housing conditions: temperature ranging from 68°F to 79°F, humidity maintained at 30%–70%, a light cycle of On at 6:00 a.m. and Off at 6:00 p.m., and room pressure set to negative. 6–10 weeks old male or female mice were used for all experiments unless otherwise indicated. Sex was not considered in the study design and analysis, as no significant differences were observed in the human RCC NSG or NSG-SGM3 mouse models used. All mice were bred and maintained under specific pathogen-free conditions, and all experiments were conducted in accordance with the animal care and use regulations of the Division of Laboratory Animal Medicine at the UCLA. Experimental mice were randomly assigned to treatment groups to avoid statistically significant differences in the baseline tumor burden.

#### Stable cell lines

Human renal cell carcinoma cell lines 786-O (cat. no. CRL-1932) and ACHN (cat. no. CRL-1611), multiple myeloma cell line MM.1S (cat. no. CRL-2974), and human embryonic kidney (HEK) 293T cell line (cat no. CRL-11268) were purchased from the American Type Culture Collection (ATCC). To establish stable tumor cell lines that overexpress both firefly luciferase and enhanced green fluorescent protein dual reporters (FG), the parental tumor cell lines were transduced with lentiviral vectors carrying the specific genes of interest (i.e., Lenti/FG). 72h after lentiviral transduction, the cells underwent flow cytometry sorting to isolate the genetically modified cells (as identified as GFP^+^ cells) necessary for creating stable cell lines. The 786-*O*-FG^CD70−/−^ cell line was generated by knocking out the CD70 gene from the parental 786-*O*-FG cell line using CRISPR/Cas9-gRNA (*CD70* gRNA sequence: AGCGCUGGAUGCACACCACG). The artificial antigen-presenting cell line (aAPC) was generated by engineering the K562 human chronic myelogenous leukemia cell line (ATCC, cat. no. CCL-243) to overexpress human CD80/CD83/CD86/41BBL co-stimulatory receptors. The aAPC-CD70 cell lines were generated by further engineering the parental aAPC line to overexpress human CD70. All cell lines used in this study were authenticated by short tandem repeat (STR) DNA profiling and regularly tested for mycoplasma contamination. Only mycoplasma-negative, authenticated cell lines were used in experiments.

#### Human participants

All experiments involving primary RCC patient samples were approved by the Ronald Reagan UCLA Medical Center. All participants provided written informed consent under protocols approved by the UCLA Institutional Review Board (IRB#24–001320). A total of 22 primary RCC patient tumor samples were collected, and the patient information is listed in Table S1. RCC patient tumor samples were mechanically dissociated into small fragments, then further processed by mashing and filtration through a 70 μm cell strainer (VWR, cat. no. 732–2758) to obtain a single-cell suspension. The suspension was subsequently centrifuged to pellet the cells. Red blood cell (RBC) lysis was performed using BioLegend’s RBC lysis protocol (BioLegend, San Diego, CA, USA, CAT #420302). Following RBC lysis, cells either proceeded to be analyzed fresh or were resuspended in a cryopreservation buffer and stored in liquid nitrogen for later experimental use. Among the 22 patient samples, 15 were used for phenotypic profiling, while 7 were utilized for the establishment of patient-derived tumor cell lines. Due to the limited sample size and the study’s primary focus on tumor-intrinsic features and immune interactions, the influence of sex or gender on experimental outcomes was not specifically assessed. We acknowledge this as a limitation and note that future studies with larger, stratified cohorts are warranted to evaluate potential sex- or gender-associated differences in tumor biology and therapeutic response. Sample allocation was determined based on experimental availability, randomization when applicable, and the suitability of each sample for downstream analyses. All samples were processed and analyzed in a blinded and de-identified manner to ensure experimental integrity.

Purified human CD34^+^ HSPCs derived from cord blood (CB) were purchased from HemaCare. Healthy donor PBMCs were obtained from the UCLA/CFAR Virology Core Laboratory under written informed consent and in compliance with federal and state regulations; no identifying information was provided. Upon receipt, both HSPCs and PBMCs were promptly aliquoted and cryopreserved in liquid nitrogen for subsequent experimental use. Donor sex and gender were not considered in the study design, which we acknowledge as a limitation. Samples were randomly allocated to experimental groups based on availability and matched across replicates to minimize batch effects.

#### Primary RCC patient-derived tumor cell lines

These studies were approved by the Ronald Reagan UCLA Medical Center under an IRB approved protocol (IRB#24–001320). A total of 7 RCC patient-derived tumor cell lines were established using the method as previously described.^58^ The patient information is listed in Table S1, under IDs 16 to 22. For some assays, the RCC tumor cell lines were further engineered with Lenti/FG to overexpress FG to enable fluorescence- and luminescence-based analyses.

### Method details

#### Media and reagents

The X-VIVO 15 Serum-Free Hematopoietic Cell Medium (cat. no. 04418Q) was purchased from Lonza. The StemSpan T cell Generation Kit (cat. no. 09940), comprising the StemSpan TM SFEM II Medium (cat. no. 09605), the StemSpan TM Lymphoid Progenitor Expansion Supplement (cat. no. 09915), the StemSpan TM LPMS (cat. no. 09930), the StemSpan TM Lymphoid Progenitor Differentiation Coating Material (cat. no. 09925), and the ImmunoCult Human CD3/CD28/CD2 T cell Activator (cat. no.10970), and MethoCultH4330 MethycelluloseBased Medium (cat. no. 04330) were purchased from StemCell Technologies. The CTS OpTmizer T cell Expansion SFM (no phenol red, bottle format, cat. no. A3705001), the RPMI 1640 cell culture medium (cat. no. MT10040CV), and the DMEM cell culture medium (cat. no. MT10013CV) were purchased from Thermo Fisher Scientific. The CryoStor Cell Cryopreservation Media CS10 (cat. no. C2874) and Iscove’s Modified Dulbecco’s Medium (cat. no. I3390) was purchased from MilliporeSigma. The homemade C10 medium was made of RPMI 1640 cell culture medium, supplemented with FBS (10% vol/vol), P/S/G (1% vol/vol), MEM NEAA (1% vol/vol), HEPES (10 mM), Sodium Pyruvate (1 mM), Beta-Mercaptoethanol (β-ME) (50 μM), and Normocin (100 μg/mL). C10 medium was used to culture human T and NKT cells. The homemade R10 medium was made of RPMI supplemented with FBS (10% vol/vol), P/S/G (1% vol/vol), and Normocin (100 μg/mL). R10 medium was used to culture tumor cells. The homemade D10 medium was made of DMEM supplemented with FBS (10% vol/vol), P/S/G (1% vol/vol), and Normocin (100μm g/ml). D10 medium was used to culture HEK 293T cells.

α-Galactosyl ceramide (αGC, KRN7000, cat. no. 867000) was purchased from Avanti Polar Lipids. Recombinant human IL-2 (cat. no. 200-02), IL-3 (cat. no. 200-03), IL-7 (cat. no. 200-07), IL-15 (cat. no. 200-15), IL-21 (cat. no. 200-21), IFN-γ (cat. no. 300-02), Flt3 ligand (Flt3L, cat. no. 300-19), macrophage colony stimulating factor (M-CSF, cat. no. 300-25), stem cell factor (SCF, cat. no. 300-07), and thrombopoietin (TPO, cat. no. 300-18) were purchased from Peprotech. Fetal Bovine Serum (FBS, lot no. 2087050) were purchased from Gibco and β-ME (cat. no. 1610710) were purchased from Bio-Rad. Penicillin Streptomycin-Glutamine (P/S/G, cat. no. 10-378-016), MEM nonessential amino acids (NEAA, cat. no. 11-140-050), HEPES Buffer Solution (cat. no. 15630080), and Sodium Pyruvate (cat. no. 11360070) were purchased from Gibco. Normocin was purchased from Invivogen (cat. no. NC9390718).

#### Lentiviral vectors

A parental lentivector, pMNDW, was utilized to construct the lentiviral vectors employed in this study.^35^^,^^44^ The 2A sequences derived from foot and-mouth disease virus (F2A), porcine teschovirus-1 (P2A), and thosea asigna virus (T2A) were used to link the inserted genes to achieve co-expression. The Lenti/iNKT vector was constructed by inserting into the pMNDW parental vector a synthetic bicistronic gene encoding human iNKT TCRα-F2A-iNKT TCRβ. The Lenti/iNKT-CAR70-IL-15 vector was constructed by inserting into the pMNDW parental vector a synthetic tetracistronic gene encoding human iNKT TCRα-F2A-iNKT TCRβ-P2A-CAR70-T2A-IL15 (CAR70 indicates a CD70-directed CAR, and IL-15 indicates the secreted form of human IL-15). The Lenti/iNKT-BCAR-IL-15 vector was constructed by inserting into the pMNDW parental vector a synthetic tetracistronic gene encoding human iNKT TCRα-F2A-iNKT TCRβ-P2A-BCAR-T2A-IL15 (BCAR indicates a BCMA-directed CAR).^21^^,^^95^ The Lenti/CAR70 vector was constructed by inserting into the pMNDW parental vector a synthetic gene encoding CAR70. The Lenti/CAR70-EGFP vector was constructed by inserting into the pMNDW parental vector a synthetic bicistronic gene encoding CAR70-F2A-EGFP. The Lenti/BCAR vector was constructed by inserting into the pMNDW parental vector a synthetic gene encoding BCAR. The Lenti/FG vector was constructed by inserting into pMNDW parental vector a synthetic bicistronic gene encoding Fluc-P2A-EGFP. The Lenti/CD70 vector was constructed by inserting into pMNDW parental vector a synthetic gene encoding human CD70. Abbreviations used in the lentivector schematic include: ΔLTR, self-inactivating long terminal repeats; MNDU3, internal promoter derived from the MND retroviral LTR U3 region; φ, packaging sequence; RRE, rev-responsive element; cPPT, central polypurine tract; and WPRE, woodchuck hepatitis virus posttranscriptional regulatory element. Synthetic gene fragments were sourced from GenScript (Piscataway, NJ, USA) and IDT (Coralville, IA, USA). Lentiviral particles were generated utilizing HEK 293T cells by employing a standardized transfection procedure with the *Trans*-IT-Lenti Transfection Reagent (Mirus Bio).^35^^,^^44^ Subsequently, a concentration protocol was applied using Amicon TM Ultra Centrifugal Filter Units in accordance with the manufacturer’s specifications (MilliporeSigma).

#### Antibodies and flow cytometry

Fluorochrome-conjugated antibodies specific for human CD45 (Clone HI30, PerCP, FITC, or Pacific Blue-conjugated, 1:500, cat. no. 982318, 982316, or 982306), TCR αβ (Clone IP26, Pacific Blue or PE-Cy7-conjugated, 1:25, cat. no. 306716 or 306720), CD3 (Clone HIT3a, Pacific Blue, PE, or PE-Cy7-conjugated, 1:500, cat. no. 300330, 300308, or 300316), CD1d (Clone 51.1, PE-Cy7 or APC-conjugated, 1:50, cat. no. 350310 or 350308), CD4 (Clone OKT4, PE-Cy7, PerCP or FITC-conjugated, 1:500, cat. no. 317414, 317432 or 317408), CD8 (Clone SK1, PE, APC-Cy7, or APC-conjugated, 1:300, cat. no. 344706, 344714 or 344722), CD14 (Clone HCD14, Pacific Blue-conjugated, 1:100, cat. no. 367122), CD15 (Clone W6D3, APC-conjugated, 1:500, cat. no. 323008), CD19 (Clone HIB19, APC-Cy7-conjugated, 1:200, cat. no. 302218), CD20 (Clone 2H7, APC-Cy7-conjugated, 1:200, cat. no. 302314), CD27 (Clone M-T271, APC or FITC-conjugated, 1:50, cat. no. 986904 or 986910), CD34 (Clone 581, PerCP-conjugated, 1:500, cat. no. 343520), CD31 (Clone WM59, FITC-conjugated, 1:100, cat. no. 989002), CD56 (Clone HCD56, FITC or PerCP-conjugated, 1:10, cat. no. 318304 or 318342), CD69 (Clone FN50, PE-Cy7 or PerCP-conjugated, 1:50, cat. no. 310912 or 310928), CD70 (Clone WM53, APC or PE-conjugated, 1:50, cat. no. 355109 or 355104), CD107a (Clone H4A3, FITC-conjugated, 1:200, cat. no. 338606), CD112 (Clone TX31, PE-conjugated, 1:250, cat. no. 337410), CD155 (Clone SKII.4, PE-Cy7-conjugated, 1:250, cat. no. 337614), CD11b (Clone ICRF44, FITC-conjugated, 1:500, cat. no. 982614), MICA/MICB (Clone 6D4, PE or APC-conjugated, 1:25, cat. no. 320906 or 320908), 41BBL (Clone 5F4, PE-conjugated, 1:500, cat. no. 311504), CD83 (Clone HB15e, APC-Cy7-conjugated, 1:500, cat. no. 305330), CD86 (Clone IT2.2, APC-conjugated, 1:500, cat. no. 305412), CD163 (Clone GHI/61, APC-conjugated, 1:100, cat. no. 333610), CD206 (Clone 15-2, APC-Cy7-conjugated, 1:100, cat. no. 321120), PD-1 (Clone A17188A, PE or FITC-conjugated, 1:25, cat. no. 379210 or 379206), TIM-3 (Clone A18087E, APC-conjugated, 1:25, cat. no. 364804), LAG-3 (Clone 7H2C65, PE-Cy7-conjugated, 1:25, cat. no. 369208), NKG2D (Clone 1D11, PE-Cy7-conjugated, 1:50, cat. no. 320812), DNAM-1 (Clone 11A8, APC-conjugated, 1:50, cat. no. 338312), NKp30 (Clone P30-15, APC-conjugated, 1:50, cat. no. 325210), NKp46 (Clone 9E2, FITC-conjugated, 1:50, cat. no. 137606), CCR2 (Clone K036C2, APC-conjugated, 1:50, cat. no. 357208), CCR5 (Clone J418F1, FITC-conjugated, 1:50, cat. no. 359120), CCR6 (Clone G034E3, PE-conjugated, 1:50, cat. no. 353410), CCR7 (Clone G043H7, APC-Cy7-conjugated, 1:50, cat. no. 353212), CXCR3 (Clone G025H7, PE-Cy7-conjugated, 1:50, cat. no. 353719), CXCR4 (Clone 12G5, APC-conjugated, 1:50, cat. no. 306510), IFN-γ (Clone B27, PE-Cy7-conjugated, 1:50, cat. no. 506518), Granzyme B (Clone QA16A02, APC-conjugated, 1:2000 or 1:5000, cat. no. 372204), Perforin (Clone dG9, PE-Cy7-conjugated, 1:50 or 1:100, cat. no. 308126), TNF-α (Clone MAb11, APC-conjugated, 1:4000, cat. no. 502912), IL-2 (Clone MQ117H12, APC-Cy7-conjugated, 1:50, cat. no. 500342), β2-microglobulin (B2M) (Clone 2M2, FITC or APC-conjugated, 1:2000 or 1:5000, cat. no. 316304 or 316311), HLA-DR (Clone L243, APC-Cy7-conjugated, 1:200 or 1:500, cat. no. 307618), and HLA-DR, DP, DQ (Clone Tü39, FITC-conjugated, 1:200 or 1:500, cat. no. 361706) were purchased from BioLegend. Fluorochrome-conjugated antibodies specific for mouse CD11b (Clone M1/70, APC-conjugated, 1:200, cat. no. 101212), F4/80 (Clone BM8, FITC-conjugated, 1:200, cat. no. 123108), and CD1d (Clone 1B1, APC or PE-Cy7-conjugated, 1:100, cat. no. 123522 or 123524), were purchased from BioLegend. Fluorochrome-conjugated antibodies specific for human iNKT TCR Vɑ24-Jβ18 (Clone 6B11, PE-conjugated, 1:20, cat. no. 552825) was purchased from BD Biosciences. Fluorochrome-conjugated antibodies specific for human fibroblast activation protein FAP (Clone 427819, PE-conjugated, 1:100, cat. no. FAB3715P), ULBP-1 (Clone 170818, PE-conjugated or unconjugated, 1:25, cat. no. FAB1380P or MAB1380), and ULBP-2,5,6 (Clone 165903, APC-conjugated, 1:25, cat. no. FAB1298A) were purchased from R&D Systems. Fluorochrome-conjugated antibodies specific for human TOX (Clone TXRX10, PE-conjugated, 1:50, cat. no. 12-6502-82) was purchased from eBioscience. A goat anti-mouse IgG F(ab’)2 secondary antibody was purchased from ThermoFisher (cat. no. A-11001). Fixable Viability Dye eFluor506 (e506, 1:500, cat. no. 65-0866-14) was purchased from Affymetrix eBioscience; mouse Fc Block (anti-mouse CD16/32, cat. no. 553141) was purchased from BD Biosciences; and human Fc Receptor Blocking Solution (TrueStain FcX) was purchased from BioLegend (cat. no. 422302). In our study, note the use of antibodies with identical clones but differing conjugated fluorochromes, with one typical antibody listed herein.

All flow cytometry staining was performed following standard protocols, as well as specific instructions provided by the manufacturer of a particular antibody. Appropriate isotype staining controls were used for all staining procedures. Stained cells were analyzed using a MACSQuant Analyzer 10 flow cytometer (Miltenyi Biotech), following the manufacturer’s instructions. FlowJo software version 9 (BD Biosciences) was used for data analysis.

#### Enzyme-linked immunosorbent cytokine assays (ELISAs)

The ELISAs for measuring human cytokines were conducted according to a standard protocol provided by BD Biosciences. Supernatants from cell culture experiments were collected and analyzed to quantify cytokines (e.g., human IFN-γ, TNF-α, IL-4, IL-6, and IL-15). The capture and biotinylated antibodies used for cytokine detection were sourced from BD Biosciences, while the streptavidin-HRP conjugate was obtained from Invitrogen. Human cytokine standards were purchased from eBioscience, and the Tetramethylbenzidine (TMB) substrate was acquired from Thermo Scientific (cat. no. PI34021). Human IL-17A ELISA Kits were purchased from Invitrogen (cat. no. BMS2017). Absorbance of the samples was measured at 450 nm using an Infinite M1000 microplate reader (Tecan).

#### Generation of HSPC-engineered allogeneic IL-15-enhanced CD70-directed CAR-engineered NKT (^Allo^CAR70-NKT) cells

Allogeneic HSPC-engineered NKT or CAR-NKT cells, including ^Allo^NKT, ^Allo^BCAR-NKT, and ^Allo^CAR70-NKT cells were generated by differentiating gene engineered human cord blood CD34^+^ HSPCs in a 5-stage clinically guided *Ex Vivo* HSPC-Derived NKT Cell Culture method. The complete methodology and step-by-step protocols have been described in detail in previously published studies.^21^^,^^95^ Here, we provide a summary of the key steps involved in the culture and generation of ^Allo^CAR70-NKT cells.

At Stage 0, the frozen stock of human CD34^+^ HSPCs was thawed and cultured in T cell X-VIVO 15 Serum-Free Hematopoietic Stem Cell Medium supplemented with human Flt3L (50 ng/mL), SCF (50 ng/mL), TPO (50 ng/mL), and IL-3 (20 ng/mL) for 24 h. Lentiviral transduction was then carried out for an additional 24 h using the Lenti/iNKT-CAR70-IL-15 vector.

At Stage 1, gene-engineered HSPCs harvested were cultured in the feeder-free StemSpan SFEM II Medium supplemented with StemSpan Lymphoid Progenitor Expansion Supplement for 14 days. HSPCs were cultured in CELLSTAR24-well Cell Culture Nontreated Multiwell Plates (VWR, cat. no. 82050-892). StemSpan Lymphoid Differentiation Coating Material (500 μL/well, diluted to a final concentration of 1X from a stock dilution of 100X) was applied to the plates and left for 2 h at room temperature or overnight at 4°C. Subsequently, 500 μL of the gene engineered CD34^+^ HSPC suspension, with a density of 2 × 10^4^ cells/ml, was added to each pre-coated well. Half of the medium in each well was removed and replaced with fresh medium twice per week.

At Stage 2, the Stage 1 cells were harvested and cultured in the feeder-free StemSpan SFEM II Medium supplemented with StemSpan Lymphoid Progenitor Maturation Supplement for ∼7 days. StemSpan Lymphoid Differentiation Coating Material (1mL/well, diluted to a final concentration of 1X) was applied to Non-Treated Falcon Polystyrene 6-well Microplates (Thermo Fisher Scientific, cat. no. 140675); 2 mL of the harvested Stage 1 cells, resuspended with a density of 1 × 10^5^ cells/ml, was added into each pre-coated well. The cell density was maintained at 1-2 × 10^6^ cells per well during the Stage 2 culturing. Cells were passaged 2–3 times per week with the addition of fresh medium for each passage.

At Stage 3, the Stage 2 cells were harvested and cultured in the feeder-free StemSpan SFEM II Medium supplemented with StemSpan Lymphoid Progenitor Maturation Supplement, CD3/CD28/CD2 T cell Activator, and human recombinant IL-15 (20 ng/mL) for ∼7 days. StemSpan Lymphoid Differentiation Coating Material (1mL/well, diluted to a final concentration of 1X) was applied to Non-Treated Falcon Polystyrene 6-well Microplates (Thermo Fisher Scientific, cat. no. 08-772-49); 2 mL of the harvested Stage 2 cells, resuspended with a density of 5 × 10^5^ cells/ml, was added into each pre-coated well. The cell density was maintained at 1-2 × 10^6^ cells per well during the Stage 3 culturing. Cells were passaged 2–3 times per week with the addition of fresh medium for each passage.

At Stage 4, the Stage 3 cells were harvested and verified by flow cytometry to confirm their status as mature ^Allo^CAR70-NKT cells or their derivatives; then the cells underwent expansion stage via an aAPC-based expansion. aAPCs were irradiated at 10,000 rads using a Rad Source RS-2000 X-ray Irradiator (Rad Source Technologies). The Stage 3 mature ^Allo^CAR70-NKT cells and derivatives were co-cultured with the irradiated aAPCs (with a ratio of 1:1). The cells were resuspended in expansion medium (the CTS OpTmizer T cell Expansion Serum Free Medium (Thermo Fisher Scientific), or the homemade C10 medium) supplemented with human IL- 7 (10 ng/mL) and IL-15 (10 ng/mL) at a density of 0.5–1 × 10^6^ cells/ml; 2 mL cell suspension was seeded into each well of the Corning Costar Flat Bottom Cell Culture 6-well Plates. The cell density was maintained at 0.5–1 × 10^6^ cells/ml during the expansion stage. Cells were passaged 2–3 times per week with the addition of fresh medium for each passage. The expanded ^Allo^CAR70-NKT cells were aliquoted and cryopreserved in CryoStor Cell Cryopreservation Media CS10 using a Thermo Scientific CryoMed Controlled-Rate Freezer 7450 (Thermo scientific) for stock.

#### Generation of PBMC-derived conventional αβ T (PBMC-T)

PBMCs from healthy donors were utilized to generate conventional αβ T cells, referred to as PBMC-T cells. To produce PBMC-T cells, PBMCs were activated using Dynabeads TM Human T-Activator CD3/CD28 (Thermo Fisher Scientific, cat. no. 11131D) following the manufacturer’s guidelines. The activated cells were then cultured in C10 medium supplemented with 20 ng/mL IL-2 for a duration of 2–3 weeks.

#### Generation of CD70-directed CAR-engineered conventional αβ T (CAR70-T) cells

PBMCs from healthy donors were utilized to generate CAR70-T cells. To produce these cells, non-treated tissue culture 24-well plates (Corning, cat. no. 3738) were coated with Ultra-LEAF Purified Anti-Human CD3 Antibody (Clone OKT3, BioLegend) at 1 μg/mL (500 μL/well), at room temperature for 2 h or at 4°C overnight. PBMCs were resuspended in the C10 medium supplemented with 1 μg/mL Ultra-LEAF Purified Anti-Human CD28 Antibody (Clone CD28.2, BioLegend) and 30 ng/mL IL-2, followed by seeding in the pre-coated plates at 1 × 10^6^ cells/ml (1 mL/well). After 2 days, the cells were transduced with either Lenti/CAR70 or Lenti/CAR70-EGFP viruses for a period of 24 h. The conventional CAR70-T cells were expanded for about 2 weeks in C10 medium and then cryopreserved for future applications.

#### *In vitro* tumor cell killing assay

Various human tumor cells (i.e., MM.1S-FG, MM.1S-CD70-FG, 786-*O*-FG, ACHN-FG, and 786-*O*-FG^CD70−/−^; 1 × 10^4^ cells per well in 96-well plate) were co-cultured with the indicated therapeutic cells (i.e., PBMC-T, CAR70-T, and ^Allo^CAR70-NKT cells) in Corning 96-well clear bottom black plates for 24 h in C10 medium. The effector cell to target cell (E:T) ratio is indicated in the figure legends. At the end of culture, viable tumor cells were quantified by adding D-luciferin (150 μg/mL; Fisher Scientific, cat. no. 50-209-8110) to cell cultures, followed by the measurement of luciferase activity using an Infinite M1000 microplate reader (Tecan). To test NKR mediated killing, 10 μg/mL Ultra-LEAF purified anti-human NKG2D (Clone 1D11, BioLegend, cat. no. 320813) or anti-human DNAM-1 antibody (Clone 11A8, BioLegend, cat. no. 338302) was added to co-cultures to investigate the mechanism of tumor cell killing mediated by NKRs, and LEAF purified mouse lgG2b κ isotype control antibody (Clone MG2b-57, BioLegend, cat. no. 401202) was included as an isotype control. To test CAR mediated killing, 10 μg/mL purified anti-human CD70 antibody (Clone 113-16, BioLegend, cat. no. 355102) was added to co-cultures to investigate the mechanism of tumor cell killing mediated by CARs, and LEAF purified mouse lgG2b κ isotype control antibody (Clone MG2b-57, BioLegend, cat. no. 401202) was included as an isotype control.

#### *In vitro* mixed macrophage/NKT reaction assay

Healthy donor PBMC-derived, M2-polarized macrophages were utilized in this assay. PBMCs were suspended in serum-free RPMI 1640 medium (Corning Cellgro, cat. no. 10-040-CV) at 1 × 10^7^ cells/ml, plated in 10 cm dishes (10–15 mL per dish), and incubated at 37°C with 5% CO_2_ for 1 h. Non-adherent cells were removed, and adherent monocytes were washed twice with PBS and cultured in C10 medium supplemented with recombinant human M-CSF (10 ng/mL, Peprotech, cat. no. 300-25) for 6 days to generate macrophages. On day 6, macrophages were detached using 0.25% Trypsin/EDTA (Gibco, cat. no. 25200-056), collected, and reseeded in 6- or 12-well plates (0.5–1 × 10^6^ cells/ml) for an additional 48 h with recombinant human IL-4 (10 ng/mL, Peprotech, cat. no. 214-14) and IL-13 (10 ng/mL, Peprotech, cat. no. 214-13) to induce polarization. M2-polarized macrophages were then harvested for flow cytometry or co-culture assays.

To set up the *in vitro* mixed macrophage/NKT cell reaction assay, M2-polarized macrophages (1 × 10^5^ cells per well) were seeded into 96-well round-bottom plates and co-cultured with the indicated therapeutic cells (i.e., CAR70-T or ^Allo^CAR70-NKT cells; 1 × 10^5^ cells per well) at a 1:1 ratio in C10 medium. The co-cultures were maintained for 24 h. After co-culture, cells were harvested and analyzed by flow cytometry to assess the viability of macrophages. To test NKT TCR/CD1d mediated macrophage killing, 100 ng/mL αGC and 10 μg/mL Ultra-LEAF purified anti-human CD1d (Clone 51.1, BioLegend, cat. no. 350302) were added to co-cultures, and LEAF purified mouse lgG2b κ isotype control antibody (Clone MG2b-57, BioLegend, cat. no. 401202) was included as an isotype control.

#### *In vitro* tumor organoid targeting assay

Healthy donor PBMC-derived, M2-polarized macrophages were utilized in this assay. To generate tumor organoids, either 2 × 10^5^ 786-O tumor cells alone or a 1:1 mixture of 1 × 10^5^ 786-O tumor cells and 1 × 10^5^ M2 macrophages were resuspended in C10 medium at a concentration of 1 × 10^5^ cells/μl. Cell aggregates were prepared by dispensing 5–10 μL of the cell suspension onto microporous membrane inserts (EMD Millipore, cat. no. PICM0RG50) placed in six-well plates containing 1 mL of C10 medium per well.^19^^,^^61^ After a 2-day incubation period to allow organoid formation, 1 × 10^6^ therapeutic cells (i.e., CAR70-T or ^Allo^CAR70-NKT cells) were resuspended in 100 μL of C10 medium and carefully added on top of each organoid. Co-culture was maintained for 24 h. Following the co-culture period, organoids were mechanically dissociated using a P1000 pipette and passed through a 70-μm nylon strainer to generate single-cell suspensions for downstream flow cytometry analysis. To perform the *in vitro* tumor organoid targeting assay under hypoxic conditions, the same experimental setup was placed in a hypoxia chamber set to 1% O_2_ and 5% CO_2_. Culture plates were incubated in the hypoxic environment for 24 h. After incubation, the organoids were collected and analyzed by flow cytometry.

#### *In vitro* mixed lymphocyte reaction (MLR) assay

Alloreactive T cells were generated by co-culturing 1 × 10^6^ healthy donor T cells with 1 × 10^6^ irradiated, donor-mismatched PBMCs for 2 weeks. Irradiated donor-mismatched PBMCs were replenished every 2 days. To assess the targeting of alloreactive T cells by ^Allo^CAR70-NKT cells, CD70^+^ and CD70^−^ alloreactive T cells were sorted by FACS and subsequently co-cultured with either conventional CAR70-T cells or ^Allo^CAR70-NKT cells at a 1:1 ratio in 96-well round-bottom plates for 8 h in C10 medium. Cytotoxicity was evaluated by flow cytometric quantification of live alloreactive cells (identified as CD3^+^6B11^−^GFP^-^ cells). Culture supernatants were collected for IFN-γ measurement by ELISA.

#### *In vitro* assays using primary RCC patient samples

In one assay, the primary RCC patient samples were analyzed for tumor cell phenotype and the TME composition using flow cytometry. RCC tumor cells identified as CD45^−^CD31^−^FAP (fibroblast activation protein)^-^ cells,^27^^,^^96^ T cells were identified as CD45^high^CD3^+^TCRαβ^+^ cells, CD4 T cells were identified as CD4^+^ T cells, CD8 T cells were identified as CD8^+^ T cells, B cells were identified as CD45^high^CD19^+^ or CD45^high^CD20^+^ cells, NK cells were identified as CD45^high^CD56^+^TCRαβ^−^ cells, myeloid cells were identified as CD45^high^CD11b^+^TCRαβ^−^ cells, granulocytes were identified as CD45^medium^SSC^high^CD11b^+^TCRαβ^−^ cells, tumor-associated macrophages (TAMs) were identified as HLA-DR^high^CD206^high^ myeloid cells, and myeloid-derived suppressor cells (MDSCs) were identified as HLA-DR^low^CD206^low^ myeloid cells. Surface expression of CD70, CD1d, and NK ligands on tumor or/and immune cells were also analyzed using flow cytometry.

In another assay, the primary RCC patient samples were used to study the TME targeting by ^Allo^CAR70-NKT cells. Patient samples were directly co-cultured with ^Allo^CAR70-NKT cells (ratio 1:1) in C10 medium in Corning 96-well Round Bottom Cell Culture plates for 24 h. At the end of culture, cells were collected, and the TME targeting of ^Allo^CAR70-NKT cells was assessed using flow cytometry by quantifying live human TAM/MDSCs (identified as 6B11^−^CD45^+^CD14^+^CD11b^+^ cells), T cells (identified as 6B11^−^CD45^+^CD3^+^ cells), B cells (identified as 6B11^−^CD45^+^CD3^−^CD19^+^ cells or 6B11^−^CD45^+^CD3^−^CD20^+^ cells), and NK cells (identified as 6B11^−^CD45^+^CD3^−^CD56^+^ cells). A total of 15 primary RCC patient samples were included in this assay.

#### 3D tissue imaging

3D tissue imaging involves three key steps: optical tissue clearing, immunostaining, and light-sheet fluorescence imaging. Tissue clearing and immunostaining were performed using the CUBIC (clear, unobstructed brain/body imaging cocktails and computational analysis) protocol.^97^^,^^98^^,^^99^ Samples were first washed in PBS with gentle shaking at room temperature three times: for 3 h during the first two washes and overnight during the third. They were then incubated in 50% CUBIC-L reagent (TCI, T3740) at room temperature for 2 days, followed by incubation in 100% CUBIC-L at 37°C for 6 days. The reagent was refreshed on days 1, 2, and 4.

Subsequently, samples were washed in PBS with gentle shaking at room temperature three times: for 3 h for the first two washes and overnight for the third. RCC tumor cells (RCC#22-FG) were genetically labeled with internal GFP. For immunolabeling of T cells, samples were incubated with an APC-conjugated anti-human CD3 antibody (BioLegend, cat no. 317318; 1:20 dilution) in PBS containing 0.5% Triton X-100. After staining, samples were again washed in PBS with gentle shaking at room temperature three times: for 3 h for the first two washes and overnight for the third. Post-staining fixation was performed in 1% formaldehyde at 4°C for 24 h, followed by an additional incubation at 37°C for 1 h. After fixation, samples were washed again in PBS with gentle shaking at room temperature three times: for 3 h for the first two washes and overnight for the third. Samples were then incubated in 50% CUBIC-R+(M) reagent (TCI, T3741) at room temperature for 2 days, followed by incubation in 100% CUBIC-R+(M) at room temperature for 4 days, with the reagent refreshed on days 1 and 2. Finally, samples were immersed in CUBIC mounting solution (TCI, M3294; refractive index = 1.520) for imaging.

Imaging was conducted using a custom-built refractive index–corrected light-sheet fluorescence microscope (rc-LSFM). GFP-labeled tumor cells were imaged using a 473 nm laser with a 500/40 nm emission filter. APC-labeled T cells were imaged using a 589 nm laser and a 641/75 nm emission filter. Autofluorescence was captured using a 532 nm laser and the same 641/75 nm emission filter.

#### *In vivo* bioluminescence live animal imaging (BLI)

BLI was conducted using the Spectral Advanced Molecular Imaging (AMI) HTX system (Spectral Instrument Imaging). Live animal images were captured 5 min after intraperitoneal (i.p.) administration of D-Luciferin, with doses of 1 mg/mouse for tumor cell (e.g., 786-*O*-FG cell) visualization and 3 mg/mouse for therapeutic cell (e.g., ^Allo^CAR70-NKT/FG cell) visualization. For tissue imaging, experimental mice received an i.p. injection of D-Luciferin with doses of 10 mg/mouse. Mice were then euthanized, and tissues were collected for BLI. The imaging data were processed and analyzed using AURA imaging software (Spectral Instrument Imaging, version 3.2.0).

#### *In vivo* antitumor efficacy study: PDX#22-FG human RCC orthotopic xenograft NSG mouse model

Experimental design is shown in Figure 4A. Briefly, on Day 0, NSG mice received orthotopic inoculation of human RCC PDX#22-FG tumor cells (5 × 10^5^ cells per mouse). The orthotopic implantation of RCC tumor cells is described previously.^100^ On Day 7, the experimental mice received i.v. injection of vehicle (100 μL PBS per mouse), ^Allo^CAR70-NKT cells (10 × 10^6^ CAR70^+^ cells in 100 μL PBS per mouse), or control CAR70-T cells (10 × 10^6^ CAR70^+^ cells in 100 μL PBS per mouse). Over the experiment, mice were monitored for survival and their tumor loads were measured twice per week using BLI. Note that PDX#22-FG represents a CD70-negative/low tumor model, reflecting metastatic RCC with limited CAR target antigen expression.

#### *In vivo* antitumor efficacy study: PDX#22-FG human RCC orthotopic xenograft NSG mouse model and a repeated dosing strategy

Experimental design is shown in Figure S4E. Briefly, on Day 0, NSG mice received orthotopic inoculation of human RCC PDX#22-FG tumor cells (5 × 10^5^ cells per mouse). On Day 7, 14, and 21, the experimental mice received i.v. injection of ^Allo^CAR70-NKT cells (10 × 10^6^ CAR70^+^ cells in 100 μL PBS per mouse). Over the experiment, mice were monitored for survival and their tumor loads were measured twice per week using BLI.

#### *In vivo* antitumor efficacy study: PDX#22-FG human RCC metastatic xenograft NSG mouse model

Experimental design is shown in Figure 5A. Briefly, on Day 0, NSG mice received intravenous (i.v.) inoculation of human RCC PDX#22-FG tumor cells (5 × 10^5^ cells per mouse). On Day 5, the experimental mice received i.v. injection of vehicle (100 μL PBS per mouse), ^Allo^CAR70-NKT cells (10 × 10^6^ CAR70^+^ cells in 100 μL PBS per mouse), or control CAR70-T cells (10 × 10^6^ CAR70^+^ cells in 100 μL PBS per mouse). Over the experiment, mice were monitored for survival and their tumor loads were measured twice per week using BLI.

#### *In vivo* antitumor efficacy study: 786-*O*-FG human RCC metastatic xenograft NSG mouse model

Experimental design is shown in Figure 5E. Briefly, on Day 0, NSG mice received intravenous (i.v.) inoculation of human RCC 786-*O*-FG tumor cells (5 × 10^5^ cells per mouse). On Day 5, the experimental mice received i.v. injection of vehicle (100 μL PBS per mouse), ^Allo^CAR70-NKT cells (10 × 10^6^ CAR70^+^ cells in 100 μL PBS per mouse), or control CAR70-T cells (10 × 10^6^ CAR70^+^ cells in 100 μL PBS per mouse). Over the experiment, mice were monitored for survival and their tumor loads were measured twice per week using BLI.

#### *In vivo* antitumor efficacy study: 786-*O*-FG^CD70−/−^ human RCC metastatic xenograft NSG mouse model

Experimental design is shown in Figure 5I. Briefly, on Day 0, NSG mice received intravenous (i.v.) inoculation of human RCC 786-*O*-FG^CD70−/−^ tumor cells (5 × 10^5^ cells per mouse). This experimental setup was designed to recapitulate the phenomenon of CAR antigen escape in RCC tumors. On Day 5, the experimental mice received i.v. injection of vehicle (100 μL PBS per mouse), ^Allo^CAR70-NKT cells (10 × 10^6^ CAR70^+^ cells in 100 μL PBS per mouse), or control CAR70-T cells (10 × 10^6^ CAR70^+^ cells in 100 μL PBS per mouse). Over the experiment, mice were monitored for survival and their tumor loads were measured twice per week using BLI.

#### *In vivo* TAM depletion assay

Experimental design is shown in Figure 6N. Briefly, on Day 0, NSG mice received subcutaneous (s.c.) inoculation of human RCC PDX#22-FG tumor cells (1 × 10^6^ cells per mouse). On Day 5, the experimental mice received paratumoral injection of vehicle (100 μL PBS per mouse), ^Allo^CAR70-NKT cells (10 × 10^6^ CAR70^+^ cells in 100 μL PBS per mouse), or control CAR70-T cells (10 × 10^6^ CAR70^+^ cells in 100 μL PBS per mouse). On Day 12, tumors were collected for FACS analysis.

#### *In vivo* alloreactive T cell xenograft NSG mouse model

Experimental design is shown in Figure 7E. Briefly, on Day 0, NSG mice received i.v. injection of CAR70-T/FG cells (5 × 10^6^ cells in 100 μL PBS per mouse), or ^Allo^CAR70-NKT/FG cells (5 × 10^6^ cells in 100 μL PBS per mouse). On Day 3, mice received i.v. injection of donor-mismatched alloreactive T cells (5 × 10^6^ cells in 100 μL PBS per mouse). Over the experiment, mice were monitored to assess the persistence and biodistribution of therapeutic cell using BLI.

#### *In vivo* cytokine release syndrome (CRS) evaluation in NSG mouse model

Experimental design is shown in Figure S7A. Briefly, on Day 0, NSG mice received intraperitoneal (i.p.) inoculation of 786-*O*-FG cells (10 × 10^6^ cells per mouse). On Day 10, the experimental mice received i.p. injection of vehicle (100 μL PBS per mouse), ^Allo^CAR70-NKT cells (10 × 10^6^ CAR^+^ cells in 100 μL PBS per mouse), or control CAR70-T cells (10 × 10^6^ CAR^+^ cells in 100 μL PBS per mouse). On Days 13, blood samples were collected from the experimental mice, and their serum cytokines (i.e., mouse IL-6, human IFN-γ, TNF-α, and IL-6) and mouse SAA-3 were measured using ELISA. A Mouse SAA-3 ELISA Kit (Millipore Sigma) was used to measure SAA-3, following the manufacturer’s instructions.

#### *In vivo* CRS evaluation in NSG-SGM3 mouse model

Experimental design is shown in Figure S7F. Briefly, on Day 0, NSG-SGM mice received i.p. inoculation of 786-*O*-FG cells (10 × 10^6^ cells per mouse). On Day 3, 7, 10, and 12, the experimental mice received i.p. injection of healthy donor PBMC-derived CD14^+^ myeloid cells. On Day 10, the experimental mice received i.p. injection of vehicle (100 μL PBS per mouse), ^Allo^CAR70-NKT cells (10 × 10^6^ CAR^+^ cells in 100 μL PBS per mouse), or control CAR70-T cells (10 × 10^6^ CAR^+^ cells in 100 μL PBS per mouse). Note that in this study, both CAR70-T cells and CD14^+^ myeloid cells were derived from the same PBMC donors, thereby preventing alloreactive responses between the CAR70-T cells and the myeloid compartment. On Days 13, blood samples were collected from the experimental mice, and their serum cytokines (i.e., human IL-6) and mouse SAA-3 were measured using ELISA. In addition, peritoneal fluid samples were collected, and cellular composition and phenotype were analyzed by flow cytometry.

#### *In vivo* organ damage evaluation

Experimental design is shown in Figure S7L. On Day 0, NSG mice received i.v. injection of vehicle (100 μL PBS per mouse), ^Allo^CAR70-NKT cells (10 × 10^6^ CAR^+^ cells in 100 μL PBS per mouse), or control CAR70-T cells (10 × 10^6^ CAR^+^ cells in 100 μL PBS per mouse). On Day 1, 20, and 40, blood samples were collected from the experimental mice, and the organ damage biomarkers were measured using ELISA. Urea Nitrogen (BUN) Colorimetric Detection Kit was purchased from ThermoFisher Scientific (cat. no. EIABUN). Mouse AST ELISA kit was purchased from Abcam (cat. no. ab263882). Mouse ALT ELISA kit was purchased from Abcam (cat. no. ab282882). Mouse Bilirubin ELISA Kit was purchased from MyBioSource (cat. no. MBS3805359). Mouse Glutamate dehydrogenase (GLDH) ELISA Kit was purchased from MyBioSource (cat. no. MBS761948).

On Day 120, various mouse tissues were collected and analyzed by the UCLA Pathology Core. Tissues were analyzed for inflammation (Inf), hematopoietic neoplasm (HN), and nonhematopoietic neoplasm (NHN). Data were presented as pathologist’s scores of individual mouse tissues (*n* = 5). 0, no abnormal findings; 1, mild; 2, moderate; 3, severe.

### Quantification and statistical analysis

Graphpad Prism 9 software (Graphpad) was used for statistical data analysis. Student’s two-tailed t test was used for pairwise comparisons. Ordinary 1-way ANOVA followed by Tukey’s or Dunnett’s multiple comparisons test was used for multiple comparisons. Log rank (Mantel-Cox) test adjusted for multiple comparisons was used for Meier survival curves analysis. Data are presented as the mean ± SEM, unless otherwise indicated. In all figures and figure legends, “n” represents the number of samples or animals used in the indicated experiments. A *p* value of less than 0.05 was considered significant. ns, not significant.

## Supplemental information

Document S1. Figures S1–S7 and Table S1

Document S2. Article plus supplemental information

## References

[1] Hsieh J.J., Purdue M.P., Signoretti S., Swanton C., Albiges L., Schmidinger M., Heng D.Y., Larkin J., Ficarra V. (2017). Renal cell carcinoma. Nat. Rev. Dis. Primers.

[2] Anselmo Da Costa I., Rausch S., Kruck S., Todenhöfer T., Stenzl A., Bedke J. (2017). Immunotherapeutic strategies for the treatment of renal cell carcinoma: Where will we go? Expert Rev. Anticancer Ther..

[3] Padala S.A., Barsouk A., Thandra K.C., Saginala K., Mohammed A., Vakiti A., Rawla P., Barsouk A. (2020). Epidemiology of Renal Cell Carcinoma. World J. Oncol..

[4] Flanigan R.C., Campbell S.C., Clark J.I., Picken M.M. (2003). Metastatic renal cell carcinoma. Curr. Treat. Options Oncol..

[5] Vuong L., Kotecha R.R., Voss M.H., Hakimi A.A. (2019). Tumor Microenvironment Dynamics in Clear-Cell Renal Cell Carcinoma. Cancer Discov..

[6] Yang M., Tang X., Zhang Z., Gu L., Wei H., Zhao S., Zhong K., Mu M., Huang C., Jiang C. (2020). Tandem CAR-T cells targeting CD70 and B7-H3 exhibit potent preclinical activity against multiple solid tumors. Theranostics.

[7] Xiong Q., Wang H., Shen Q., Wang Y., Yuan X., Lin G., Jiang P. (2024). The development of chimeric antigen receptor T-cells against CD70 for renal cell carcinoma treatment. J. Transl. Med..

[8] Junker K., Hindermann W., von Eggeling F., Diegmann J., Haessler K., Schubert J. (2005). CD70: a new tumor specific biomarker for renal cell carcinoma. J. Urol..

[9] Pal S.K., Tran B., Haanen J.B.A.G., Hurwitz M.E., Sacher A., Tannir N.M., Budde L.E., Harrison S.J., Klobuch S., Patel S.S. (2024). CD70-Targeted Allogeneic CAR T-Cell Therapy for Advanced Clear Cell Renal Cell Carcinoma. Cancer Discov..

[10] Wagner K., Siska P.J. (2024). “Living drugs” target CD70 in advanced renal tumors. Trends Pharmacol. Sci..

[11] Li Y.-R., Halladay T., Yang L. (2024). Immune evasion in cell-based immunotherapy: unraveling challenges and novel strategies. J. Biomed. Sci..

[12] Larson R.C., Maus M.V. (2021). Recent advances and discoveries in the mechanisms and functions of CAR T cells. Nat. Rev. Cancer.

[13] Depil S., Duchateau P., Grupp S.A., Mufti G., Poirot L. (2020). Off-the-shelf’ allogeneic CAR T cells: development and challenges. Nat. Rev. Drug Discov..

[14] Li Y.-R., Fang Y., Niu S., Chen Y., Lyu Z., Yang L. (2025). Managing allorejection in off-the-shelf CAR-engineered cell therapies. Mol. Ther..

[15] Li Y.-R., Zhu Y., Halladay T., Yang L. (2025). In vivo CAR engineering for immunotherapy. Nat. Rev. Immunol..

[16] Wang Y., Wang S., Li N. (2025). Accelerating the clinical translation of CD70-targeted chimeric antigen receptor-based cell therapies in oncology: A comprehensive clinical investigation panorama analysis based on the Trialtrove database. Cancer Lett..

[17] Wang L., Wang Y., He X., Mo Z., Zhao M., Liang X., Hu K., Wang K., Yue Y., Mo G. (2025). CD70-targeted iPSC-derived CAR-NK cells display potent function against tumors and alloreactive T cells. Cell reports. Med.

[18] Mier J.W. (2019). The tumor microenvironment in renal cell cancer. Curr. Opin. Oncol..

[19] Li Y.-R., Brown J., Yu Y., Lee D., Zhou K., Dunn Z.S., Hon R., Wilson M., Kramer A., Zhu Y. (2022). Targeting Immunosuppressive Tumor-Associated Macrophages Using Innate T Cells for Enhanced Antitumor Reactivity. Cancers.

[20] Li Y.-R., Wilson M., Yang L. (2022). Target tumor microenvironment by innate T cells. Front. Immunol..

[21] Li Y.-R., Zhou Y., Yu J., Kim Y.J., Li M., Lee D., Zhou K., Chen Y., Zhu Y., Wang Y.-C. (2025). Generation of allogeneic CAR-NKT cells from hematopoietic stem and progenitor cells using a clinically guided culture method. Nat. Biotechnol..

[22] Li Y.-R., Fang Y., Lyu Z., Zhu Y., Yang L. (2023). Exploring the dynamic interplay between cancer stem cells and the tumor microenvironment: implications for novel therapeutic strategies. J. Transl. Med..

[23] Heidegger I., Pircher A., Pichler R. (2019). Targeting the Tumor Microenvironment in Renal Cell Cancer Biology and Therapy. Front. Oncol..

[24] Kronenberg M. (2005). Toward an understanding of NKT cell biology: progress and paradoxes. Annu. Rev. Immunol..

[25] Bae E.A., Seo H., Kim I.K., Jeon I., Kang C.Y. (2019). Roles of NKT cells in cancer immunotherapy. Arch Pharm. Res. (Seoul).

[26] Bendelac A., Savage P.B., Teyton L. (2007). The Biology of NKT Cells. Annu. Rev. Immunol..

[27] Li Y.-R., Ochoa C.J., Zhu Y., Kramer A., Wilson M., Fang Y., Chen Y., Singh T., Di Bernardo G., Zhu E. (2023). Profiling ovarian cancer tumor and microenvironment during disease progression for cell-based immunotherapy design. iScience.

[28] Courtney A., Liu D., Wei J., Gao X., Marinova E., Asgharzadeh S., Metelitsa L. (2012). M2 macrophages express CD1d and are selectively targeted by NKT cells in tumors (127.39). J. Immunol..

[29] Metelitsa L.S. (2011). Anti-tumor potential of type-I NKT cells against CD1d-positive and CD1d-negative tumors in humans. Clin. Immunol..

[30] Grewal I.S. (2008). CD70 as a therapeutic target in human malignancies. Expert Opin. Ther. Targets.

[31] Bowman M.R., Crimmins M.A., Yetz-Aldape J., Kriz R., Kelleher K., Herrmann S. (1994). The cloning of CD70 and its identification as the ligand for CD27. J. Immunol..

[32] Heczey A., Xu X., Courtney A.N., Tian G., Barragan G.A., Guo L., Amador C.M., Ghatwai N., Rathi P., Wood M.S. (2023). Anti-GD2 CAR-NKT cells in relapsed or refractory neuroblastoma: updated phase 1 trial interim results. Nat. Med..

[33] Heczey A., Courtney A.N., Montalbano A., Robinson S., Liu K., Li M., Ghatwai N., Dakhova O., Liu B., Raveh-Sadka T. (2020). Anti-GD2 CAR-NKT cells in patients with relapsed or refractory neuroblastoma: an interim analysis. Nat. Med..

[34] Pellicci D.G., Koay H.-F., Berzins S.P. (2020). Thymic development of unconventional T cells: how NKT cells, MAIT cells and γδ T cells emerge. Nat. Rev. Immunol..

[35] Zhu Y., Smith D.J., Zhou Y., Li Y.R., Yu J., Lee D., Wang Y.C., Di Biase S., Wang X., Hardoy C. (2019). Development of Hematopoietic Stem Cell-Engineered Invariant Natural Killer T Cell Therapy for Cancer. Cell Stem Cell.

[36] Neelapu S.S., Tummala S., Kebriaei P., Wierda W., Gutierrez C., Locke F.L., Komanduri K.V., Lin Y., Jain N., Daver N. (2018). Chimeric antigen receptor T-cell therapy - assessment and management of toxicities. Nat. Rev. Clin. Oncol..

[37] Prasad V. (2018). Immunotherapy: Tisagenlecleucel - the first approved CAR-T-cell therapy: implications for payers and policy makers. Nat. Rev. Clin. Oncol..

[38] Rafiq S., Hackett C.S., Brentjens R.J. (2020). Engineering strategies to overcome the current roadblocks in CAR T cell therapy. Nat. Rev. Clin. Oncol..

[39] Amini L., Silbert S.K., Maude S.L., Nastoupil L.J., Ramos C.A., Brentjens R.J., Sauter C.S., Shah N.N., Abou-El-Enein M. (2022). Preparing for CAR T cell therapy: patient selection, bridging therapies and lymphodepletion. Nat. Rev. Clin. Oncol..

[40] Cappell K.M., Kochenderfer J.N. (2023). Long-term outcomes following CAR T cell therapy: what we know so far. Nat. Rev. Clin. Oncol..

[41] Srinivasan S., Tan N., Cheng H.-Y., Zhang Y., Tacheva-Grigorova S., Van Blarcom T., Sommer C., Nguyen D., Sasu B., Panowski S. (2020). Investigation of ALLO-316: A Fratricide-Resistant Allogeneic CAR T Targeting CD70 As a Potential Therapy for the Treatment of AML. Blood.

[42] Cheng J., Zhao Y., Hu H., Tang L., Zeng Y., Deng X., Ding S., Guo A.-Y., Li Q., Zhu X. (2023). Revealing the impact of CD70 expression on the manufacture and functions of CAR-70 T-cells based on single-cell transcriptomics. Cancer Immunol. Immunother..

[43] De Munter S., Buhl J.L., De Cock L., Van Parys A., Daneels W., Pascal E., Deseins L., Ingels J., Goetgeluk G., Jansen H. (2024). Knocking Out CD70 Rescues CD70-Specific NanoCAR T Cells from Antigen-Induced Exhaustion. Cancer Immunol. Res..

[44] Li Y.-R., Zhou Y., Kim Y.J., Zhu Y., Ma F., Yu J., Wang Y.-C., Chen X., Li Z., Zeng S. (2021). Development of allogeneic HSC-engineered iNKT cells for off-the-shelf cancer immunotherapy. Cell reports. Med.

[45] Courtney A.N., Tian G., Metelitsa L.S. (2023). Natural killer T cells and other innate-like T lymphocytes as emerging platforms for allogeneic cancer cell therapy. Blood.

[46] Rotolo A., Caputo V.S., Holubova M., Baxan N., Dubois O., Chaudhry M.S., Xiao X., Goudevenou K., Pitcher D.S., Petevi K. (2018). Enhanced Anti-lymphoma Activity of CAR19-iNKT Cells Underpinned by Dual CD19 and CD1d Targeting. Cancer Cell.

[47] Bollino D., Webb T.J. (2017). Chimeric antigen receptor-engineered natural killer and natural killer T cells for cancer immunotherapy. Transl. Res..

[48] Gordy L.E., Bezbradica J.S., Flyak A.I., Spencer C.T., Dunkle A., Sun J., Stanic A.K., Boothby M.R., He Y.-W., Zhao Z. (2011). IL-15 regulates homeostasis and terminal maturation of NKT cells. J. Immunol..

[49] Liu D., Song L., Wei J., Courtney A.N., Gao X., Marinova E., Guo L., Heczey A., Asgharzadeh S., Kim E. (2012). IL-15 protects NKT cells from inhibition by tumor-associated macrophages and enhances antimetastatic activity. J. Clin. Investig..

[50] Ohteki T. (2002). Critical role for IL-15 in innate immunity. Curr. Mol. Med..

[51] Sun C., Dotti G., Savoldo B. (2016). Utilizing cell-based therapeutics to overcome immune evasion in hematologic malignancies. Blood.

[52] Van der Vreken A., Vanderkerken K., De Bruyne E., De Veirman K., Breckpot K., Menu E. (2024). Fueling CARs: metabolic strategies to enhance CAR T-cell therapy. Exp. Hematol. Oncol..

[53] Sterner R.C., Sterner R.M. (2021). CAR-T cell therapy: current limitations and potential strategies. Blood Cancer J..

[54] Rabinovich G.A., Gabrilovich D., Sotomayor E.M. (2007). Immunosuppressive strategies that are mediated by tumor cells. Annu. Rev. Immunol..

[55] Majzner R.G., Mackall C.L. (2018). Tumor Antigen Escape from CAR T-cell Therapy. Cancer Discov..

[56] Grisanzio C., Seeley A., Chang M., Collins M., Di Napoli A., Cheng S.-C., Percy A., Beroukhim R., Signoretti S. (2011). Orthotopic xenografts of RCC retain histological, immunophenotypic and genetic features of tumours in patients. J. Pathol..

[57] Nanus D.M., Engelstein D., Mydlo J.H. (2001). Orthotopic Model of Renal Cell Carcinoma BT - Renal Cancer: Methods and Protocols.

[58] Hu J., Tan P., Ishihara M., Bayley N.A., Schokrpur S., Reynoso J.G., Zhang Y., Lim R.J., Dumitras C., Yang L. (2023). Tumor heterogeneity in VHL drives metastasis in clear cell renal cell carcinoma. Signal Transduct. Target. Ther..

[59] Kohli K., Pillarisetty V.G., Kim T.S. (2022). Key chemokines direct migration of immune cells in solid tumors. Cancer Gene Ther..

[60] Alrumaihi F. (2022). The Multi-Functional Roles of CCR7 in Human Immunology and as a Promising Therapeutic Target for Cancer Therapeutics. Front. Mol. Biosci..

[61] Li Y.-R., Yu Y., Kramer A., Hon R., Wilson M., Brown J., Yang L. (2022). An Ex Vivo 3D Tumor Microenvironment-Mimicry Culture to Study TAM Modulation of Cancer Immunotherapy. Cells.

[62] Chen Z., Han F., Du Y., Shi H., Zhou W. (2023). Hypoxic microenvironment in cancer: molecular mechanisms and therapeutic interventions. Signal Transduct. Target. Ther..

[63] Matsuda J.L., Mallevaey T., Scott-Browne J., Gapin L. (2008). CD1d-restricted iNKT cells, the “Swiss-Army knife” of the immune system. Curr. Opin. Immunol..

[64] Diorio C., Teachey D.T., Grupp S.A. (2025). Allogeneic chimeric antigen receptor cell therapies for cancer: progress made and remaining roadblocks. Nat. Rev. Clin. Oncol..

[65] Li Y.-R., Fang Y., Niu S., Zhu Y., Chen Y., Lyu Z., Zhu E., Tian Y., Huang J., Rezek V. (2025). Allogeneic CD33-directed CAR-NKT cells for the treatment of bone marrow-resident myeloid malignancies. Nat. Commun..

[66] Li Y.-R., Zhou Y., Yu J., Zhu Y., Lee D., Zhu E., Li Z., Kim Y.J., Zhou K., Fang Y. (2024). Engineering Allorejection-Resistant CAR-NKT Cells from Hematopoietic Stem Cells for Off-The-Shelf Cancer Immunotherapy. Mol. Ther..

[67] Li Y.-R., Dunn Z.S., Zhou Y., Lee D., Yang L. (2021). Development of Stem Cell-Derived Immune Cells for Off-the-Shelf Cancer Immunotherapies. Cells.

[68] Sommer C., Boldajipour B., Kuo T.C., Bentley T., Sutton J., Chen A., Geng T., Dong H., Galetto R., Valton J. (2019). Preclinical Evaluation of Allogeneic CAR T Cells Targeting BCMA for the Treatment of Multiple Myeloma. Mol. Ther..

[69] Lv Z., Luo F., Chu Y. (2023). Strategies for overcoming bottlenecks in allogeneic CAR-T cell therapy. Front. Immunol..

[70] Coman T., Rossignol J., D’Aveni M., Fabiani B., Dussiot M., Rignault R., Babdor J., Bouillé M., Herbelin A., Coté F. (2018). Human CD4- invariant NKT lymphocytes regulate graft versus host disease. OncoImmunology.

[71] Haraguchi K., Takahashi T., Hiruma K., Kanda Y., Tanaka Y., Ogawa S., Chiba S., Miura O., Sakamaki H., Hirai H. (2004). Recovery of Valpha24+ NKT cells after hematopoietic stem cell transplantation. Bone Marrow Transplant..

[72] Li Y.-R., Zhou K., Zhu Y., Halladay T., Yang L. (2025). Breaking the mold: Unconventional T cells in cancer therapy. Cancer Cell.

[73] Norelli M., Camisa B., Barbiera G., Falcone L., Purevdorj A., Genua M., Sanvito F., Ponzoni M., Doglioni C., Cristofori P. (2018). Monocyte-derived IL-1 and IL-6 are differentially required for cytokine-release syndrome and neurotoxicity due to CAR T cells. Nat. Med..

[74] Giavridis T., Van Der Stegen S.J.C., Eyquem J., Hamieh M., Piersigilli A., Sadelain M. (2018). CAR T cell-induced cytokine release syndrome is mediated by macrophages and abated by IL-1 blockade letter. Nat. Med..

[75] Yang S., Xu J., Dai Y., Jin S., Sun Y., Li J., Liu C., Ma X., Chen Z., Chen L. (2024). Neutrophil activation and clonal CAR-T re-expansion underpinning cytokine release syndrome during ciltacabtagene autoleucel therapy in multiple myeloma. Nat. Commun..

[76] Boulch M., Cazaux M., Cuffel A., Ruggiu M., Allain V., Corre B., Loe-Mie Y., Hosten B., Cisternino S., Auvity S. (2023). A major role for CD4(+) T cells in driving cytokine release syndrome during CAR T cell therapy. Cell Rep. Med..

[77] Sica A., Larghi P., Mancino A., Rubino L., Porta C., Totaro M.G., Rimoldi M., Biswas S.K., Allavena P., Mantovani A. (2008). Macrophage polarization in tumour progression. Semin. Cancer Biol..

[78] Yunna C., Mengru H., Lei W., Weidong C. (2020). Macrophage M1/M2 polarization. Eur. J. Pharmacol..

[79] Zang P.D., Angeles A., Pal S.K. (2025). CD70: An Emerging Anticancer Target in Renal Cell Carcinoma and Beyond. Annu. Rev. Med..

[80] Kim T.J., Lee Y.H., Koo K.C. (2022). Current and future perspectives on CAR-T cell therapy for renal cell carcinoma: A comprehensive review. Investig. Clin. Urol..

[81] Finke J.H., Rini B., Ireland J., Rayman P., Richmond A., Golshayan A., Wood L., Elson P., Garcia J., Dreicer R., Bukowski R. (2008). Sunitinib reverses type-1 immune suppression and decreases T-regulatory cells in renal cell carcinoma patients. Clin. Cancer Res..

[82] Atkins M.B., Plimack E.R., Puzanov I., Fishman M.N., McDermott D.F., Cho D.C., Vaishampayan U., George S., Olencki T.E., Tarazi J.C. (2018). Axitinib in combination with pembrolizumab in patients with advanced renal cell cancer: a non-randomised, open-label, dose-finding, and dose-expansion phase 1b trial. Lancet Oncol..

[83] Rotolo A., Whelan E.C., Atherton M.J., Kulikovskaya I., Jarocha D., Fraietta J.A., Kim M.M., Diffenderfer E.S., Cengel K.A., Piviani M. (2023). Unedited allogeneic iNKT cells show extended persistence in MHC-mismatched canine recipients. Cell Rep. Med..

[84] Wei W., Grünwald V., Herrmann K. (2025). CD70-targeted cancer theranostics: Progress and challenges. Med.

[85] Lanza R., Russell D.W., Nagy A. (2019). Engineering universal cells that evade immune detection. Nat. Rev. Immunol..

[86] DiNofia A.M., Grupp S.A. (2021). Will allogeneic CAR T cells for CD19(+) malignancies take autologous CAR T cells “off the shelf”. Nat. Rev. Clin. Oncol..

[87] Li Y.-R., Zhu Y., Fang Y., Lyu Z., Yang L. (2025). Emerging trends in clinical allogeneic CAR cell therapy. Med.

[88] Elsallab M., Maus M.V. (2023). Expanding access to CAR T cell therapies through local manufacturing. Nat. Biotechnol..

[89] Steffin D., Ghatwai N., Montalbano A., Rathi P., Courtney A.N., Arnett A.B., Fleurence J., Sweidan R., Wang T., Zhang H. (2025). Interleukin-15-armoured GPC3 CAR T cells for patients with solid cancers. Nature.

[90] Li Y.-R., Zhu Y., Yang L. (2025). IL-15 in CAR engineering: striking an efficacy-safety balance. Trends Mol. Med..

[91] Li Y.-R., Lyu Z., Chen Y., Fang Y., Yang L. (2024). Frontiers in CAR-T cell therapy for autoimmune diseases. Trends Pharmacol. Sci..

[92] Han B.K., Olsen N.J., Bottaro A. (2016). The CD27-CD70 pathway and pathogenesis of autoimmune disease. Semin. Arthritis Rheum..

[93] Dhaeze T., Tremblay L., Lachance C., Peelen E., Zandee S., Grasmuck C., Bourbonnière L., Larouche S., Ayrignac X., Rébillard R.-M. (2019). CD70 defines a subset of proinflammatory and CNS-pathogenic T(H)1/T(H)17 lymphocytes and is overexpressed in multiple sclerosis. Cell. Mol. Immunol..

[94] Giannoni F., Hardee C.L., Wherley J., Gschweng E., Senadheera S., Kaufman M.L., Chan R., Bahner I., Gersuk V., Wang X. (2013). Allelic exclusion and peripheral reconstitution by TCR transgenic T cells arising from transduced human hematopoietic stem/progenitor cells. Mol. Ther..

[95] Li Y.-R., Zhou K., Lee D., Zhu Y., Halladay T., Yu J., Zhou Y., Lyu Z., Fang Y., Chen Y. (2025). Generating allogeneic CAR-NKT cells for off-the-shelf cancer immunotherapy with genetically engineered HSP cells and feeder-free differentiation culture. Nat. Protoc..

[96] Li Y.-R., Ochoa C.J., Lyu Z., Zhu Y., Chen Y., DiBernardo G., Ruegg L., Memarzadeh S., Yang L. (2025). Protocol to profile tumor and microenvironment from ovarian cancer patient samples and evaluate cell-based therapy using in vitro killing assays. STAR Protoc..

[97] Tainaka K., Murakami T.C., Susaki E.A., Shimizu C., Saito R., Takahashi K., Hayashi-Takagi A., Sekiya H., Arima Y., Nojima S. (2018). Chemical Landscape for Tissue Clearing Based on Hydrophilic Reagents. Cell Rep..

[98] Tainaka K., Kubota S.I., Suyama T.Q., Susaki E.A., Perrin D., Ukai-Tadenuma M., Ukai H., Ueda H.R. (2014). Whole-body imaging with single-cell resolution by tissue decolorization. Cell.

[99] Zhu E., Zhang Y., Zhao P., Cho J.M., Wang Z., Li Y.-R., Wang J., Margolis S., Wang S., Yang L. (2025). Refractive Index-Corrected Light-Sheet Microscopy for Macro-View Cardiovascular Imaging. Adv. Sci..

[100] Schokrpur S., Hu J., Moughon D.L., Liu P., Lin L.C., Hermann K., Mangul S., Guan W., Pellegrini M., Xu H., Wu L. (2016). CRISPR-Mediated VHL Knockout Generates an Improved Model for Metastatic Renal Cell Carcinoma. Sci. Rep..

