# Supplementary material for: Multimodal targeting of metastatic renal cell carcinoma via CD70-directed allogeneic CAR-NKT cells
Source: Cell Rep Med. 2025 Aug 29;6(9):102321. doi: 10.1016/j.xcrm.2025.102321 (PMC12490242; doi:10.1016/j.xcrm.2025.102321)
Supplement: Document S1. Figures S1–S7 and Table S1 [file mmc1.pdf]

Cell Reports Medicine, Volume *6*

Supplemental information

Multimodal targeting of metastatic renal cell carcinoma via CD70-directed allogeneic CAR-NKT cells

Yan-Ruide Li, Junhui Hu, Zhe Li, Enbo Zhu, Yuning Chen, Tyler Halladay, Xinyuan Shen, Ying Fang, Yichen Zhu, Zibai Lyu, Yanxin Tian, Jie Huang, Annabel S. Zhao, Nathan Y. Ma, Catherine Zhang, Yongpeng Xie, Hanwei Zhang, Tzung Hsiai, Arnold I. Chin, Lily Wu, and Lili Yang

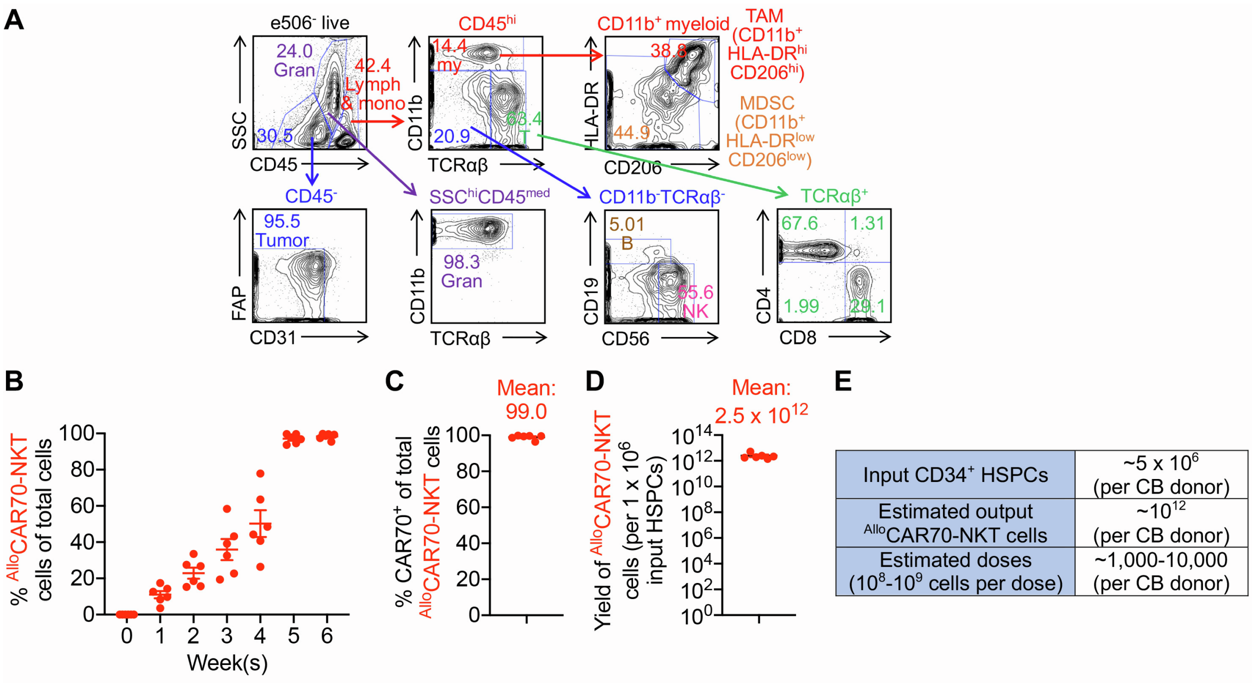

Figure S1. FACS gating strategies and the generation of ^{Allo}CAR70-NKT cells. Related to Figures 1 and 2.

(A) FACS gating strategies for RCC tumor cells and immune cells in the TME. Gran, granulocyte; lymph, lymphocyte; mono, monocyte; my, myeloid cell; TAM, tumor-associated macrophage; MDSC, myeloid-derived suppressor cell; NK, natural killer cell.

(B-E) Generation of ^{Allo}CAR70-NKT cells. (B) Percentage of ^{Allo}CAR70-NKT cells in total live cells during the 6-week culture (n = 6; n indicates different CB donors). (C) CAR70 expression on ^{Allo}CAR70-NKT cells (n = 6; n indicates different CB donors). (D) Yield of ^{Allo}CAR70-NKT cells (n = 6; n indicates different CB donors). (E) Table showing the estimated output cell numbers and doses of ^{Allo}CAR70-NKT cells.

Representative of over 6 experiments.

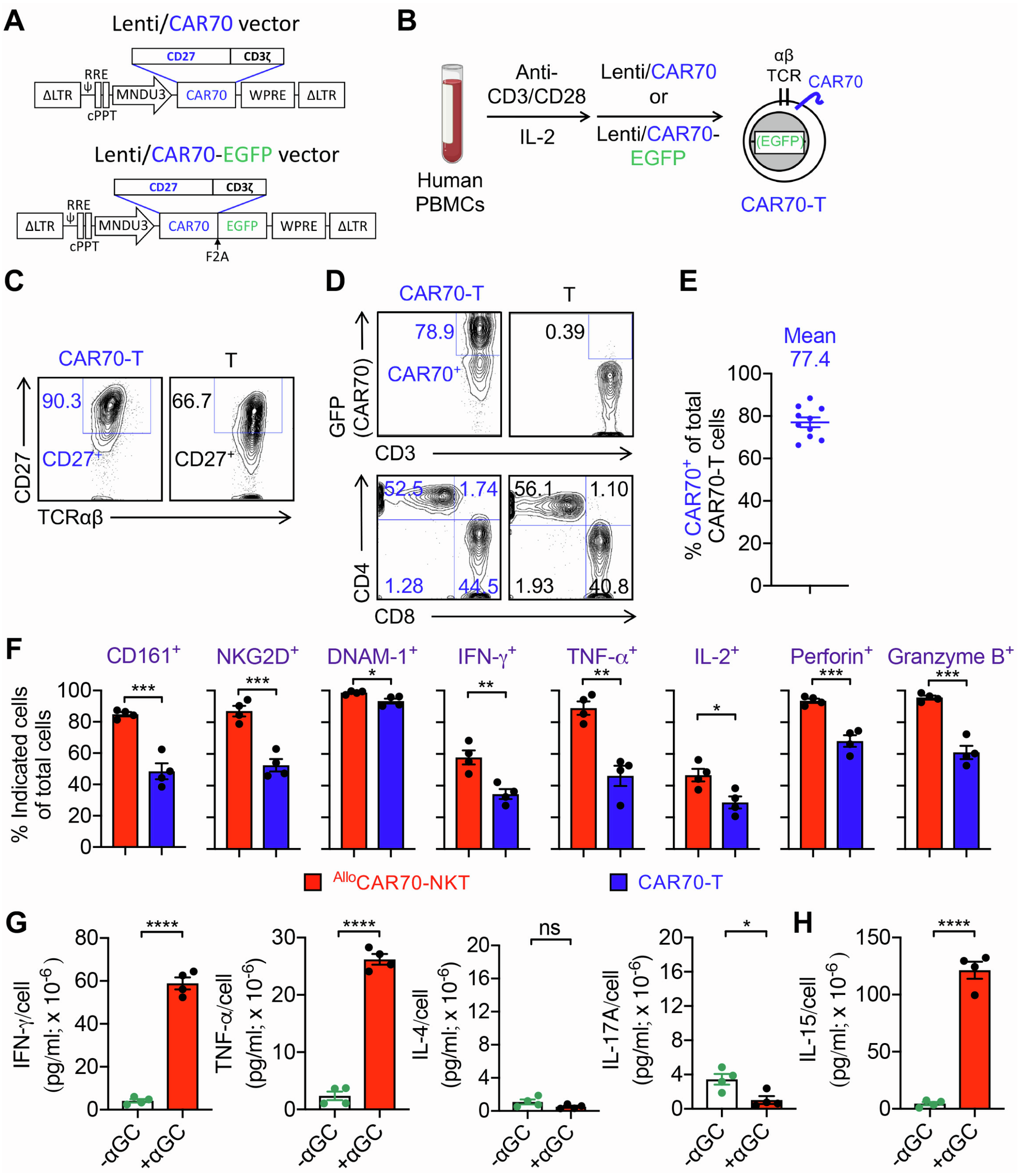

Figure S2. Generation of conventional CAR70-T cells and antigen response of ^{Allo}CAR70-NKT cells. Related to Figure 2.

(A) Schematics showing the design of Lenti/CAR70 and Lenti/CAR70-EGFP lentivectors. The CAR70 targets CD70 via its natural ligand CD27. EGFP, enhanced green fluorescent protein.

(B) Schematics showing the generation of CAR70-T cells.

(C) FACS detection of CD27 expression on CAR70-T and non CAR70-engineered T cells. Note that CD27 is endogenously expressed on T cells, making it challenging to use CD27 as a definitive marker for CAR expression.

(D) FACS detection of CAR70 (identified as GFP) and CD4/CD8 expressions on CAR70-T cells.

(E) CAR70 expression on CAR70-T cells (n = 10; n indicates different PBMC donors).

(F) FACS analyses of surface marker expression and intracellular marker production by ^{Allo}CAR70-NKT and conventional CAR70-T cells (n = 4; n indicates different donors). Data are related to main Figure 2K.

(G and H) Antigen responses of ^{Allo}CAR70-NKT cells. Data are related to main Figures 2L-2O. (G) ELISA measurements of effector cytokine (IFN-γ, TNF-α, IL-4, and IL-17A) levels in the culture supernatants collected on day 5 (n = 4). Note that the number was calculated by dividing the total amount of cytokines produced by the exact number of ^{Allo}CAR70-NKT cells, reflecting the cytokine production capacity of each individual cell. (H) ELISA measurements of IL-15 levels in the culture supernatants collected at 48 hours (n = 4). Note that the number was calculated by dividing the total amount of IL-15 produced by the exact number of ^{Allo}CAR70-NKT cells.

Representative of 3 (F-H) and over 10 (A-E) experiments. Data are presented as the mean ± SEM. ns, not significant, *$p < 0.05$, **$p < 0.01$, ***$p < 0.001$, ****$p < 0.0001$ by Student's *t* test (F-H).

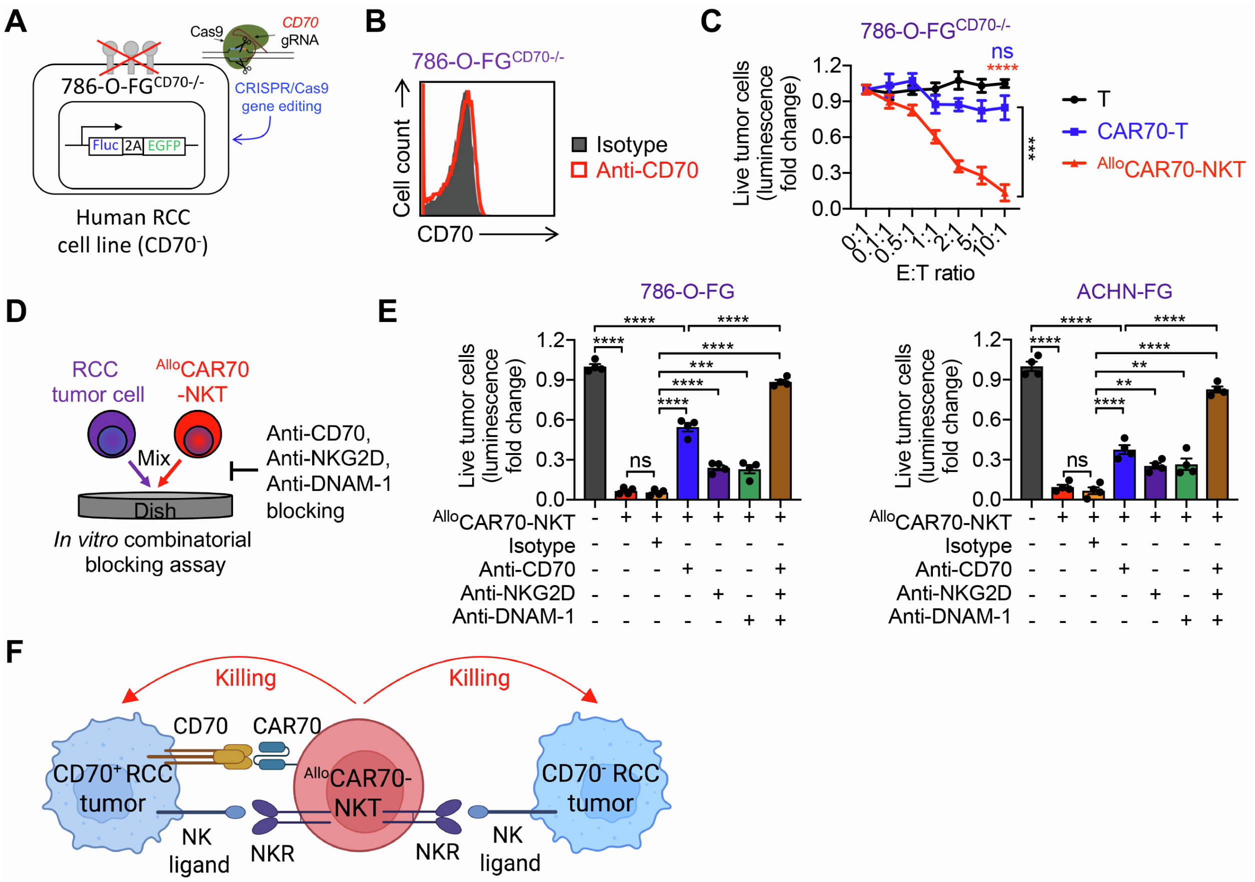

Figure S3. ***In vitro*** **tumor cell killing efficacy of** $^{\mathbf{Allo}}$**CAR70-NKT cells. Related to Figure 3.**

(A) Schematics showing the human RCC tumor cell line 786-O-FG$^{CD70-/-}$. gRNA, guide RNA; CRISPR, clustered regularly interspaced short palindromic repeats.

(B) FACS detection of CD70 expression on 786-O-FG$^{CD70-/-}$ cells.

(C) Tumor cell killing data at 24 h (n = 4).

(D and E) Studying the tumor cell killing mechanisms of ^{Allo}CAR70-NKT cells mediated by CAR and NKRs (i.e., NKG2D and DNAM-1) using an *in vitro* combinatorial blocking assay. (D) Experimental design. (E) Tumor cell killing data at 24 h (E:T ratio = 1:1; n = 4).

(F) Diagram showing that ^{Allo}CAR70-NKT cells mediate tumor killing through both CAR-dependent and NKR-dependent mechanisms: exerting dual CAR and NKR-mediated cytotoxicity against CD70^{+} RCC tumor cells, while relying on NKR-mediated killing for CD70^{-} RCC tumor cells.

Representative of 3 experiments. Data are presented as the mean ± SEM. ns, not significant, ***p < 0.001, ****p < 0.0001 by one-way ANOVA (C and E).

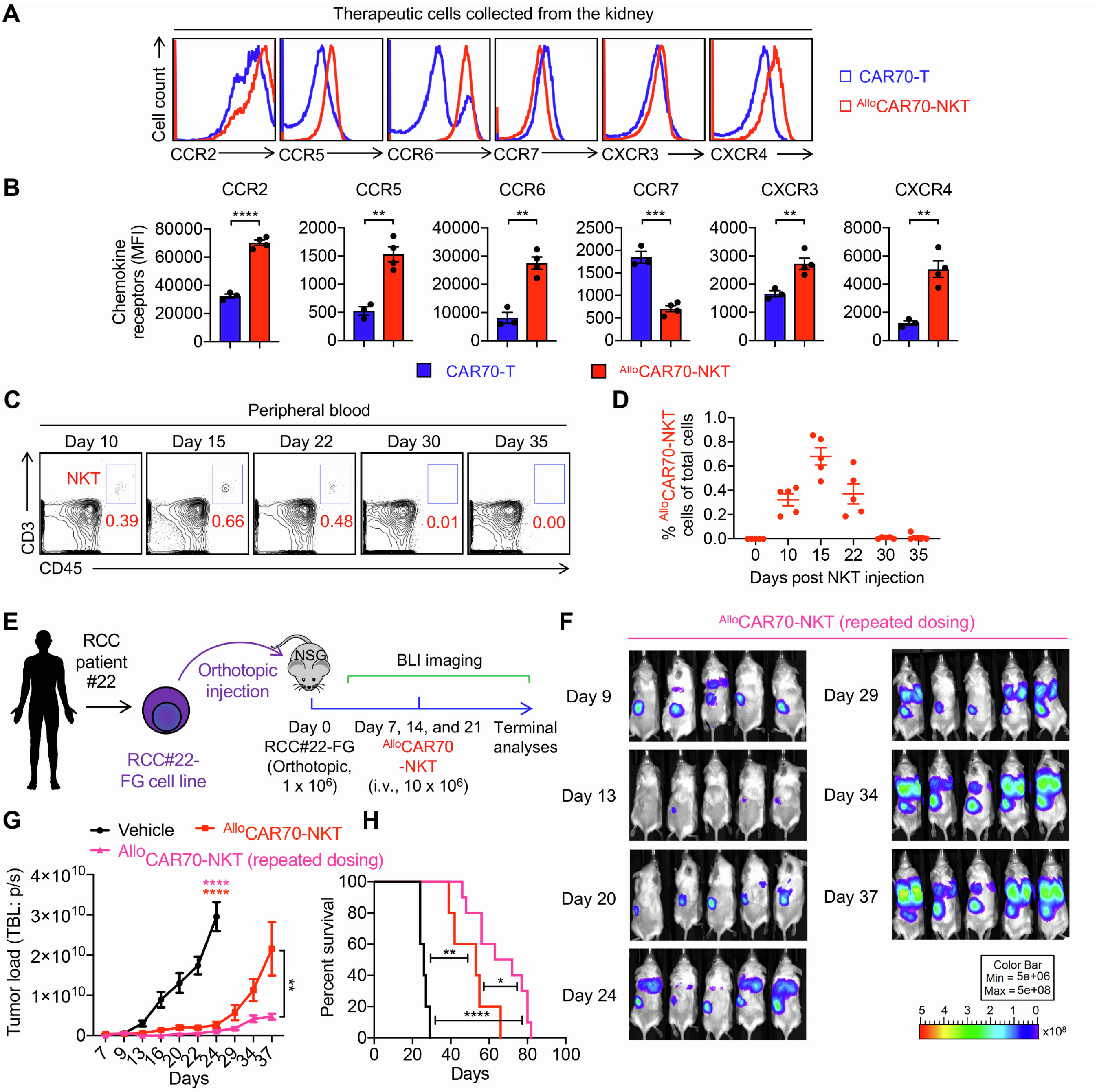

Figure S4. *In vivo* antitumor efficacy of ^{Allo}CAR70-NKT cells. Related to Figure 4.

(A and B) Chemokine receptor profiles of ^{Allo}CAR70-NKT cells and conventional CAR70-T cells. (A) FACS detection of the chemokine receptor expression in therapeutic cells collected from tumor sites of experimental mice on day 18. Data are related to main Figure 4A. (B) Quantification of (A) (n = 3-4).

(C and D) Studying the *in vivo* persistence of ^{Allo}CAR70-NKT cells by peripheral blood monitoring. (C) FACS plots showing the detection of ^{Allo}CAR70-NKT cells in the peripheral blood of experimental mice over time. (D) Quantification of (C) (n = 5).

(E-H) Studying the *in vivo* antitumor efficacy of ^{Allo}CAR70-NKT cells using an RCC PDX orthotopic mouse model and a repeated dosing strategy. (E) Experimental design. The repeated dosing group included a total of 10 mice from two independent experiments. (F) BLI images showing the presence of tumor cells in experimental

mice over time. Data from 5 representative mice are presented here. (G) Quantification of (F) (n = 5-10). (H) Kaplan–Meier survival curves of experimental mice over time (n = 5-10). The data for the Vehicle and ^{Allo}CAR70-NKT groups were also presented in the main Figures 4A-4D.

Representative of 2 experiments. Data are presented as the mean ± SEM. ns, not significant, *p < 0.05, **p < 0.01, ***p < 0.001, ****p < 0.0001 by Student's *t* test (B and G-Day 37), one-way ANOVA (G-Day 24), or log rank (Mantel-Cox) text adjusted for multiple comparisons (H).

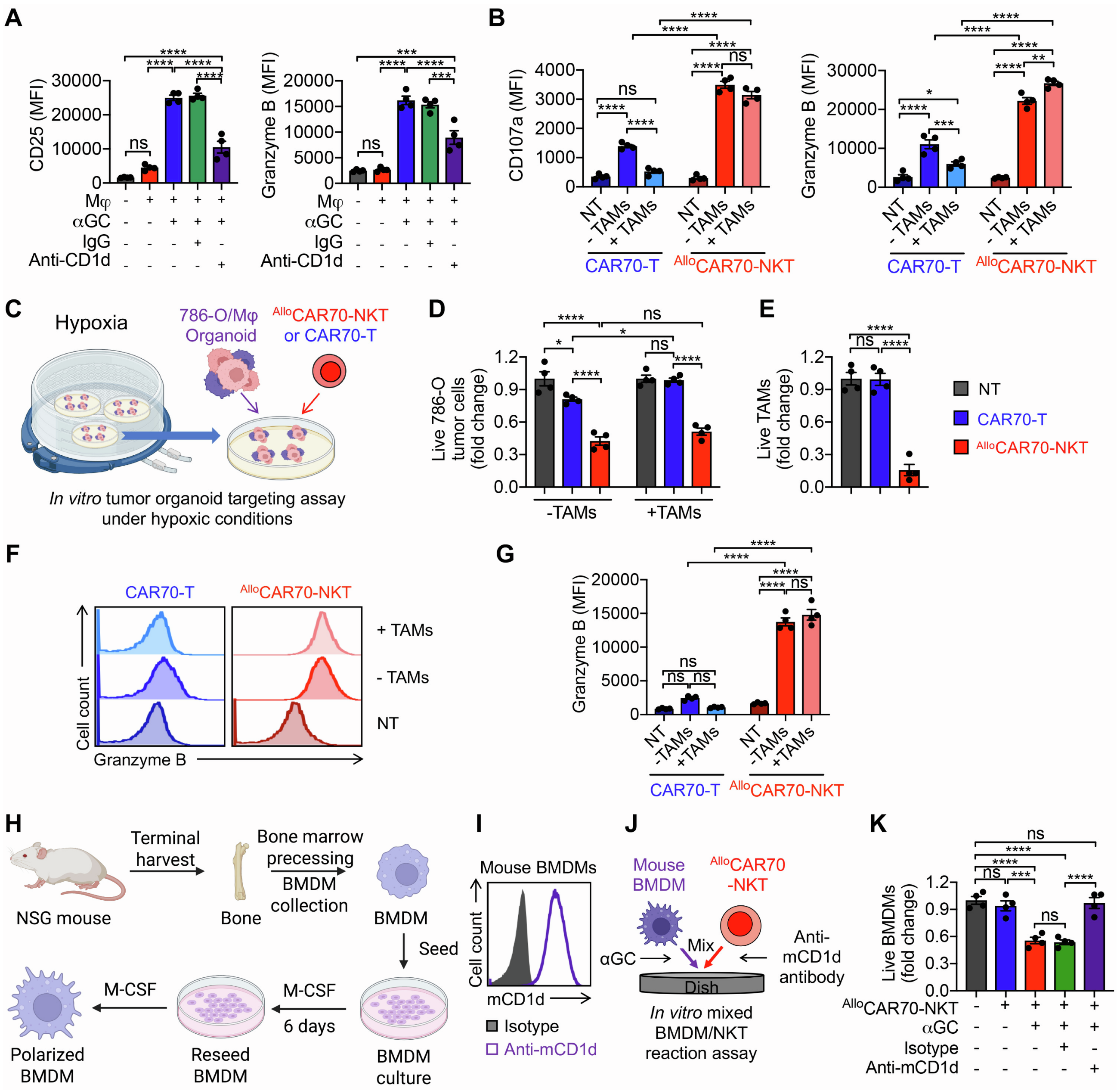

Figure S5. RCC TME targeting by ^{Allo}CAR70-NKT cells. Related to Figure 6.

(A and B) FACS analyses of surface effector marker (i.e., CD25) expression and intracellular cytotoxic molecule (i.e., Granzyme B) production in ^{Allo}CAR70-NKT cells, in the *in vitro* mixed macrophage/NKT reaction assay (A; related to Figures 6D-6H) and *in vitro* tumor organoid targeting assay (B; related to Figures 6I-6M) (n = 4).

(C-G) Studying ^{Allo}CAR70-NKT cells targeting of RCC TME using 786-O/macrophage co-culture organoid models under hypoxia conditions. (C) Experimental design. (D) Tumor cell killing data at 24 h (n = 4). (E) TAM killing data at 24 h (n = 4). (F) FACS detection of cytotoxic molecules (i.e., Granzyme B) production in the indicated therapeutic cells. (G) Quantification of (F) (n = 4).

(H-K) Studying ^{Allo}CAR70-NKT cells targeting of mouse BMDMs via mouse CD1d recognition. BMDM, bone marrow-derived macrophage. (H) Diagram showing the generation of mouse BMDMs. (I) FACS plots showing

the mouse CD1d expression on BMDMs. (J) Experimental design to study ^{Allo}CAR70-NKT cells targeting of mouse BMDMs using an *in vitro* mixed BMDM/NKT reaction assay. (K) BMDM killing data at 24 h (n = 4). Representative of 3 experiments. Data are presented as the mean ± SEM. ns, not significant, *p < 0.05, **p < 0.01, ***p < 0.001, ****p < 0.0001 by one-way ANOVA (A, B, D, E, G, and K).

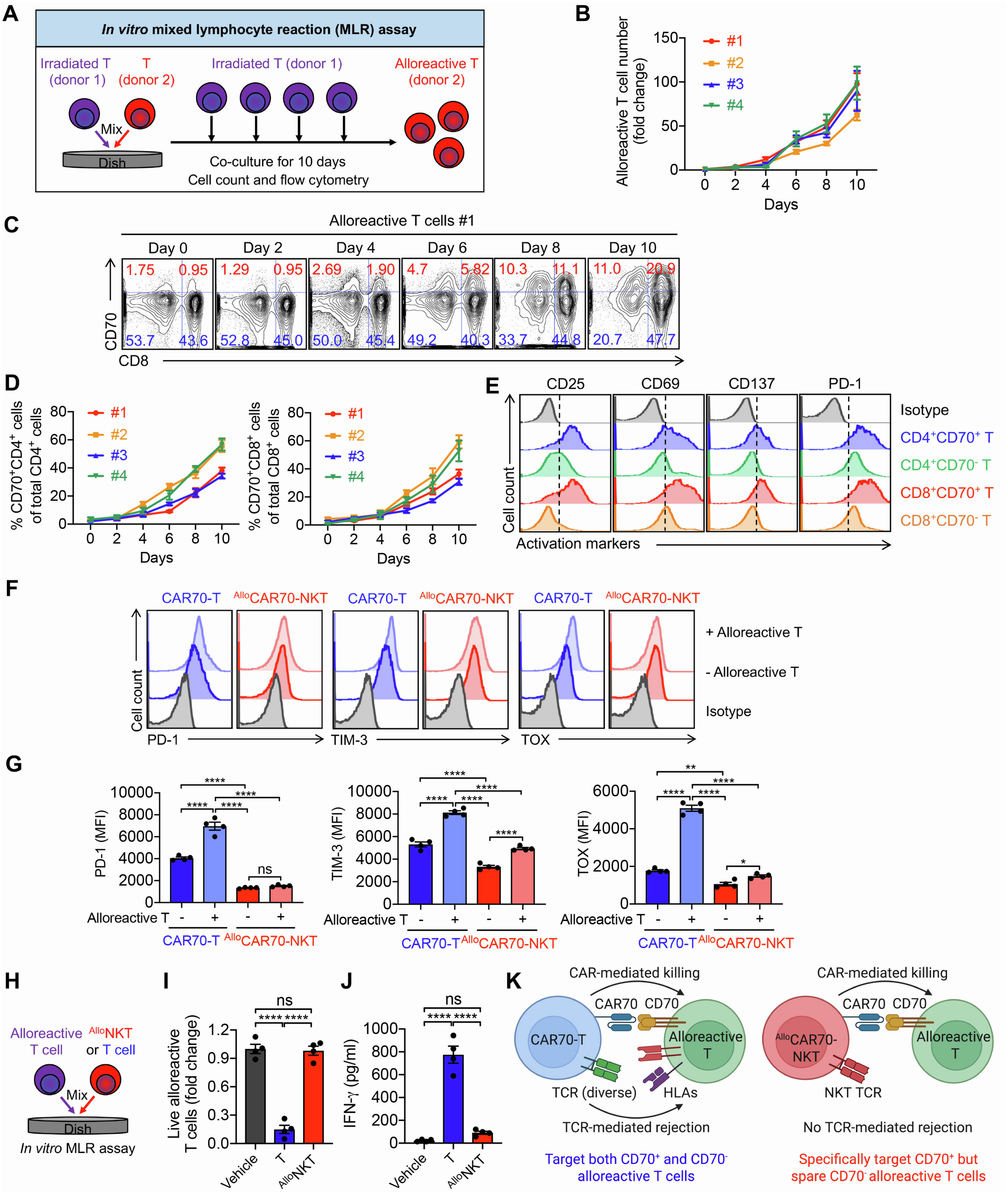

Figure S6. Alloreactive T cell targeting by ^{Allo}CAR70-NKT cells. Related to Figure 7.

(A-E) Generation and characterization of alloreactive T cells. (A) Diagram showing the generation of alloreactive T cells from healthy donor PBMCs. (B) Quantification of alloreactive T cell numbers throughout the *in vitro* culture period (n = 3). Alloreactive T cells from 4 PBMC donors were included. (C) FACS monitoring the CD70 expression on alloreactive T cells. (D) Quantification of CD70^{+} cells within CD4^{+} and

$CD8^+$ T cell subsets (n = 3). Alloreactive T cells from 4 PBMC donors were included. (E) FACS detection of T cell activation marker expression on the indicated cells.
(F and G) Exhaustion marker expression on ^{Allo}CAR70-NKT cells. (F) FACS plots showing the exhaustion marker expression on ^{Allo}CAR70-NKT cells in peripheral blood from experimental mice. The data are related to main Figure 7E. (G) Quantification or (F) (n = 3).
(H-K) Studying alloreactive T cell targeting by ^{Allo}NKT cells via TCR-mediated rejection. Conventional T cells were included as a control. (H) Experimental design. (I) Alloreactive T cell killing data at 24 h (E:T ratio = 5:1; n = 4) (J) ELISA measurements of IFN-γ levels in the culture supernatants collected at 24 h (n = 4). (K) Schematic illustrating that CAR70-T cells target alloreactive T cells through both CAR- and TCR-mediated mechanisms, whereas ^{Allo}CAR70-NKT cells selectively eliminate only $CD70^+$ alloreactive T cells via CAR-mediated recognition.
Representative of 3 experiments. Data are presented as the mean ± SEM. ns, not significant, *$p < 0.05$, **$p < 0.01$, ****$p < 0.0001$ by one-way ANOVA (G, I, and J).

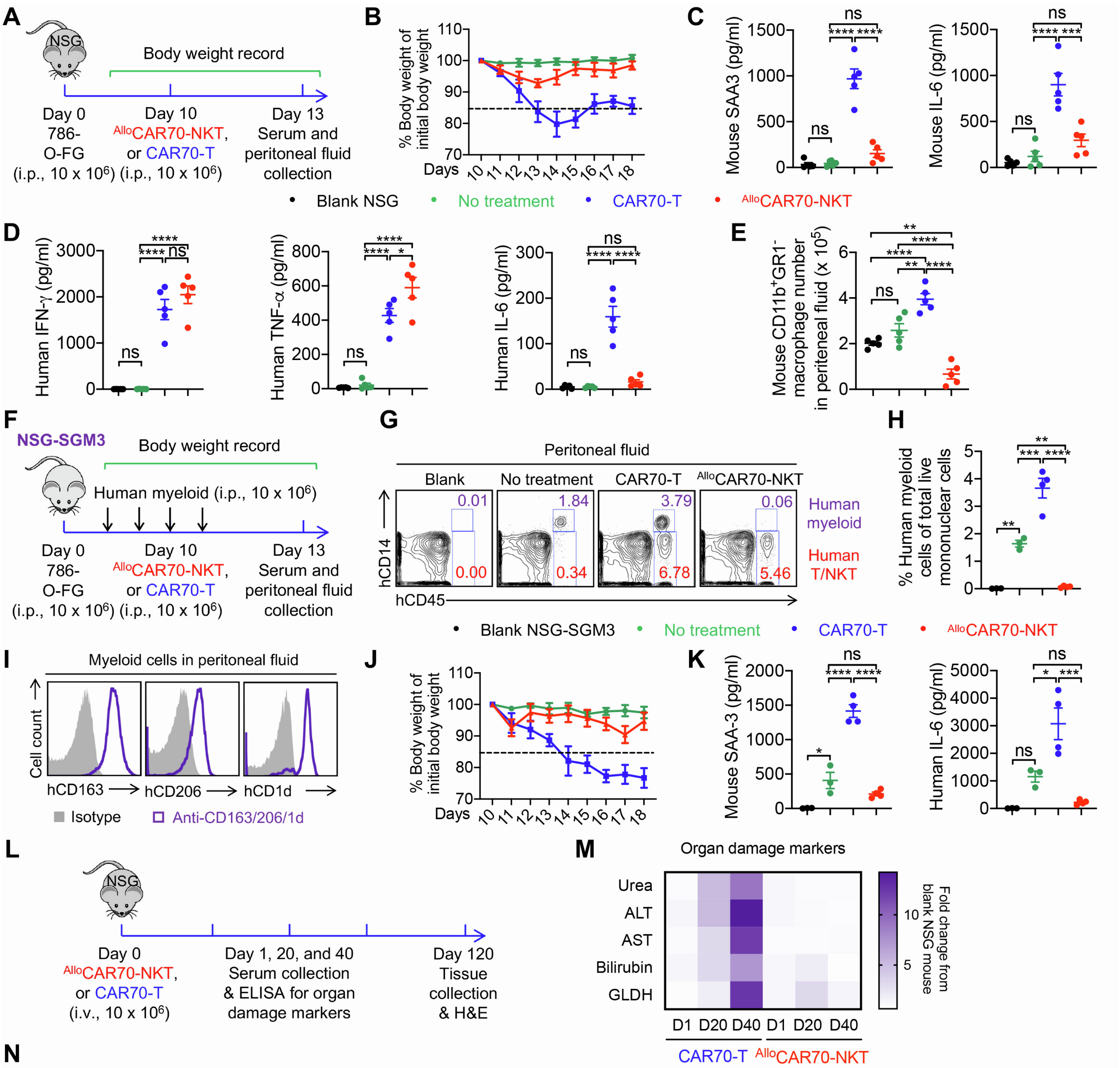

Group	Mouse ID	Pathologist's score																										
		Bone marrow			Spleen			Ileum			Pancreas			Kidney			Lung			Liver			Heart			Brain		
		Inf	HN	NHN	Inf	HN	NHN	Inf	HN	NHN	Inf	HN	NHN	Inf	HN	NHN	Inf	HN	NHN	Inf	HN	NHN	Inf	HN	NHN	Inf	HN	NHN
AlloCAR70-NKT	1	0	0	0	0	0	0	0	0	0	0	0	0	0	0	0	0	0	0	0	0	1	0	0	0	0	0	0
	2	0	0	0	0	0	0	0	0	0	0	0	0	0	0	0	0	0	0	0	0	0	0	0	0	0	0	0
	3	0	0	0	0	1	0	0	0	0	0	0	1	0	0	0	0	0	0	0	1	0	0	0	0	0	0	0
	4	0	0	0	0	0	0	0	0	0	0	0	0	0	0	0	0	0	0	0	0	0	0	0	0	0	0	0
	5	0	0	0	0	0	0	0	0	0	0	0	0	0	0	0	0	0	0	0	0	0	0	0	0	0	0	0
Blank NSG	6	0	0	0	0	0	0	0	0	0	0	0	0	0	0	0	0	0	0	0	0	0	0	0	0	0	0	0
	7	0	0	0	0	0	0	0	0	0	0	0	0	0	0	0	0	0	0	0	0	0	0	0	0	0	0	0
	8	0	0	0	0	0	0	0	0	0	0	0	0	0	0	0	0	0	0	0	0	0	0	0	0	0	0	0
	9	0	0	0	0	0	0	1	0	0	0	0	0	0	0	0	0	0	0	0	0	0	0	0	0	0	0	0
	10	0	0	0	0	0	0	0	0	0	0	0	0	0	0	0	0	0	0	1	0	0	0	0	0	0	0	0

Pathologist's score: 0 No abnormal findings; 1 Mild; 2 Moderate; 3 Severe

LEGEND: Inf: Inflammation; HN: Hematopoietic Neoplasm; NHN: Non-Hematopoietic Neoplasm

Figure S7. Safety study of AlloCAR70-NKT cells. Related to Figures 4, 5, and 7.

(A-E) Studying the CRS toxicity induced by AlloCAR70-NKT cells using a 786-O-FG human RCC xenograft NSG mouse model. (A) Experimental design. (B) Body weight of experimental mice over time (n = 5). (C)

ELISA analyses of mouse IL-6 and SAA3 in mouse serum (n = 5). (D) ELISA analyses of human cytokines (i.e., IFN-γ, TNF-α, and IL-6) in mouse serum (n = 5). (E) Quantification of mouse peritoneal macrophage numbers from the indicated samples. Mouse macrophages were identified as mouse $CD45^{+}CD11b^{+}GR1^{-}$ cells. (F-K) Studying the CRS toxicity induced by ^{Allo}CAR70-NKT cells using a 786-O-FG human RCC xenograft NSG-SGM mouse model. (F) Experimental design. Human $CD14^{+}$ myeloid cells were isolated via MACS sorting and intraperitoneally injected into experimental mice on days 3, 7, 10, and 12 to establish macrophage populations within the TME. (G) FACS plots showing the presence of human myeloid and T cells in the peritoneal fluids of experimental mice. (H) Quantification of (G) (n = 3-4). (I) FACS plots showing the expression of human CD163, CD206, and CD1d on myeloid cells isolated from the peritoneal fluid of experimental mice without therapeutic cell treatment. (J) Body weight of experimental mice over time (n = 3-4). (C) ELISA analyses of human IL-6 and SAA3 in mouse serum (n = 3-4).

(L-N) Studying the long-term organ toxicity induced by ^{Allo}CAR70-NKT cells using a human xenograft NSG mouse model. (L) Experimental design. (M) Organ damage markers (Urea, ALT, AST, Bilirubin, and GLDH) measured over time (n = 5). ALT, Alanine transaminase; AST, Aspartate Transaminase; GLDH, Glutamate Dehydrogenase. (N) Table showing organ damage scores for tissues collected from the experimental mice. Representative of 1 (N) and 2 (A-M) experiments. Data are presented as the mean ± SEM. ns, not significant, *$p < 0.05$, **$p < 0.01$, ***$p < 0.001$, ****$p < 0.0001$ by one-way ANOVA (C-E, H, and K).

Table S1. Primary RCC patient information. Related to Figure 1.

ID	UCLA code	Gender	Age	Pathology	Stage	Sample location
1	UCLA-RC-57	M	67	Clear cell RCC (ccRCC), WHO/ISUP (World Health Organization/International Society of Urological Pathology) grade 2	T1a	Primary renal
2	UCLA-RC-59	M	57	Leiomyosarcoma, FNCLCC (Federation Nationale des Centres de Lutte Contre le Cancer) grade 3	T4	Primary renal
3	UCLA-RC-60	M	70	Papillary RCC, WHO/ISUP grade 3	T1a	Transplanted kidney
4	UCLA-RC-63	M	60	ccRCC, WHO/ISUP grade 3	T2a	Primary renal
5	UCLA-RC-69	M	70	ccRCC, WHO/ISUP grade 3	T3a	Primary renal
6	UCLA-RC-64	M	74	ccRCC, WHO/ISUP grade 4	T3a	Primary renal
7	UCLA-RC-68	M	60	ccRCC, WHO/ISUP grade 3	T3a	Primary renal
8	UCLA-RC-65	M	25	ccRCC, WHO/ISUP grade 2	T1b	Primary renal
9	UCLA-RC-66	F	77	Low grade oncocytic tumor	T1a	Primary renal
10	UCLA-RC-67	M	85	ccRCC, WHO/ISUP grade 3	T3a N0 M1	Primary renal
11	UCLA-RC-71	M	71	ccRCC, WHO/ISUP grade 3	T1b	Primary renal
12	UCLA-RC-73	F	42	ccRCC, WHO/ISUP grade 2	T1b	Primary renal
13	UCLA-RC-74	M	49	ccRCC, WHO/ISUP grade 3	T1a	Primary renal
14	UCLA-RC-75	M	87	Papillary RCC, WHO/ISUP grade 3	T3a N2 M1	Primary renal
15	UCLA-RC-77	F	48	ccRCC, WHO/ISUP grade 3	T1a	Primary renal
16	UCLA-RC-36	F	21	ccRCC, WHO/ISUP grade 2	T1a	Primary renal (know VHL patient)
17	UCLA-RC-21	M	64	ccRCC, WHO/ISUP grade 2	T1a	Primary renal
18	UCLA-RC-24	F	69	ccRCC, WHO/ISUP grade 3	T2	Primary renal
19	UCLA-RC-34	F	66	Papillary RCC, WHO/ISUP grade 2	T1a	Primary renal
20	UCLA-RC-25	F	54	ccRCC, WHO/ISUP grade 2	T1a	Primary renal
21	UCLA-RC-22	M	59	ccRCC, WHO/ISUP grade 4	T3a N0 M2	Primary renal
22	UCLA-RC-18	M	61	ccRCC, WHO/ISUP grade 3	T2	Primary renal
